# Regioselective synthesis, structural investigation and binding behavior of 6-aroyl-7-aryl-5-methyl-4,7-dihydro-[1,2,4]triazolo[1,5-*a*]pyrimidines with BSA using various spectroscopic and *in silico* methods

**DOI:** 10.1039/d5ra09673a

**Published:** 2026-03-10

**Authors:** Ranjana Aggarwal, Manisha Sharma, Garima Sumran, Suresh Kumar, Parvin Kumar

**Affiliations:** a Department of Chemistry, Kurukshetra University Kurukshetra-136119 Haryana India ranjana67in@yahoo.com ranjanaaggarwal67@gmail.com +91-9896740740; b Council of Scientific and Industrial Research (CSIR) HQ Vigyan Suchna Bhawan, 14, Satsang Vihar Marg New Delhi-110067 India; c Department of Chemistry, D. A. V. College (Lahore) Ambala City Haryana 134 003 India; d Department of Chemistry, SUS Govt. College Matak Majri Karnal Haryana, 132041 India

## Abstract

A series of 6-aroyl-7-aryl-5-methyl-4,7-dihydro-[1,2,4]triazolo[1,5-*a*]pyrimidines 5 was synthesized regioselectively *via* a one-pot multicomponent reaction between β-diketone 1, aromatic aldehyde 2, and 3-amino-1*H*-1,2,4-triazole 3. The regioisomeric structure of the newly synthesized compounds 5 was unambiguously determined using ^1^H NMR, ^13^C NMR, and rigorous multinuclear 2D-NMR spectroscopy [^1^H–^13^C] HMBC, [^1^H–^13^C] HSQC and [^1^H–^15^N] HMBC. The remarkable features of this protocol are high yields, operational simplicity, use of commercially available reagents and broad substrate scope. The interactions of selected compounds (5f, 5m and 5t) with bovine serum albumin (BSA) were studied by UV-vis spectroscopy, steady-state fluorescence, and molecular docking. The results indicated that compound 5t could effectively quench the intrinsic fluorescence of BSA *via* a static quenching process. Competitive binding studies using site markers demonstrated that compound 5t binds to site I of BSA. Binding constants for [1,2,4]triazolo[1,5-*a*]pyrimidines show that the affinity of 5t binding to BSA is stronger than that of 5f and 5m.

## Abstract 2

Regioselective synthesis and characterization of 6-aroyl-7-aryl-5-methyl-4,7-dihydro [1,2,4]triazolo[1,5-*a*]pyrimidines identified that compound 5t has the strongest BSA binding *via* site I static quenching mechanism.

## Graphical abstract

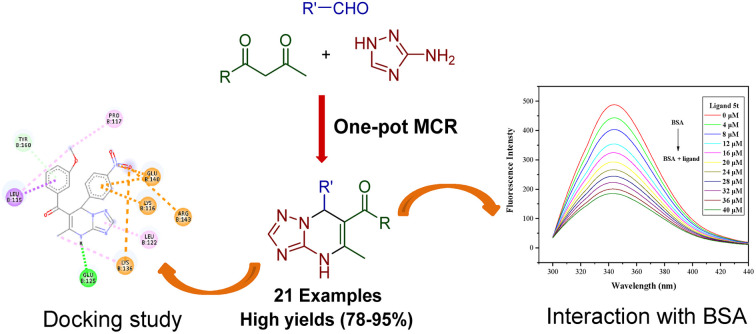

## 1. Introduction

**Fig. 1:**
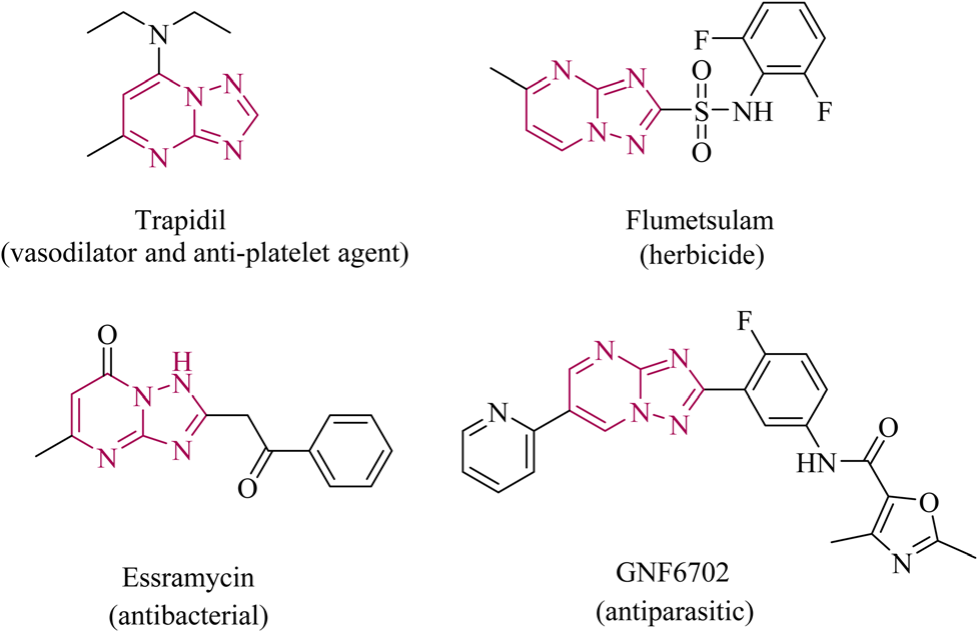
Marketed drugs with the [1,2,4]triazolo[1,5-*a*]pyrimidine nucleus.

[1,2,4]Triazolo[1,5-*a*]pyrimidine and its derivatives have profound applications in both medical and agricultural fields because of their varied biological activities and synthetic versatility.^1,2^ This system exhibits favourable physicochemical characteristics such as planarity, hydrogen-bonding capability, and multiple nitrogen donor sites along with multiple substitution sites (5,6,7) at the pyrimidine ring and the capacity to interact with diverse biological targets. Due to its structural similarity with purines, this scaffold has been extensively investigated as a possible isosteric replacement for the purine ring.^3^ [1,2,4]Triazolo[1,5-*a*]pyrimidines have a broad spectrum of biological activities including antiviral,^4^ antimicrobial,^5,6^ antiparasitic,^7^ anticancer,^8^ anti-inflammatory,^9^ and anticonvulsant properties.^10^ These compounds have emerged as corrosion inhibitors^11^ and promising PDE2A inhibitors with potential in treating Alzheimer's disease and other neurodegenerative disorders.^12–14^ The [1,2,4]triazolo[1,5-*a*]pyrimidine scaffold is a key structural motif in several clinically and pharmacologically relevant drugs, including Trapidil (a vasodilator/antiplatelet agent),^15^ Flumetsulam (a herbicide),^16^ Essramycin (an antibacterial agent),^17^ and GNF6702 (a broad-spectrum antiprotozoal proteasome inhibitor),^18^ highlighting its broad therapeutic versatility (Fig. 1).

Proteins serve as primary molecular targets owing to their pivotal roles in cellular signaling and regulation and their ability to form drug–protein interactions enabling selective modulation of biochemical pathways for therapeutic effects.^19^ Bovine serum albumin (BSA) is a globular protein widely used in biochemical and pharmaceutical research due to its structural similarity to human serum albumin (HSA) and its ability to bind a variety of ligands.^20^ BSA, a type of serum albumin, plays a crucial role in maintaining blood pH, oncotic pressure and the transport of endogenous and exogenous compounds, including fatty acids, drugs, and other metabolites. BSA comprises 583 amino acids and is structured into three homologous α-helical domains.^21^ It contains three major drug-binding sites: site I (subdomain IIA), site II (subdomain IIIA), and site III (subdomain IB), each exhibiting distinct affinities for ligands. BSA serves as a valuable model for studying drug–protein interactions, pharmacokinetics, and drug delivery mechanisms due to its wide availability, low cost, stability, and unusual binding properties.

Two main synthetic approaches have been developed for the construction of [1,2,4]triazolo[1,5-*a*]pyrimidines. The first involves the annulation of a triazole ring onto a pyrimidine precursor,^22^ while the second and most commonly used approach involves the annulation of a pyrimidine moiety onto a triazole core.^23^ Literature reports the synthesis of [1,2,4]triazolo[1,5-*a*]pyrimidines *via* a cyclocondensation reaction of 3-amino-1,2,4-triazole with 1,3-dicarbonyl compounds or α,β-unsaturated carbonyl compounds.^24^ Multicomponent reactions (MCRs) are of significant importance in synthetic chemistry due to their operational simplicity, high atom economy, and capacity to rapidly generate structurally diverse and complex scaffolds from readily available starting materials in a single step, thereby minimizing time, resource consumption, and byproduct formation. Therefore, another efficient synthetic approach to triazolo[1,5-*a*]pyrimidines is Biginelli-like three-component heterocyclization of 3-amino-1,2,4-triazole with aldehyde and active methylene compounds (β-keto ester,^25^ cyclohexane-1,3-dione,^26^ β-ketonitrile,^27^ malononitrile,^28^ and acetoacetanilide^29^). Four-component condensation of 1,3-indanedione, aromatic aldehyde, 5-amino-4*H*-1,2,4-triazol-3-thiol and phenacyl bromide has also been reported to afford triazolo[1,5-*a*]pyrimidines.^30^ El-Deeb *et al.* reported the synthesis of 2-[(4-chlorophenyl)-7-(4-methoxyphenyl)-5-methyl-4,7-dihydro-[1,2,4]triazolo[1,5-*a*]pyrimidin-6-yl]phenylmethanone in 27% yield *via* a multicomponent reaction of 3-(4-chlorophenyl)-4*H*-[1,2,4]triazol-5-amine, 4-methoxybenzaldehyde, and benzoylacetone under reflux in glacial acetic acid for 36 h.^31^ However, to the best of our knowledge, the reaction of 3-amino-1*H*-1,2,4-triazole with aldehydes and unsymmetrical aryl 1,3-diketones has not yet been reported in the literature. Accordingly, this work was designed to synthesize triazolopyrimidine derivatives bearing diverse substitutions at the 5,6,7-positions by varying the aromatic aldehydes and 1,3-diketone components.

Keeping in view the biological importance of [1,2,4]triazolo[1,5-*a*]pyrimidines and following our ongoing work on the multicomponent synthesis of novel bioactive heterocycles,^32,33^ we herein report a one-pot multicomponent reaction for regioselective synthesis of 6-aroyl-7-aryl-5-methyl-4,7-dihydro-[1,2,4]triazolo[1,5-*a*]pyrimidine derivatives (5) using 3-amino-1*H*-1,2,4-triazole, aromatic aldehydes and unsymmetrical 1,3-diketones. The exact structure of the regioisomer was identified unambiguously by multinuclear 2D-NMR spectroscopy. Furthermore, the binding properties of the synthesized [1,2,4]triazolo[1,5-*a*]pyrimidine derivatives with BSA were investigated using an *in silico* molecular docking method and various spectroscopic techniques, namely UV-visible, steady-state fluorescence and competitive displacement assays. These studies are pivotal for the advancement in developing novel pharmaceutical agents and the optimization of therapeutic compounds.

## 2. Results and discussion

### 2.1. Chemistry

**Scheme 1:**
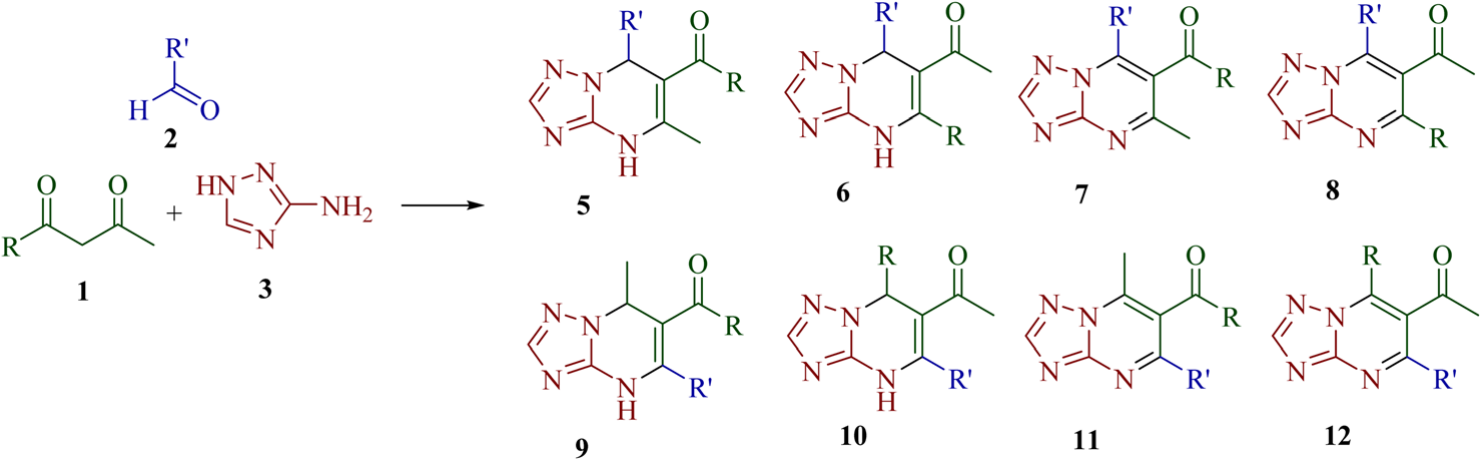
Structure of possible [1,2,4]triazolo[1,5-*a*]pyrimidine derivatives 5–12*via* the condensation of 3-amino-1*H*-1,2,4-triazole, aldehydes and unsymmetrical 1,3-diketones.

The synthetic strategy for functionalized [1,2,4]triazolo[1,5-*a*]pyrimidine derivatives through Biginelli-like condensation between 3-amino-1*H*-1,2,4-triazole 3, aromatic aldehyde 2 and unsymmetrical 1,3-diketones 1 is shown in Scheme 1. The presence of various nucleophilic sites on 3-amino-1*H*-1,2,4-triazole (3) and distinct electrophilic centers in unsymmetrical 1,3-diketones (1) could lead to the possibility of at least eight structurally diverse products (4,7-dihydrotriazolo[1,5-*a*]pyrimidine- and aromatized triazolo[1,5-*a*]pyrimidine derivatives 5–12). To study the regioselectivity pattern, a model three-component reaction of 3-amino-1*H*-1,2,4-triazole 3 (1.0 mmol), 1-phenylbutane-1,3-dione 1a (1.0 mmol) and benzaldehyde 2a (1.0 mmol) was initially investigated in a variety of polar protic and aprotic solvents (ethanol, water, tetrahydrofuran, acetonitrile and DMF). The results are depicted in Table 1. The reaction did not proceed at room temperature in water or DMF, even after stirring for 5 h (entries 3 and 8). Under reflux, most of the tested solvents resulted either in complex mixtures or in the formation of a gummy, non-isolable mass (entries 1, 2, 4–6, 9, and 10). Notably, the reaction in acetonitrile in the presence of I_2_ afforded the product 5a in a modest 30% yield (entry 7). Solvent-free conditions were also examined. No reaction occurred at room temperature (entry 11), while heating to 150 °C for 8 h led to the formation of a gummy mass instead of the desired product (entry 12).

**Table 1:** Screening of solvents and optimization of reaction conditions^a^

Entry	Solvent	Temperature	Time	Yield^b^ (%)
1	EtOH	Reflux	9 h	—c
2	EtOH	Reflux	24 h	—c
3	Water	rt	5 h	NR^d^
4	Water	Reflux	12 h	Gummy mass
5	THF	Reflux	9 h	—c
6	MeCN	Reflux	9 h	—c
7	MeCN-I_2_	Reflux	9 h	30
8	DMF	rt	5 h	NR^d^
9	DMF	Reflux	8–9 h	Gummy mass
10	DMF-KOH	Reflux	8–9 h	Gummy mass
11	Solvent-free	rt	5 h	NR^d^
12	Solvent-free	150 °C	8 h	Gummy mass

a Reagents and conditions: 1-phenylbutane-1,3-dione (1a, 1.0 mmol), benzaldehyde (2a, 1.0 mmol), 3-amino-1*H*-1,2,4-triazole (3, 1.0 mmol) and an appropriate solvent (10.0 mL). rt = room temperature.

b Isolated yield.

c Complex mixture obtained.

d NR = no reaction.

The structure of the isolated product 5a was confirmed using spectroscopic data. The IR spectrum of compound 5a displayed characteristic absorption bands at 3466 cm^−1^ and 1628 cm^−1^ corresponding to the amino (NH) group and carbonyl group stretching vibrations, respectively, indicating the product formation. The ^1^H NMR spectrum of 5a displayed a sharp singlet signal of three protons intensity at *δ* = 1.82 ppm attributed to the methyl group, three singlet signals of one proton intensity at *δ* = 6.42, 7.68 and 10.73 ppm corresponding to 2-H, 7-CH and NH proton, respectively, besides signals for the aromatic rings. The ^13^C NMR spectrum confirmed this, revealing two signals in the aliphatic region at *δ* = 19.56 and 61.24 ppm and one in the aromatic region at *δ* = 194.95 ppm assigned to methyl carbon, C-7 and the carbonyl carbon, respectively, along with a set of twelve desired carbon signals in the aromatic region. Furthermore, HRMS (ESI) *m*/*z* calculated for C_19_H_16_N_4_O is 316.1324 and was observed at 317.1328 for (M + H)^+^, confirming the successful condensation of reactants to afford targeted [1,2,4]triazolo[1,5-*a*]pyrimidine 5a.

**Scheme 2:**
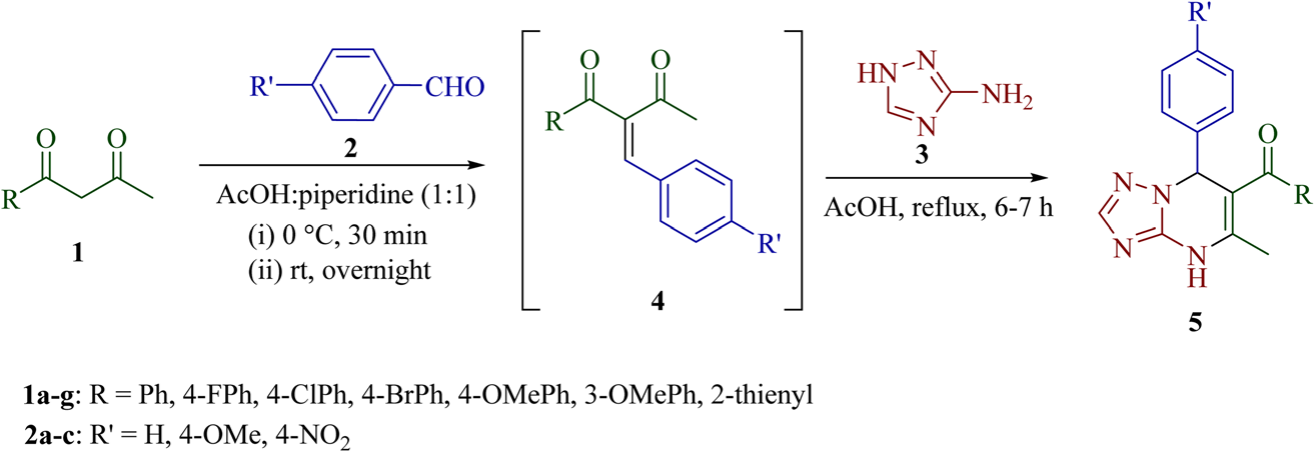
Regioselective synthesis of [1,2,4]triazolo[1,5-*a*]pyrimidines 5.

To improve the yield of target product, an alternative one-pot step-wise synthetic strategy was explored. In this approach, an equimolar mixture of 1-phenylbutane-1,3-dione 1 and benzaldehyde 2 was stirred in the presence of an acetic acid–piperidine catalytic system to generate the intermediate (Z)-2-benzylidene-1-phenylbutane-1,3-dione 4, followed by the condensation of 4 with 3-amino-1*H*-1,2,4-triazole 3 in acetic acid under reflux for 6–7 h to afford 5-methyl-7-phenyl-4,7-dihydro-[1,2,4]triazolo[1,5-*a*]pyrimidin-6-yl)(phenyl)methanone 5a regioselectively in 80% yield (Scheme 2). The identity of intermediate 4 was confirmed, in the case of 4a, by comparison of its melting point and spectral data with the reported literature data.^34^

The scope of the described sequential multicomponent reaction was explored by using various aromatic aldehydes and unsymmetrical β-diketones (Table 2). All the substrates having electron-withdrawing or electron-donating groups on the phenyl ring of aromatic benzaldehydes and β-diketones smoothly gave a diverse range of [1,2,4]triazolo[1,5-*a*]pyrimidine derivatives (5b–u) with high regioselectivity in good yields (78–95%). High yields were obtained when the aldehyde contained an electron-donating substituent (4-OMe) in the aromatic ring in comparison to the electron-withdrawing group (4-NO_2_).

**Table 2:** Substrate scope^a^^,^^b^

S. No.	Diketone (1)	Aldehyde (2)	3-Amino-1*H*-1,2,4-triazole	Triazolopyrimidines	Yield%
	R	R′	(3)	(5)	
1	Ph	H	3	5a	80
2	4-FPh	H	3	5b	82
3	4-ClPh	H	3	5c	80
4	4-BrPh	H	3	5d	81
5	4-OMePh	H	3	5e	86
6	3-OMePh	H	3	5f	82
7	2-Thienyl	H	3	5g	79
8	Ph	4-OMe	3	5h	86
9	4-FPh	4-OMe	3	5i	92
10	4-ClPh	4-OMe	3	5j	90
11	4-BrPh	4-OMe	3	5k	88
12	4-OMePh	4-OMe	3	5l	95
13	3-OMePh	4-OMe	3	5m	92
14	2-Thienyl	4-OMe	3	5n	82
15	Ph	4-NO_2_	3	5o	80
16	4-FPh	4-NO_2_	3	5p	83
17	4-ClPh	4-NO_2_	3	5q	82
18	4-BrPh	4-NO_2_	3	5r	80
19	4-OMePh	4-NO_2_	3	5s	89
20	3-OMePh	4-NO_2_	3	5t	85
21	2-Thienyl	4-NO_2_	3	5u	78

a Reaction conditions: unsymmetrical β-diketones 1 (1.0 mmol) and aromatic aldehyde 2 (1.0 mmol) were stirred in AcOH/piperidine (1 : 1) at 0 °C for 30 min, followed by stirring at room temperature overnight. Subsequently, 3-amino-1*H*-1,2,4-triazole 3 (1.0 mmol) was added, and the reaction mixture was refluxed in AcOH for 6–7 h.

b Isolated yields.

The structures of the newly synthesized compounds 5 were characterized using spectroscopic techniques including IR, ^1^H NMR, ^13^C NMR, and MS spectrometry, with all spectral data made available in the SI.

**Fig. 2:**
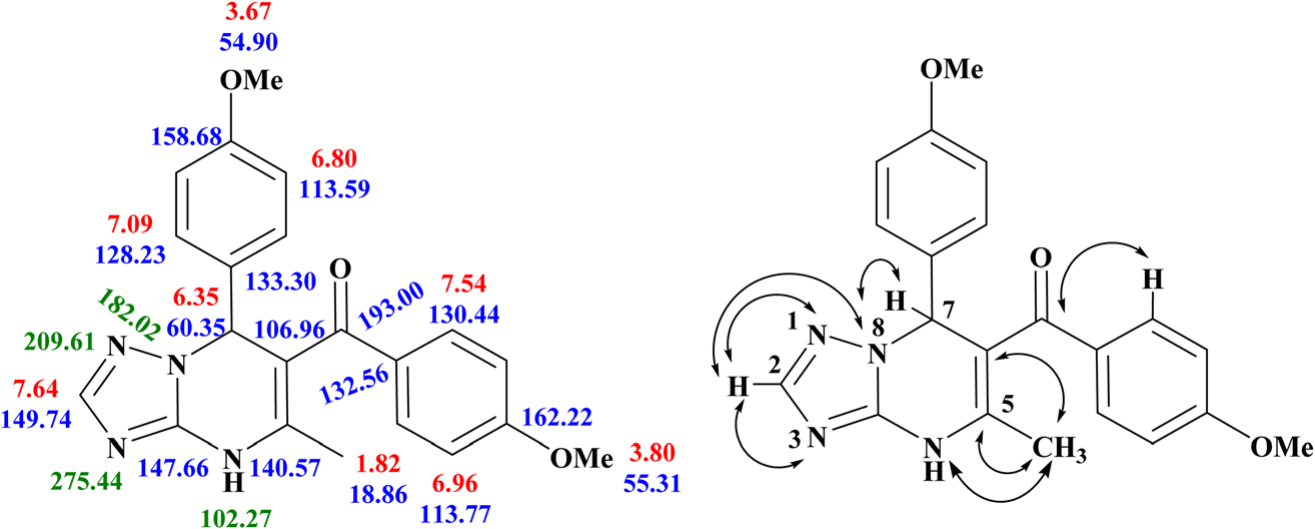
^1^H (in red), ^13^C (in blue) and ^15^N (in green) NMR spectral characteristics (chemical shifts *δ*, ppm) and key correlation in the 2D NMR spectra for compound 5l.

To unambiguously determine the correct regioisomeric structure of the isolated product, two-dimensional NMR experiments ([^1^H–^13^C] HMBC; [^1^H–^13^C] HSQC and [^1^H–^15^N] HMBC) were conducted. The 2D NMR correlation results and ^1^H, ^13^C and ^15^N chemical shifts for compound 5l are shown in Fig. 2, S1–S3 and Table 3.

**Table 3:** Assignment and chemical shifts in compound 5l

Chemical shifts (*δ* in ppm)	gs-HSQC correlation	gs-HMBC correlation	Assignments
193.00	—	7.54 (H2″/H6″)	CO
		6.35 (7-H)	
162.22	—	7.54 (H2″/H6″)	C4″
		6.96 (H3″/H5″)	
158.68	—	7.09 (H2′/H6′)	C4′
		6.80 (H3′/H5′)	
149.74	7.64 (2-H)	147.66 (C-3a)	C2
147.66	—	7.64 (2-H)	C-3a
140.57	—	1.82 (CH_3_)	C5
		6.35 (7-H)	
133.30		7.09 (H2′/H6′)	C1′
		6.80 (H3′/H5′)	
		6.35 (7-H)	
132.56		7.54 (H2″/H6″)	C1″
		6.96 (H3''/H5″)	
130.44	7.54 (H2''/H6″)	193.00 (C=O)	C″/C6″
		162.22 (C4″)	
		132.56 (C1″)	
128.23	7.09 (H2′/H6′)	158.68 (C4′)	(C2′/C6′)
		60.35 (C-7)	
113.77	6.96 (H3''/H5″)	162.22 (C4″)	(C″/C5″)
		132.56 (C1″)	
113.59	6.80 (H3′/H5′)	158.68 (C4′)	(C3′/C5′)
		133.30 (C1′)	
106.96		6.35 (7-H)	C6
60.35	6.35 (C–H)	133.30 (C1′)	C7
		128.23 (C2′/C6′)	
		140.57 (C5)	
		147.66 (C-3a)	
		193.00 (CO)	
		106.96 (C6)	
55.31	3.80 (4″-OCH_3_)	162.22 (C4″)	C4″-OCH_3_
54.90	3.67 (4′-OCH_3_)	158.68 (C4′)	C4′-OCH_3_
18.86	1.82 (5-CH_3_)	140.57 (C5)	C5–CH_3_
		106.96 (C6)	

The (^1^H–^13^C) HMBC spectrum of 5l exhibits cross-peaks of protons of methyl group (*δ* = 1.82 ppm) with C-5 (*δ* = 140.57 ppm) and C-6 (*δ* = 106.96 ppm), confirming the presence of a methyl substituent at the C-5 position of the pyrimidine nucleus. Furthermore, carbonyl carbon at *δ* = 193.0 ppm displays cross peaks with H2″/H6″ (*δ* = 7.54 ppm) protons of the aryl ring and C–H at 6.35 ppm (likely H at C-7), indicating the presence of a carbonyl carbon atom with an aryl/heteroaryl ring. Similarly, the (^1^H–^15^N) HMBC spectrum also shows a cross peak of methyl proton (*δ* = 1.82 ppm) with N-4 (*δ* = −102.27), thus confirming the presence of the methyl substituent at position-5 of [1,2,4]triazolo[1,5-*a*]pyrimidine. Furthermore, the N8 atom (*δ* = −182.02 ppm) shows correlation peaks with a proton at the C-7 position (*δ* = 6.35 ppm) and a methine proton (*δ* = 7.64) at the C-2 position. Lastly, the N-1 (*δ* = −209.61 ppm) and N-3 (*δ* = −275.44 ppm) atoms display cross peaks with methine protons (*δ* = 7.64) at the C-2 position. The possibility of the structure 6-acetyl-5,7-diphenyl-4,7-dihydro-[1,2,4]triazolo[1,5-*a*]pyrimidine 6 can be ruled out, as there is no cross peak of carbonyl carbon to methyl protons in the HMBC spectrum. Thus, from the above-mentioned 2D NMR evidence, the structure of the regioisomer 5l can unequivocally be determined as (4″-methoxyphenyl)(7-(4′-methoxyphenyl)-5-methyl-4,7-dihydro-[1,2,4]triazolo[1,5-*a*]pyrimidin-6-yl)methanone (Fig. 2).

**Scheme 3:**
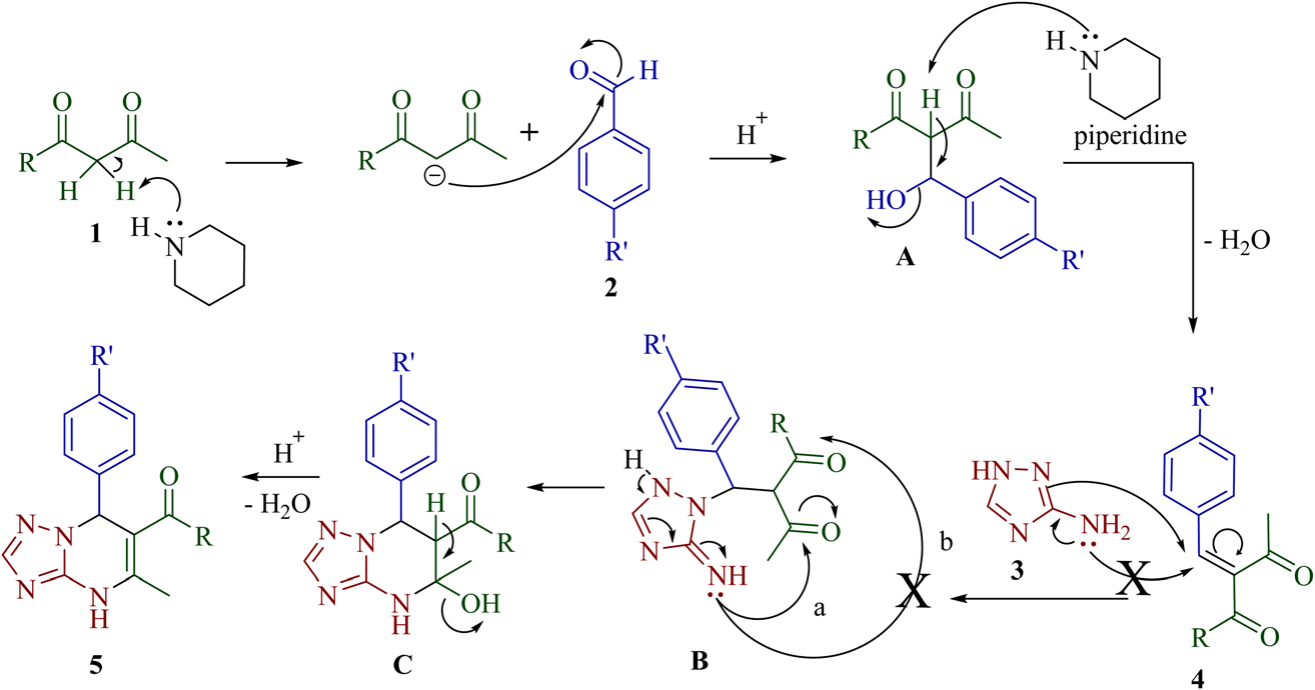
Plausible mechanism of the regioselective synthesis of the 4,7-dihydro[1,2,4]triazolo[1,5-*a*]pyrimidine derivatives 5.

The plausible mechanism for the regioselective synthesis of [1,2,4]triazolo[1,5-*a*]pyrimidine derivatives 5 is outlined in Scheme 3. Initially, piperidine acts as a Brønsted base and abstracts a proton from the active methylene group of β-diketone 1, generating a stabilized enolate intermediate.^35,36^ Concurrently, the aromatic aldehyde 2 is weakly activated under acidic conditions through the protonation of the carbonyl oxygen atom by acetic acid, thereby increasing the electrophilicity of the carbonyl carbon atom. The enolate then nucleophilically attacks the aldehyde 2 to generate β-hydroxy intermediate A that is subsequently dehydrated to furnish the corresponding Knoevenagel adduct 4. Next, the endocyclic nitrogen of 3-amino-1*H*-1,2,4-triazole 3 undergoes a Michael-type addition onto the activated double bond of 4, leading to intermediate B. The intramolecular nucleophilic attack by the exocyclic amino group of triazole 3 on the more electrophilic and less steric hindered carbonyl carbon atom adjacent to methyl group results in the formation of cyclized intermediate C. Finally, intermediate C undergoes removal of water to furnish 4,7-dihydro[1,2,4]triazolo[1,5-*a*]pyrimidine 5 as the final product.

### 2.2 Biological studies

#### 2.2.1. Molecular docking studies

Molecular docking is a computational technique to study the interaction of ligands with biomolecular targets by predicting the binding site, orientation and binding energy. All novel synthesized [1,2,4]triazolo[1,5-*a*]pyrimidine derivatives (5a–u), and phenylbutazone (PBZ) and ibuprofen (IBP) as site-I and site-II markers, respectively, were studied by *in silico* molecular docking studies with BSA (PDB ID: 4f5s).

**Fig. 3:**
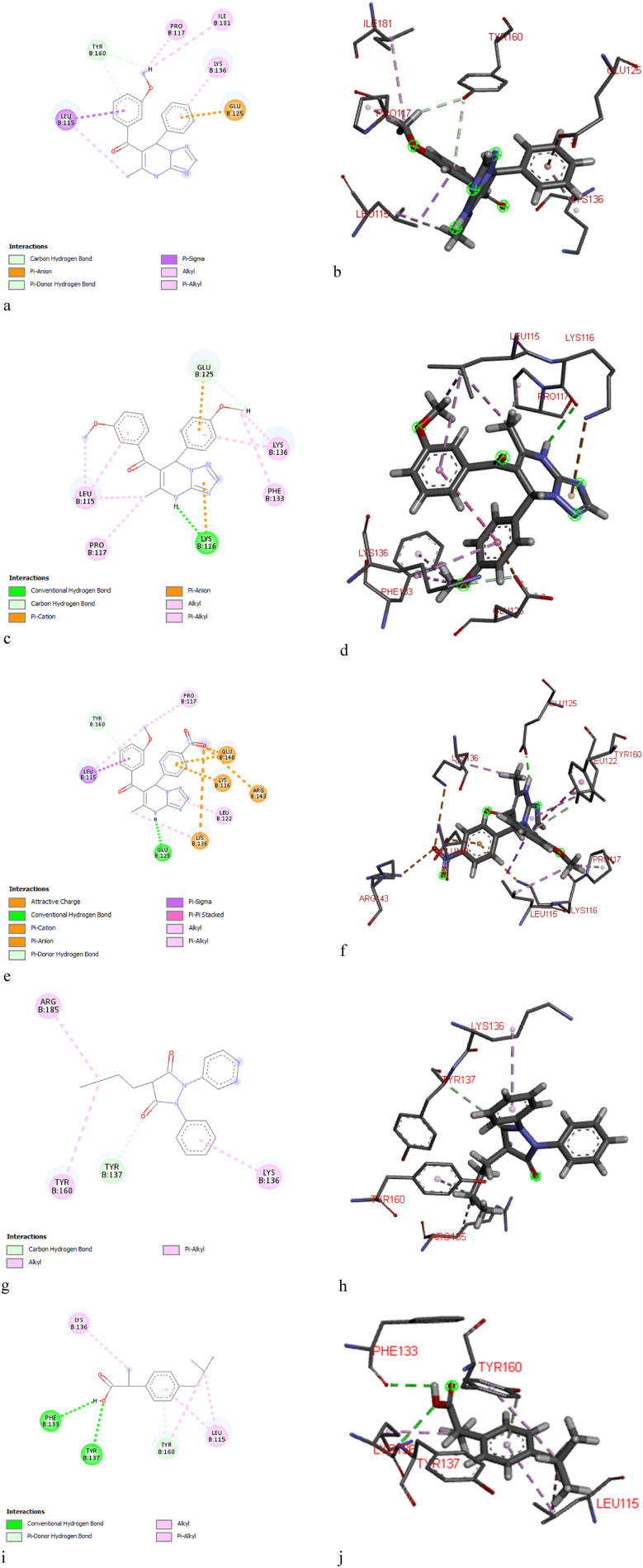
Types of interactions formed between the ligand and the BSA protein: (a and b) 5f, (c and d) 5m and (e and f) 5t, (g and h) phenylbutazone (PBZ) and (i and j) ibuprofen (IBP).

The molecular docking simulation study of [1,2,4]triazolo[1,5-*a*]pyrimidine derivatives (5a–u) with BSA revealed the binding energy scores ranging from −6.0 to −7.9 kcal mol^−1^, indicating moderate to strong binding interactions with the protein (Table 4). Among these, compounds 5c, 5d, and 5f, each bearing an unsubstituted phenyl ring at the 7-position of the fused heterocyclic system, exhibited the strongest affinity interaction with a binding score of −7.9 kcal mol^−1^, compared with ibuprofen as reference, −6.6 kcal mol^−1^. Specifically, compound 5c, with a 4-chlorophenyl substituent, and compound 5d, with a 4-bromophenyl group, demonstrated strong interactions with residues such as Glu125, Lys136, Leu115, Leu122, Pro117, and Phe133, through a combination of hydrogen bonding, alkyl or π-alkyl, π-anion, and hydrophobic interactions (the 2D and 3D plots are reported in Table S1). Compound 5f, containing a 3-methoxyphenyl moiety, showed an equivalent binding score of −7.9 kcal mol^−1^ and formed a hydrogen bond with Tyr160. Moreover, 5f interacted with key residues including Lys136, Ile181, Pro117, Leu115 (alkyl or π-alkyl) and Glu125 (π-anion), as shown in the 2D and 3D plots (Fig. 3).

**Table 4:** Receptor-ligand interactions between the binding pocket of BSA (PDB ID: 4f5s) and [1,2,4]triazolo[1,5-*a*]pyrimidines

Compounds	Binding energy (kcal mol^−1^)	Interacted residues
5a	−7.4	Glu125^a^, Lys136^b^, Leu122^b^^,^^c^, Pro117^b^, Leu115^b^, Lys116^d^
5b	−7.6	Lys116^a^, Lys136^b^, Leu115^b^, Glu125^e^
5c	−7.9	Glu125^a^, Lys136^b^, Pro117^b^, Leu115^b^, Pro113^b^, Glu140^e^, Lys116^f^, Tyr160^g^, Phe133^h^
5d	−7.9	Lys136^b^, Leu115^b^, Pro117^b^, Leu122^b^^,^^c^, Glu125^e^, Lys132^h^
5e	−7.4	Lys136^b^, Leu115^b^, Pro117^b^, Tyr160^g^^,^^h^, Glu125^h^, Thr121^h^, Phe133^i^
5f	−7.9	Lys136^b^, Ile181^b^, Pro117^b^, Leu115^b^^,^^c^, Glu125^e^, Tyr160^g^^,^^h^
5g	−7.1	Lys116^a^, Lys136^b^, Pro117^b^, Leu115^b^, Leu122^b^, Glu125^e^, Thr121^h^
5h	−7.4	Glu125^a^, Pro113^b^, Lys136^b^, Phe133^b^, Glu140^b^^,^^e^^,^^h^, Leu115^c^, Tyr160^g^
5i	−7.3	Glu125^a^, Pro117^b^, Leu122^b^, Pro113^b^, Leu115^c^, Lys116^f^, Tyr160^g^, Glu140^e^^,^^h^
5j	−6.4	Asp118^a^, Lys136^b^, Leu122^b^, Glu140^e^
5k	−6.5	Lys136^b^, Pro117^b^, Leu122^b^, Leu115^b^^,^^c^, Glu125^h^, Phe133^h^^,^^i^
5l	−6.4	Asp118^a^, Lys136^b^, Leu115^b^, Pro113^b^, Leu122^b^, Glu140^e^
5m	−7.0	Lys116^a^^,^^f^, Lys136^b^, Phe133^b^, Pro117^b^, Leu115^b^, Glu125^e^^,^^h^
5n	−6.0	Asp118^a^, Lys136^b^^,^^h^, Leu122^b^, Tyr160^h^
5o	−6.4	Lys136^b^, Leu115^c^, Glu125^e^, Glu140^e^, Lys116^f^
5p	−7.5	Tyr160^a^, Lys116^a^, Lys136^b^, Leu122^b^, Leu115^c^, Glu125^e^, Glu140^e^, Phe133^i^^,^^j^
5q	−6.6	Tyr160^a^, Lys116^a^^,^^f^, Lys136^b^, Leu115^b^^,^^c^, Pro113^b^^,^^h^, Glu125^e^, Glu140^e^, Phe133^h^
5r	−7.3	Glu125^a^, Pro117^b^, Leu115^b^^,^^c^, Lys136^b^^,^^k^, Glu140^e^, Tyr160^g^, Arg143^k^, Arg144^k^
5s	−6.2	Pro113^b^, Leu115^c^, Glu140^e^, Lys116^f^, Thr121^g^^,^^h^, Asp118^h^, Tyr137^g^
5t	−7.4	Glu125^a^, Leu115^b^^,^^c^, Leu122^b^, Pro117^b^, Lys136^b^^,^^k^, Glu140^e^, Lys116^f^, Tyr160^g^, Arg143^k^
5u	−6.5	Glu125^a^, Leu115^b^, Lys136^b^, Leu122^b^, Phe133^b^, Glu140^e^^,^^k^, Lys116^f^, Tyr160^g^, Arg143^k^, Arg144^k^
PBZ	−8.1	Lys136^b^, Tyr160^b^, Arg185^b^, Tyr137^h^
IBP	−6.6	Phe133^a^, Tyr137^a^, Lys136^b^, Leu115^b^, Tyr160^g^

a Hydrogen bonding.

b Alkyl or π-alkyl.

c π-sigma.

d Amide π-stacking.

e π-Anion.

f π-Cation.

g π-Donor hydrogen bond.

h Carbon hydrogen bond.

i π–π stacked.

j Halogen.

k Attractive charge.

A comparative analysis of compounds 5c, 5d, and 5f with compounds bearing a 4-methoxyphenyl group at the 7-position (5j, 5k and 5m) and those substituted with a 4-nitrophenyl group (5q, 5r and 5t) revealed that compound 5m (bearing a 3-methoxyphenyl group) and compound 5t (also bearing a 3-methoxyphenyl group) exhibited the most favorable docking scores within their respective subsets, with binding energies of −7.0 kcal mol^−1^ and −7.4 kcal mol^−1^, respectively (Table 4). Regarding 5m, the pyrimidine-NH demonstrated a hydrogen bond with Lys116, while the triazole core showed a π-cation interaction with Lys116. Alkyl or π-alkyl interaction with residues Lys136, Phe133, Pro117, Leu115 and π-anion and carbon–hydrogen bond with Glu125 also contribute to its enhanced binding affinity. In compound 5t, the pyrimidine-NH showed H-bond with amino acid Glu125. The methyl group and triazole core showed alkyl and π-alkyl interactions with Lys136 and Leu122, respectively. Fig. 3a–j display the lowest energy 2D and 3D poses of the interaction of ligands 5f, 5m, 5t, PBZ and IBP with BSA. Based on the docking results, compounds 5f, 5m, and 5t were selected for further *in vitro* investigation using spectroscopic techniques to evaluate their binding affinity and conformational interactions with BSA. Their superior binding energies and favorable interaction profiles with crucial BSA residues underscore their potential as effective BSA-binding agents among the studied triazolopyrimidine derivatives.

The synthesized [1,2,4]triazolo[1,5-*a*]pyrimidine derivatives (5a–u) possess a stereogenic center, and were obtained as racemic mixtures; therefore, the initial molecular docking studies with BSA (PDB ID: 4f5s) were performed using the racemic forms of the ligands. In order to evaluate the influence of stereochemistry on protein–ligand interactions, additional docking studies were carried out for both *R*- and *S*-enantiomers of selected representative compounds (5a, 5f, 5m, and 5t) (the 2D and 3D plots are reported in Table S2).

The docking results demonstrated a clear enantioselective preference, with the *R*-enantiomers exhibiting more favorable binding energies toward BSA than their corresponding *S*-enantiomers. This difference in binding affinity can be attributed to improved geometric complementarity and more effective interactions of the *R*-enantiomers within the BSA binding pocket. Notably, the racemic mixtures of these compounds displayed higher binding energies than either individual enantiomer, suggesting a cumulative or averaged interaction profile when both enantiomers are considered together.

These findings indicate that stereochemistry plays a significant role in modulating the binding affinity of the synthesized compounds toward BSA. However, the racemic form, consistent with the experimentally studied samples, provides an appropriate and reliable representation of protein–ligand interactions in the present docking study.

#### 2.2.2. Binding studies of [1,2,4]triazolo[1,5-*a*]pyrimidine with BSA

**Fig. 4:**
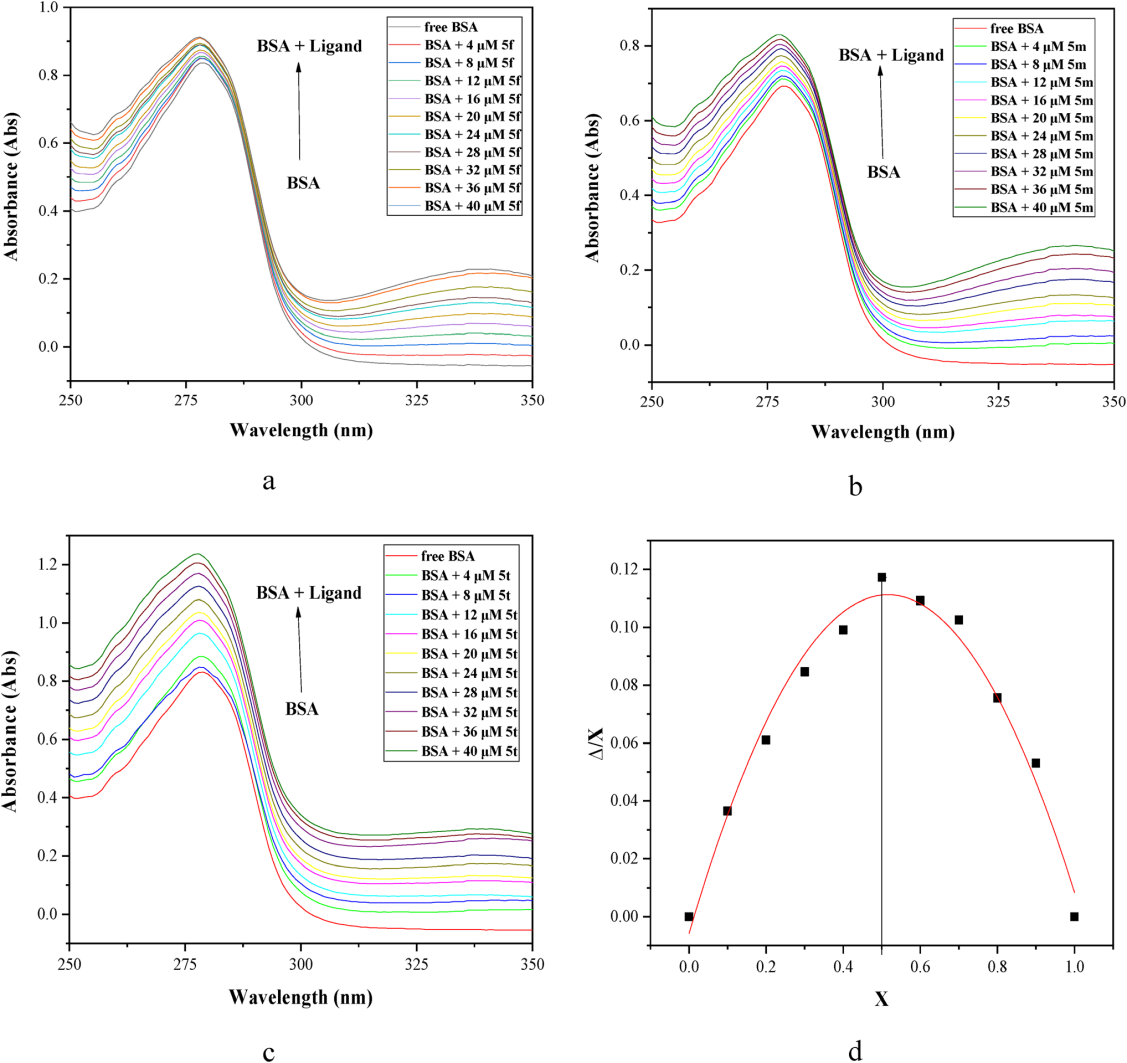
UV-visible spectra of BSA in the absence and presence of different concentrations (0–40 µM) of ligands (a) 5f, (b) 5m and (c) 5t at a constant BSA concentration of 15 µM under a physiological pH 7.2 of PBS at room temperature. (d) Job's plot for the BSA-5t complex system.

UV-vis spectrophotometric titration serves as an effective analytical method for investigating conformational changes in proteins and assessing their interactions with drug molecules.^37^ This technique can be employed to distinguish between dynamic and static quenching mechanisms by monitoring changes in the protein's absorption spectrum. Significant spectral shifts suggest static quenching *via* ground–state complex formation. During the dynamic quenching, no significant alteration in the absorption profile of protein is observed. Interaction between BSA and ligands perturbs the protein's electronic environment, leading to shifts in wavelength or changes in absorption intensity in the UV-vis spectrum.^38^ Among the synthesized compounds, 5f having no substituent on the phenyl ring at the 7-position, 5m having electron-donating 4-OMe and 5t having electron withdrawing 4-NO_2_ on the phenyl ring were selected for UV-visible spectral studies with BSA. The UV spectra of BSA solutions in the absence and presence of 5f, 5m, and 5t at different concentrations ranging from 0 to 40 µM were recorded in the wavelength range of 250–350 nm at room temperature. The absorption spectrum of BSA exhibits a peak at 278 nm due to π–π* transitions of aromatic amino acids, primarily tryptophan (Trp), tyrosine (Tyr) and phenylalanine (Phe) residues.^39^ The addition of increasing concentrations of 5f, 5m and 5t to the BSA solution results in noteworthy hyperchromicity at 278 nm, with a slight blue shift (Δ*λ* ∼ 1 nm), indicating static interaction *via* complex formation between [1,2,4]triazolo[1,5-*a*]pyrimidines 5 and amino acid residues of BSA (Fig. 4a–c).

The stoichiometry of the BSA-ligand complex was determined using Job's method of continuous variation. Ten different solutions were prepared with different volumes of BSA and 5t fixed at a constant concentration of 15 µM. In Job's plot, the absorbance at *λ*_max_ (278 nm) was measured, and absorbance *versus* mole fraction of ligand 5t was plotted (Fig. 4d). The maximum change in absorbance was observed at 0.5 on the axis at a mole fraction corresponding to a 1 : 1 binding ratio, indicating the formation of a 1 : 1 BSA-ligand complex 5t.^40^

#### 2.2.3. Fluorescence quenching studies

Fluorescence spectroscopy has emerged as a powerful and sensitive technique for elucidating the interaction mechanisms and binding affinities between ligands and biomolecules such as BSA. Intrinsic fluorescence of BSA is primarily due to the presence of aromatic amino acid residues, notably Trp, Tyr, and Phe. Among these, tryptophan exhibits the strongest fluorescence due to its high quantum yield and pronounced sensitivity to environmental changes. Fluorescence emission from Trp serves as an effective probe for monitoring structural alterations in the protein, including conformational changes, subunit interactions, denaturation, and ligand binding. Thus, changes in fluorescence intensity or emission wavelength reflect interactions near Trp residues, making fluorescence spectroscopy a valuable tool for site-specific and mechanistic analysis of protein–ligand interactions.

**Fig. 5:**
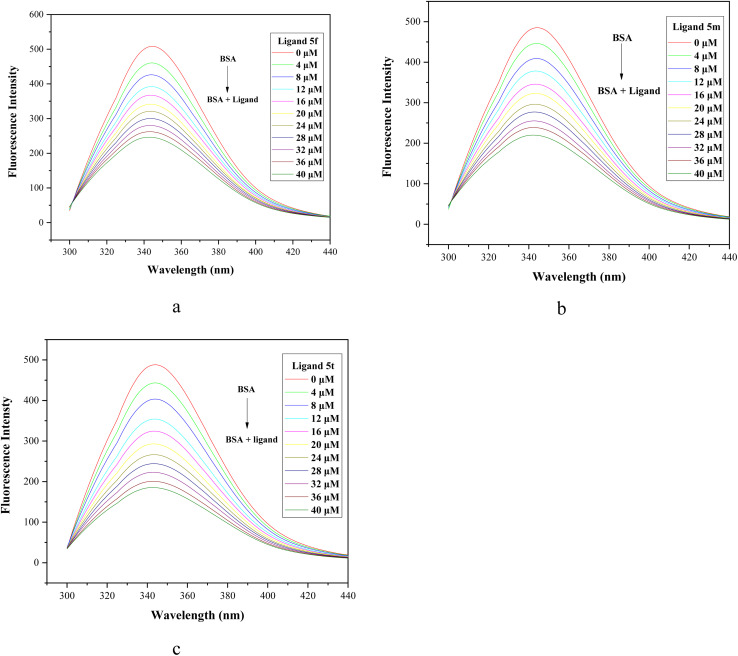
Emission spectra of BSA (15 µM) in the presence of increasing concentrations (0–40 µM) of compounds (a) 5f, (b) 5m, and (c) 5t.

Fluorescence spectroscopy experiments were conducted with BSA (15 × 10^−6^ M) and varying concentrations of compounds 5f, 5m, and 5t (0–40 µM). The emission spectra were monitored in the 300–450 nm range after excitation at 280 nm. The results revealed that the fluorescence intensity of BSA around 342 nm decreased progressively with the gradual increase in ligand concentration (Fig. 5a–c) and exhibited a slight blue shift (*e.g.* 4 nm in the case of 5f). The addition of ligand causes a quenching in the fluorescence spectrum of BSA, primarily arising from the conformational and dynamic changes in the protein structure.

The extent of quenching depends upon the extent of ligand-BSA interaction and can be determined by calculating the quenching constant *K*_q_ using the Stern–Volmer equation^41^ (eqn 1):1
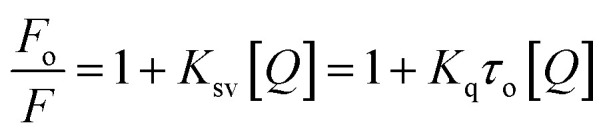
where [*Q*] is the ligand (quencher) concentration, *F*_o_ and *F* are the fluorescence intensities of BSA in the absence and presence of ligands (5f, 5m and 5t) at different concentrations (0–40 µM), *K*_SV_ and *K*_q_ are the Stern–Volmer constant and quenching constant, respectively, and *τ*_o_ denotes the average fluorescence lifetime of BSA. Quenching constant *K*_q_ was calculated by employing the average lifetime *τ*_o_ = 10^−8^ s.

**Fig. 6:**
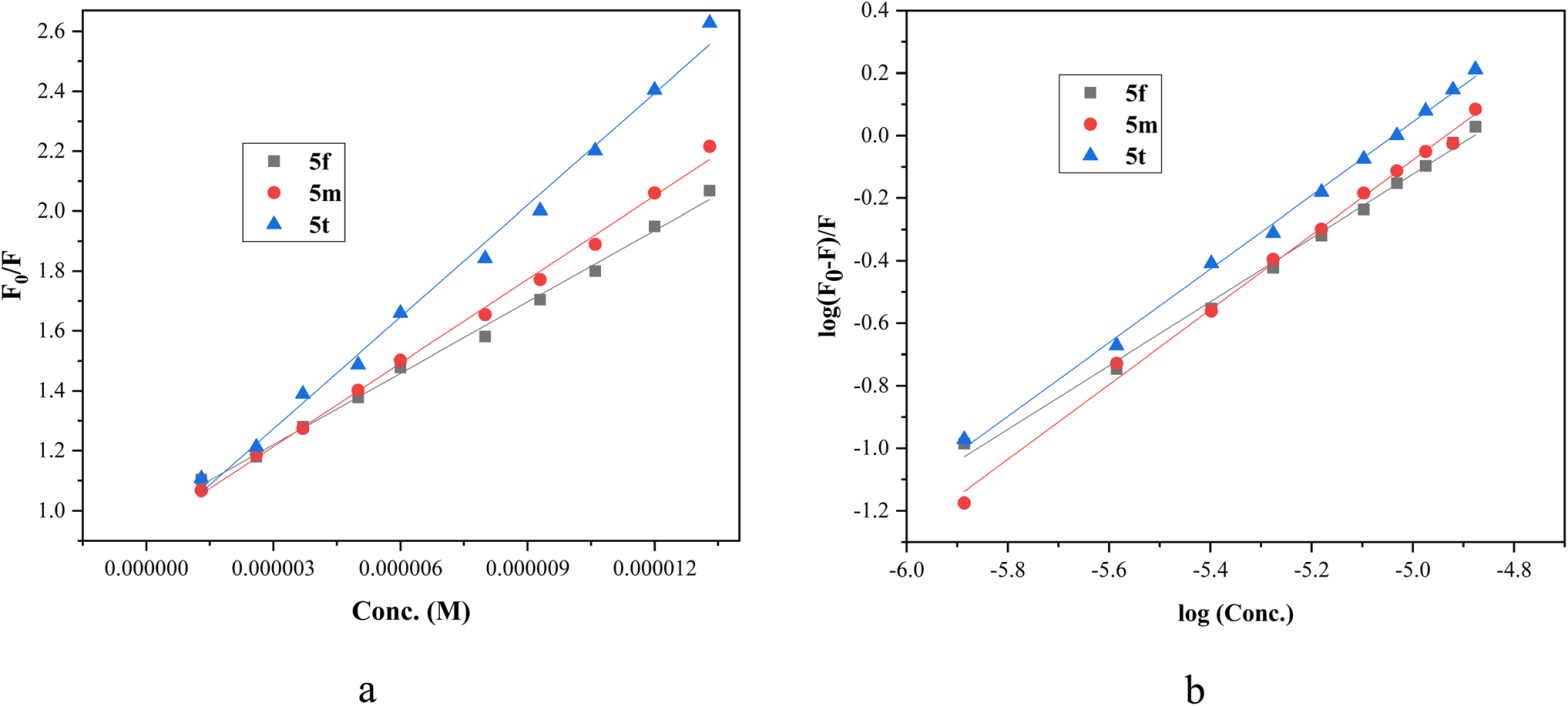
(a) Stern–Volmer plot of BSA quenching by compounds 5f, 5m and 5t. (b) Double logarithmic plot employed to determine the binding parameters for compounds 5f, 5m and 5t.

The plot of *F*_o_/*F versus* 1/[*Q*] of ligand quencher with BSA revealed linear relationship, as shown in Fig. 6a. The *K*_SV_ and *K*_q_ values of BSA triggered by ligands at room temperature were determined by calculating the slope of curve, and are given in Table 5. The values of quenching constant *K*_q_ at 298 K for ligands 5f, 5m and 5t are 7.94 × 10^12^, 9.31 × 10^12^ and 12.45 × 10^12^, respectively. The *K*_q_ values (∼10^12^ M^−1^ s^−1^) of ligands are greater than maximum scatter collision quenching constant^42^(2.0 × 10^10^ M^−1^ s^−1^), indicating the static quenching mechanism involving ground–state complex formation with BSA. According to the *K*_q_ values, the ability of the ligand to quench the emission intensity of BSA follows the order of 5t > 5m > 5f, suggesting that the ligand structure significantly influences the binding affinity.

**Table 5:** Stern–Volmer constant, binding constant, quenching constant and number of binding sites for the interaction of BSA with compounds 5f, 5m and 5t

Compd	*K*_SV_ × 10^4^ (M^−1^)	*K*_q_ × 10^12^ (M^−1^ s^−1^)	log *K*_b_	*K*_b_ (M^−1^)	*n*	Δ*G*° (kcal mol^−1^)
5f	7.94 ± 18	7.94 ± 18	4.97 ± 0.13	9.5 × 10^4^	1.0 ± 0.02	−6.83
5m	9.31 ± 19	9.31 ± 19	5.90 ± 0.15	8.0 × 10^5^	1.1 ± 0.02	−11.02
5t	12.45 ± 36	12.45 ± 36	5.93 ± 0.11	8.5 × 10^5^	1.1 ± 0.02	−11.72

##### 2.2.3.1. Identification of the binding constant and the number of binding sites

The binding constant *K*_b_ and the number of binding sites (*n*) were calculated using the Scatchard equation^43^ (eqn 2) as follows:2
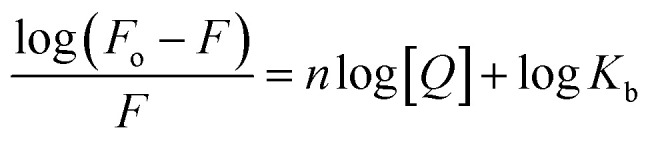


From the plot of log(*F*_o_ − *F*)/*F* against log[*Q*] (Fig. 6b), the binding constant *K*_b_ and the number of binding sites *n* were obtained from the intercept and slope, respectively. The calculated results are given in Table 5. The observed *n* value close to 1 supports the presence of a single binding site on BSA for triazolopyrimidines 5. The binding constant *K*_b_ for ligands 5f, 5m, and 5t lies within the favorable range of 10^4^–10^6^ M^−1^, indicating moderate and reversible interactions with BSA. Such binding strength is considered optimal for carrier proteins, as it allows efficient ligand transport while enabling controlled release at the target site. The results indicate that the compounds bind to BSA in the order of 5t > 5m > 5f. Furthermore, the negative value of standard Gibbs free energy change (Δ*G*°) from eqn 3 suggests the spontaneity of the binding process that resulted in the BSA-triazolopyrimidine complex:

3 Δ*G*° = −*RT* ln *K*_b_

#### 2.2.4. BSA site marker fluorescence quenching studies

Competitive displacement assays using site-specific markers of BSA are widely employed to elucidate the binding site and affinity of test ligands. The primary drug-binding sites of BSA are Sudlow's site I and site II, located within the hydrophobic cavities of subdomains IIA and IIIA, respectively. Site markers are small molecules that bind selectively to specific sites on the protein structure, and are commonly used to investigate the binding interactions of various ligands with the protein. Site I markers include phenylbutazone, warfarin, dansylamide, and iodipamide, whereas ibuprofen, flufenamic acid, dansylglycine and diazepam are commonly used site II markers.^44^ To identify the binding site of ligand 5t on BSA, a displacement assay was conducted using phenylbutazone (PBZ) and ibuprofen (IBP) as site-specific probes for Sudlow's sites I and II, respectively. BSA-binding constants of the ligand were determined in the presence of PBZ and IBP using fluorescence emission spectroscopy and compared to the values obtained in the absence of site markers (Table 6).

**Table 6:** Binding constants for the BSA-5t complex in the presence of site markers at 298 K

System	log *K*_b_	*K*_b_ (M^−1^)
BSA + 5t	5.9316 ± 0.114	8.54 × 10^5^
BSA + PBZ + 5t	3.94411 ± 0.209	0.87 × 10^4^
BSA + IBP + 5t	4.75972 ± 0.180	5.75 × 10^4^

**Fig. 7:**
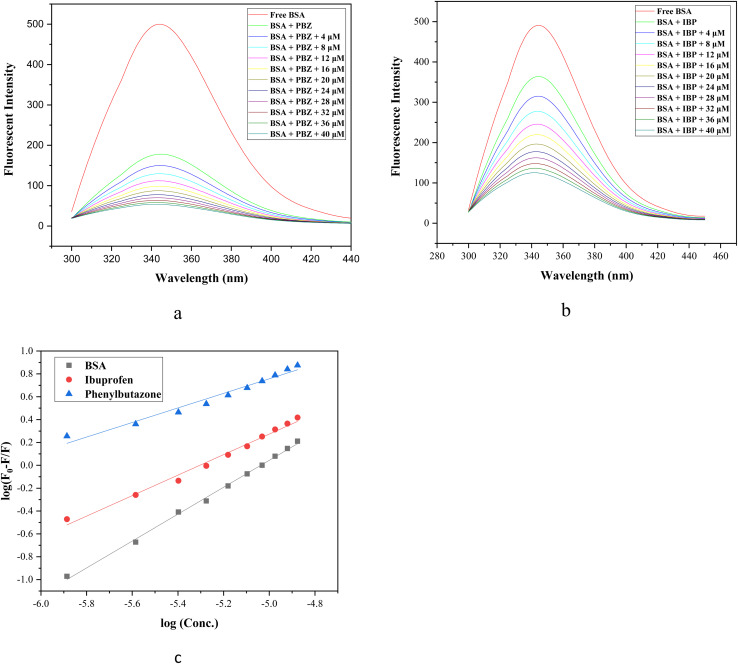
Fluorescence spectra of (a) BSA-PBZ and (b) BSA-IBP complex in the absence and presence of increasing concentrations of 5t (0–40 µM) at 298 K. (c) Double logarithmic plot employed to determine the binding constants for compound 5t in the absence (DMSO) and presence of site markers, ibuprofen and phenylbutazone.

In this experiment, the fluorescence spectra of BSA and site marker-BSA (BSA-PBZ) (BSA-IBP) complexes (1 : 1) were recorded in the absence and presence of incremental addition of ligand 5t (0–40 µM) (Fig. 7a and b). The fluorescence intensity of the BSA solution decreases upon the addition of the site marker, indicating that the site marker has attached to the BSA molecule. Sequential addition of 5t to the site marker-BSA solution resulted in a gradual decrease in the fluorescence emission intensity, and the data thus obtained were utilized to plot a graph of log(*F*_o_ − *F*)/*F* against log[*Q*] for complex 5t in the absence and presence of site markers, as shown in Fig. 7c. The binding constant *K*_b_ for phenylbutazone and ibuprofen was found to be 0.87 × 10^4^ and 5.75 × 10^4^, respectively (Table 6). A decrease in the *K*_b_ and log *K*_b_ values of the compound 5t with BSA in the presence of phenylbutazone and ibuprofen, compared to the *K*_b_ value in the absence of site marker, indicates that the complex and the site marker compete for the same binding site.^45^ As ligand 5t contains a single binding site, it can interact with either site I or site II on the BSA molecule, but not both simultaneously. Nevertheless, a significant decrease in the *K*_b_ value in the presence of phenylbutazone compared to ibuprofen was calculated, suggesting the selective binding of compound 5t to Sudlow's site I of BSA.

## 3. Conclusions

In summary, we have developed an efficient protocol for the regioselective synthesis of a series of 6-aroyl-7-aryl-5-methyl-[1,2,4]triazolo[1,5-*a*]pyrimidines by three-component condensation of 3-amino-1*H*-1,2,4-triazole with aromatic aldehydes and β-diketones. Out of the possible regioisomers, the exact one was identified using multinuclear 2D-NMR [^1^H–^13^C] HMBC; [^1^H–^13^C] HSQC and [^1^H–^15^N] HMBC spectroscopic techniques. Molecular docking studies were conducted to assess the interactions of the synthesized compounds with BSA amino acid residues, which indicated that the binding of compounds to BSA is primarily mediated by hydrogen bonding and hydrophobic interactions. Selected compounds (5f, 5m and 5t) were evaluated for their binding interaction with BSA using UV-absorption spectroscopy, fluorescence and competitive displacement assays. Fluorescence quenching studies demonstrated that the compounds interact with BSA *via* a static quenching mechanism, indicating the formation of a ground-state complex. Among the tested compounds, 5t exhibited the highest binding affinity and most effectively quenched the intrinsic fluorescence of BSA. Furthermore, site-marker competitive binding assay indicated that ligand 5t preferentially binds at Sudlow's site I (in subdomain IIA) of BSA.

## 4. Experimental

### 4.1. General

All the chemicals and solvents used in the present study were purchased from commercial suppliers of Hi-media and Avera, India, and used without further purification. To monitor the progress of the reaction and purity of the products, TLC experiments were conducted on 0.2 mm Merck precoated silica gel 60 F254-coated aluminum plates. A mixture of ethyl acetate and petroleum ether was used as the mobile phase and spots were visualized under UV light at 254 nm. The melting points were determined using an electrical digital Melting Point Apparatus (MEPA) in an open capillary tube, which were uncorrected. IR spectra were recorded using a Buck Scientific IR M–500 instrument with KBr pellets (*υ*_max_ in cm^−1^). ^1^H and ^13^C NMR spectra were recorded using a Bruker instrument at frequencies of 400 and 100 MHz, respectively, with DMSO-*d*_6_ as a solvent and tetramethylsilane (TMS) as an internal standard (the chemical shift in *δ* scale and coupling constants (*J*) were expressed in parts per million (ppm) and hertz, respectively). High-resolution mass spectra (HRMS) were recorded in the ESI^+^ mode at CIL GJU, Hisar. Furthermore, 2D correlation spectroscopy, (^1^H–^13^C) gs-HSQC and (^1^H–^13^C) gs-HMBC of the samples were carried out at Kurukshetra University, Kurukshetra. The UV-vis spectra were recorded using a UV-vis spectrophotometer 117 (Systronic, India) with a 1 cm-path-length cell. Fluorescence spectra were recorded using a Shimadzu-5301pc spectro-fluorophotometer (Kyoto, Japan).

### 4.2. General procedure for the synthesis of 6-aroyl-7-aryl-5-methyl-4,7-dihydro- [1,2,4]triazolo[1,5-*a*]pyrimidines (5a–u)

A mixture of acetic acid-piperidine (1 : 1) was taken in a 100 mL round-bottomed flask and cooled in an ice bath. The appropriate diketone 1 (1.0 mmol) and aldehyde 2 (1.0 mmol) were added to the flask and stirred for half an hour under ice-cold conditions and then stirred at room temperature overnight. 3-Amino-1*H*-1,2,4-triazole 3 (84 mg, 1.0 mmol) and 10 mL acetic acid were added, and the mixture was heated under reflux for 6–7 h. After the completion of the reaction (TLC monitoring), the reaction mixture was allowed to cool to room temperature. The reaction mixture was neutralized with a saturated sodium bicarbonate solution (15 mL) and extracted with EtOAc (3 × 15 mL). The combined organic layers were then dried over anhydrous MgSO_4_ and evaporated *in vacuo*. The product obtained was recrystallized from ethanol to give the pure 1,2,4]triazolo[1,5-*a*]pyrimidines 5a–u.

#### 4.2.1. (5-Methyl-7-phenyl-4,7-dihydro-[1,2,4]triazolo[1,5-*a*]pyrimidin-6-yl)(phenyl)methanone (5a)

White solid; m.p. 250 °C; yield 80%; IR (KBr) (*ν*_max_/cm^−1^): 1628 (C=O), 3466 (NH); ^1^H NMR (400 MHz, DMSO-*d*_6_) *δ*: 10.73 (s, 1H, NH); 7.68 (s, 1H, triazole 3-H); 7.54–7.48 (m, 3H, 3″, 4″, 5″-H); 7.44–7.41 (t, 2H, *J* = 7.4 Hz, 2″, 6″-H); 7.29–7.26 (t, 2H, *J* = 7.4 Hz, 2′, 6′-H); 7.23–7.16 (m, 3H, 3′, 4′, 5′-H); 6.42 (s, 1H, C–H); 1.82 (s, 3H, CH_3_); ^13^C NMR (100 MHz, DMSO-*d*_6_) *δ*: 194.95, 150.60, 143.25, 141.74, 141.14, 132.33, 129.17, 128.98, 128.50, 128.40, 128.35, 127.60, 107.73, 61.24, and 19.56; MS (EI) *m*/*z*: calcd for C_19_H_16_N_4_O: 316.1324; found: 317.1328 [M + 1]^+^, elemental analysis: calcd for C_19_H_16_N_4_O: C, 72.13%; H, 5.10%; and N, 17.71%. Found: C, 72.21%; H, 5.28%; and N, 17.59%.

#### 4.2.2. (4″-Fluorophenyl)(5-methyl-7-phenyl-4,7-dihydro-[1,2,4]triazolo[1,5-*a*]pyrimidin-6-yl)methanone (5b)

White solid; m.p. 268.5 °C; yield 82%; IR (KBr) (*ν*_max_/cm^−1^): 1628 (C=O), 3428 (NH); ^1^H NMR (400 MHz, DMSO-*d*_6_) *δ*: 10.74 (s, 1H, NH); 7.68 (s, 1H, triazole 3-H); 7.60–7.57 (dt, 2H, *J*_o_ = 7.2 Hz, *J*_(m)HF_ = 4.6 Hz, 2″, 6″-H); 7.30–7.20 (m, 5H, 2′, 3′, 4′, 5′, 6′-H); 7.20–7.17 (dd, 2H, *J*_o_ = 7.6 Hz, *J*_(m)HF_ = 4.8 Hz, 3″, 5″-H); 6.41 (s, 1H, C–H); 1.84 (s, 3H, CH_3_); ^13^C NMR (100 MHz, DMSO-*d*_6_) *δ*: 192.99, 165.26–162.77 (d, ^1^*J*_C–F_ = 249 Hz, Ph-4″-C), 149.95, 147.14, 142.54, 141.04, 136.91, 136.88, 130.74–130.65 (d, ^3^*J*_C–F_ = 9 Hz, Ph-2″, 6″-C), 128.37, 127.90, 127.00, and 115.70–115.49 (d, ^2^*J*_C–F_ = 21 Hz, Ph-3″, 5″-C), 106.89, 60.67, 18.97; MS (EI) *m*/*z*: calcd for C_19_H_15_FN_4_O: 334.1230; found: 335.1239 [M + 1]^+^, elemental analysis: calcd for C_19_H_15_FN_4_O: C, 68.25%; H, 4.52%; and N, 16.76%. Found: C, 68.13%; H, 4.38%; N, and 16.62%.

#### 4.2.3. (4″-Chlorophenyl)(5-methyl-7-phenyl-4,7-dihydro-[1,2,4]triazolo[1,5-*a*]pyrimidin-6-yl)methanone (5c)

White solid; m.p. 267.5 °C; yield 80%; IR (KBr) (*ν*_max_/cm^−1^): 1628 (C=O), 3428 (NH); ^1^H NMR (400 MHz, DMSO-*d*_6_) *δ*: 10.80 (s, 1H, NH); 7.69 (s, 1H, triazole 3-H); 7.53–7.51 (d, 2H, *J* = 8.8 Hz, 2″, 6″-H); 7.50–7.48 (d, 2H, *J* = 8.8 Hz, 3″, 5″-H); 7.29–7.26 (t, 2H, *J* = 7.2 Hz, 2′, 6′-H); 7.23–7.21 (m, 1H, 4′-H); 7.19–7.17 (d, 2H, *J* = 7.6 Hz, 3′, 5′-H); 6.40 (s, 1H, C–H); 1.86 (s, 3H, CH_3_); ^13^C NMR (100 MHz, DMSO-*d*_6_) *δ*: 193.70, 150.60, 147.68, 143.91, 141.68, 139.79, 137.08, 130.37, 129.31, 129.00, 128.53, 127.62, 107.45, 61.18, and 19.75; MS (EI) *m*/*z*: calcd for C_19_H_15_ClN_4_O: 350.0934; found: 351.0981 [M + 1]^+^, 352.1015 [M + 2]^+^, and elemental analysis: calcd for C_19_H_15_ClN_4_O: C, 65.05%; H, 4.31%; N, 15.97%. Found: C, 65.18%; H, 4.43%; and N, 15.86%.

#### 4.2.4. (4″-Bromophenyl)(5-methyl-7-phenyl-4,7-dihydro-[1,2,4]triazolo[1,5-*a*]pyrimidin-6-yl)methanone (5d)

White solid; m.p. 274.5 °C; yield 81%; IR (KBr) (*ν*_max_/cm^−1^): 1628 (C=O); ^1^H NMR (400 MHz, DMSO-*d*_6_) *δ*: 10.81 (s, 1H, NH); 7.67 (s, 1H, triazole 3-H); 7.64–7.62 (d, 2H, *J* = 8.4 Hz, 2″, 6″-H); 7.45–7.43 (d, 2H, *J* = 8.4 Hz, 3″, 5″-H); 7.29–7.26 (t, 2H, *J* = 7.2 Hz, 2′, 6′-H); 7.23–7.21 (m, 1H, 4′-H); 7.19–7.17 (t, 2H, *J* = 7.2 Hz, 3′, 5′-H); 6.40 (s, 1H, C–H); 1.86 (s, 3H, CH_3_); ^13^C NMR (100 MHz, DMSO-*d*_6_) *δ*: 193.80, 150.61, 147.69, 143.99, 141.70, 140.16, 132.23, 130.51, 128.99, 128.52, 127.61, 126.06, 107.43, 61.16, and 19.78; MS (EI) *m*/*z*: calcd for C_19_H_15_BrN_4_O: 394.0429; found: 395.0438 [M + 1]^+^, and elemental analysis: calcd for C_19_H_15_BrN_4_O: C, 57.74%; H, 3.83%; N, 14.17%. Found: C, 57.67%; H, 3.695; and N, 14.39%.

#### 4.2.5. (4″-Methoxyphenyl)(5-methyl-7-phenyl-4,7-dihydro-[1,2,4]triazolo[1,5-*a*]pyrimidin-6-yl)methanone (5e)

White solid; m.p. 256.5 °C; yield 86%; IR (KBr) (*ν*_max_/cm^−1^): 1628 (C=O); ^1^H NMR (400 MHz, DMSO-*d*_6_) *δ*: 10.54 (s, 1H, NH); 7.67 (s, 1H, triazole 3-H); 7.54–7.52 (d, 2H, *J* = 8.4 Hz, 2″, 6″-H); 7.28–7.24 (t, 2H, *J* = 7.6 Hz, 2′, 6′-H); 7.22–7.18 (m, 1H, 4′-H); 7.16–7.14 (d, 2H, *J* = 7.6 Hz, 3′, 5′-H); 6.96–6.94 (d, 2H, *J* = 8.8 Hz, 3″, 5″-H); 6.40 (s, 1H, C–H); 3.80 (s, 3H, 4″-OCH_3_); 1.82 (s, 3H, CH_3_); ^13^C NMR (100 MHz, DMSO-*d*_6_) *δ*: 193.82, 163.00, 150.52, 148.11, 141.58, 140.48, 132.96, 131.11, 128.98, 128.48, 127.54, 114.44, 107.66, 61.56, 55.96, and 19.21; MS (EI) *m*/*z*: calcd for C_20_H_18_N_4_O_2_: 346.1430; found: 347.1414 [M + 1]^+^, and elemental analysis: calcd for C_20_H_18_N_4_O_2_: C, 69.35%; H, 5.24%; and N, 16.17%. Found: C, 69.21%; H, 5.43%; and N, 16.26%.

#### 4.2.6. (3″-Methoxyphenyl)(5-methyl-7-phenyl-4,7-dihydro-[1,2,4]triazolo[1,5-*a*]pyrimidin-6-yl)methanone (5f)

White solid; m.p. 248 °C; yield 82%; IR (KBr) (*ν*_max_/cm^−1^): 1628 (C=O); ^1^H NMR (400 MHz, DMSO-*d*_6_) *δ*: 10.72 (s, 1H, NH); 7.68 (s, 1H, triazole 3-H); 7.36–7.32 (t, 1H, *J* = 8 Hz, 5″-H); 7.30–7.26 (t, 2H, *J* = 7.6 Hz, 2′, 6′-H); 7.24–7.20 (m, 1H, 4′-H); 7.18–7.16 (d, 2H, *J* = 7.6 Hz, 3′, 5′-H); 7.10–7.07 (dd, 1H, *J* = 8.4 Hz, *J* = 2.8 Hz, 6″-H); 7.06–7.04 (d, 1H, *J* = 8 Hz, 4″-H); 6.94 (br s, 1H, 2″-H); 6.41 (s, 1H, C–H); 3.74 (s, 3H, 3″-OCH_3_); 1.84 (s, 3H, CH_3_); ^13^C NMR (100 MHz, DMSO-*d*_6_) *δ*: 194.69, 159.80, 150.59, 147.81, 143.31, 142.68, 142.65, 141.77, 130.36, 128.99, 128.50, 127.56, 120.71, 118.17, 112.87, 107.80, 61.20, 55.78, and 19.44; MS (EI) *m*/*z*: calcd for C_20_H_18_N_4_O_2_: 346.1430; found: 347.1537 [M + 1]^+^, and elemental analysis: calcd for C_20_H_18_N_4_O_2_: C, 69.35%; H, 5.24%; and N, 16.17%. Found: C, 69.26%; H, 5.37%; and N, 16.48%.

#### 4.2.7. (5-Methyl-7-phenyl-4,7-dihydro-[1,2,4]triazolo[1,5-*a*]pyrimidin-6-yl)(thiophen-2″-yl)methanone (5g)

White solid; m.p. 269.5 °C; yield 79%; IR (KBr) (*ν*_max_/cm^−1^): 1628 (C=O); ^1^H NMR (400 MHz, DMSO-*d*_6_) *δ*: 10.57 (s, 1H, NH); 7.92–7.90 (dd, 1H, *J*_1_ = 4.8 Hz, *J*_2_ = 1.2 Hz, 5″-H); 7.71–7.70 (dd, 1H, *J*_1_ = 4.8 Hz, *J*_2_ = 1.2 Hz, 3″-H); 7.66 (s, 1H, triazole 3-H); 7.28–7.24 (m, 2H, 2′,6′-H); 7.22–7.18 (m, 3H, 3′, 4′, 5′-H); 7.15–7.13 (dd, 1H, *J*_1_ = 4.8 Hz, *J*_2_ = 3.6 Hz, 4″-H); 6.44 (s, 1H, C–H); 1.96 (s, 3H, CH_3_); ^13^C NMR (100 MHz, DMSO-*d*_6_) *δ*: 186.96, 150.51, 147.98, 145.29, 141.24, 140.15, 134.96, 134.09, 128.98, 128.92, 128.61, 127.75, 107.61, 61.61, and 19.19; MS (EI) *m*/*z*: calcd for C_17_H_14_N_4_OS: 322.0888; found: 323.0869 [M + 1]^+^, and elemental analysis: calcd for C_17_H_14_N_4_OS: C, 63.34%; H, 4.38%; and N, 17.38%. Found: C, 63.23%; H, 4.27%; and N, 17.19%.

#### 4.2.8. (7-(4′-Methoxyphenyl)-5-methyl-4,7-dihydro-[1,2,4]triazolo[1,5-*a*]pyrimidin-6-yl) (phenyl)methanone (5h)

White solid; m.p. 220.5 °C; yield 86%; IR (KBr) (*ν*_max_/cm^−1^): 1628 (C=O), 3472 (NH); ^1^H NMR (400 MHz, DMSO-*d*_6_) *δ*: 10.69 (s, 1H, NH); 7.67 (s, 1H, triazole 3-H); 7.55–7.49 (m, 3H, 3″,4″,5″-H); 7.45–7.42 (m, 2H, 2″, 6″-H); 7.13–7.10 (d, 2H, *J* = 8.8 Hz, 2′, 6′-H); 6.83–6.81 (d, 2H, *J* = 8.8 Hz, 3′, 5′-H); 6.38 (s, 1H, C–H); 3.68 (s, 3H, 4′-OCH_3_); 1.83 (s, 3H, CH_3_); ^13^C NMR (100 MHz, DMSO-*d*_6_) *δ*: 195.06, 159.36, 150.46, 147.64, 142.95, 141.07, 133.88, 132.35, 129.18, 128.93, 128.42, 128.37, 114.26, 107.86, 60.69, 55.54, and 19.50; MS (EI) *m*/*z*: calcd for C_20_H_18_N_4_O_2_: 346.1430; found: 347.1439 [M + 1]^+^, and elemental analysis: calcd for C_20_H_18_N_4_O_2_: C, 69.35%; H, 5.24%; and N, 16.17%. Found: C, 69.29%; H, 5.15%; and N, 16.42%.

#### 4.2.9. (4″-Fluorophenyl)(7-(4′-methoxyphenyl)-5-methyl-4,7-dihydro-[1,2,4]triazolo[1,5-*a*]pyrimidin-6-yl)methanone (5i)

White solid; m.p. 223 °C; yield 92%; IR (KBr) (*ν*_max_/cm^−1^): 1628 (C=O); ^1^H NMR (400 MHz, DMSO-*d*_6_) *δ*: 10.68 (s, 1H, NH); 7.66 (s, 1H, triazole 3-H); 7.62–7.58 (dt, 2H, *J* = 7.2 Hz, *J* = 5.6 Hz, 2″, 6″-H); 7.28–7.24 (t, 2H, *J* = 8.8 Hz, 3″, 5″-H); 7.12–7.10 (d, 2H, *J* = 8.4 Hz, 2′, 6′-H); 6.83–6.81 (d, 2H, *J* = 8.8 Hz, 3′, 5′-H); 6.35 (s, 1H, C–H); 3.68 (s, 3H, 4′-OCH_3_); 1.85 (s, 3H, CH_3_); ^13^C NMR (100 MHz, DMSO-*d*_6_) *δ*: 193.73, 165.89–163.40 (d, ^1^*J*_C–F_ = 249 Hz, Ph-4″-C), 159.39, 150.44, 147.60, 142.87, 137.47, 133.78, 131.39–131.29 (d, ^3^*J*_C–F_ = 10 Hz, Ph-2″, 6″-C), 128.95, 116.34–116.12 (d, ^2^*J*_C–F_ = 22 Hz, Ph-3″, 5″-C), 114.28, 107.66, 60.75, 55.55, and 19.52; MS (EI) *m*/*z*: calcd for C_20_H_17_FN_4_O_2_: 364.1336; found: 365.1372 [M + 1]^+^, and elemental analysis: calcd for C_20_H_17_FN_4_O_2_: C, 65.93%; H, 4.70%; and N, 15.38%. Found: C, 65.66%; H, 4.62%; and N, 15.33%.

#### 4.2.10. (4″-Chlorophenyl)(7-(4′-methoxyphenyl)-5-methyl-4,7-dihydro-[1,2,4]triazolo[1,5-*a*]pyrimidin-6-yl)methanone (5j)

White solid; m.p. 219.5 °C; yield 90%; IR (KBr) (*ν*_max_/cm^−1^): 1628 (C=O); ^1^H NMR (400 MHz, DMSO-*d*_6_) *δ*: 10.76 (s, 1H, NH); 7.67 (s, 1H, triazole 3-H); 7.65–7.63 (d, 2H, *J* = 8.4 Hz, 2″, 6″-H); 7.46–7.44 (d, 2H, *J* = 8 Hz, 3″, 5″-H); 7.12–7.10 (d, 2H, *J* = 8.4 Hz, 2′, 6′-H); 6.83–6.81 (d, 2H, *J* = 8.4 Hz, 3′, 5′-H); 6.35 (s, 1H, C–H); 3.68 (s, 3H, 4′-OCH_3_); 1.86 (s, 3H, CH_3_); ^13^C NMR (100 MHz, DMSO-*d*_6_) *δ*: 193.88, 159.39, 150.49, 147.55, 143.63, 140.12, 133.85, 132.23, 130.52, 128.93, 126.05, 114.27 107.56, 60.62, 55.55, and 19.70; MS (EI) *m*/*z*: calcd for C_20_H_17_ClN_4_O_2_: 380.1040; found: 381.2947 [M + 1]^+^, 382.2982 [M + 2]^+^, and elemental analysis: calcd for C_20_H_17_FN_4_O_2_: C, 63.08%; H, 4.50%; and N, 14.71%. Found: C, 62.97%; H, 4.21%; and N, 14.49%.

#### 4.2.11. (4″-Bromophenyl)(7-(4′methoxyphenyl)-5-methyl-4,7-dihydro-[1,2,4]triazolo[1,5-*a*]pyrimidin-6-yl)methanone (5k)

White solid; m.p. 215 °C; yield 88%; IR (KBr) (*ν*_max_/cm^−1^): 1628 (C=O); ^1^H NMR (400 MHz, DMSO-*d*_6_) *δ*: 10.75 (s, 1H, NH); 7.67 (s, 1H, triazole 3-H); 7.54–7.52 (d, 2H, *J* = 8.8 Hz, 2″, 6″-H); 7.50–7.48 (d, 2H, *J* = 8.8 Hz, 3″, 5″-H); 7.12–7.10 (d, 2H, *J* = 8.4 Hz, 2′, 6′-H); 6.83–6.80 (d, 2H, *J* = 8.8 Hz, 3′, 5′-H); 6.39 (s, 1H, C–H); 3.68 (s, 3H, 4′-OCH_3_); 1.86 (s, 3H, CH_3_); ^13^C NMR (100 MHz, DMSO-*d*_6_) *δ*: 193.76, 159.39, 150.48, 147.55, 143.58, 139.76, 137.06, 133.85, 130.38, 129.30, 128.94, 114.28 107.58, 60.64, 55.55, and 19.68; MS (EI) *m*/*z*: calcd for C_20_H_17_BrN_4_O_2_: 424.0535; found: 425.0521 [M + 1]^+^, 426.0512 [M + 2]^+^, and elemental analysis: calcd for C_20_H_17_FN_4_O_2_: C, 56.48%; H, 4.03%; and N, 13.17%. Found: C, 56.03%; H, 3.98%; and N, 13.01%.

#### 4.2.12. (4″-Methoxyphenyl)(7-(4′-methoxyphenyl)-5-methyl-4,7-dihydro-[1,2,4]triazolo[1,5-*a*]pyrimidin-6-yl)methanone (5l)

White solid; m.p. 220 °C; yield 95%; IR (KBr) (*ν*_max_/cm^−1^): 1659 (C=O), 3402 (NH); ^1^H NMR (400 MHz, DMSO-*d*_6_) *δ*: 10.69 (s, 1H, NH); 7.64 (s, 1H, triazole 3-H); 7.55–7.53 (d, 2H, *J* = 8.8 Hz, 2″, 6″-H); 7.11–7.08 (d, 2H, *J* = 8.4 Hz, 2′, 6′-H); 6.97–6.95 (d, 2H, *J* = 8.8 Hz, 3″, 5″-H); 6.81–6.79 (d, 2H, *J* = 8.8 Hz, 3′, 5′-H); 6.35 (s, 1H, C–H); 3.80 (s, 3H, 4″-OCH_3_); 3.67 (s, 3H, 4′-OCH_3_); 1.82 (s, 3H, CH_3_); ^13^C NMR (100 MHz, DMSO-*d*_6_) *δ*: 193.00, 162.22, 158.68, 149.74, 147.66, 140.57, 133.30, 132.56, 130.44, 128.23, 113.77, 113.59, 106.96, 60.35, 55.31, 54.90, and 18.86; MS (EI) *m*/*z*: calcd for C_21_H_20_N_4_O_3_: 376.1535; found: 377.1422 [M + 1]^+^, and elemental analysis: calcd for C_21_H_20_N_4_O_3_: C, 67.01%; H, 5.36%; and N, 14.88%. Found: C, 66.91%; H, 5.08%; and N, 14.53%.

#### 4.2.13. (3″-Methoxyphenyl)(7-(4′-methoxyphenyl)-5-methyl-4,7-dihydro-[1,2,4]triazolo[1,5-*a*]pyrimidin-6-yl)methanone (5m)

White solid; m.p. 210.5 °C; yield 92%; IR (KBr) (*ν*_max_/cm^−1^): 1628 (C=O); ^1^H NMR (400 MHz, DMSO-*d*_6_) *δ*: 10.68 (s, 1H, NH); 7.67 (s, 1H, triazole 3-H); 7.36–7.32 (t, 1H, *J* = 8 Hz, 5″-H); 7.12–7.06 (m, 4H, 2′, 6′-H, 2″, 6″-H); 6.96–6.95 (t, 1H, *J* = 8 Hz, 4″-H); 6.84–6.81 (d, 2H, *J* = 8.8 Hz, 3′, 5′-H); 6.36 (s, 1H, C–H); 3.75 (s, 3H, 3″-OCH_3_); 3.68 (s, 3H, 4′-OCH_3_); 1.84 (s, 3H, CH_3_); ^13^C NMR (100 MHz, DMSO-*d*_6_) *δ*: 194.73, 159.80, 159.38, 150.48, 147.68, 143.02, 142.64, 133.97, 130.35, 128.89, 120.74, 118.16, 114.27, 112.88, 107.92, 60.65, 55.79, 55.56, and 19.41; MS (EI) *m*/*z*: calcd. For C_21_H_20_N_4_O_3_: 376.1535; found: 377.1637 [M + 1]^+^, and elemental analysis: calcd for C_21_H_20_N_4_O_3_: C, 67.01%; H, 5.36%; and N, 14.88%. Found: C, 67.11%; H, 5.29%; and N, 14.76%.

#### 4.2.14. (7-(4′-Methoxyphenyl)-5-methyl-4,7-dihydro-[1,2,4]triazolo[1,5-*a*]pyrimidin-6-yl)(thiophen-2″-yl)methanone (5n)

White solid; m.p. 225 °C; yield 82%; IR (KBr) (*ν*_max_/cm^−1^): 1628 (C=O); ^1^H NMR (400 MHz, DMSO-*d*_6_) *δ*: 10.53 (s, 1H, NH); 7.93–7.91 (dd, 1H, *J*_1_ = 4.8 Hz, *J*_1_ = 0.8 Hz, 5″-H); 7.74–7.73 (dd, 1H, *J*_1_ = 4 Hz, *J*_1_ = 0.8 Hz, 3″-H); 7.64 (s, 1H, triazole 3-H); 7.16–7.12 (m, 3H, 4″-H, 2′, 6′-H); 6.82–6.79 (d, 2H, *J* = 8.8 Hz, 3′, 5′-H); 6.40 (s, 1H, C–H); 3.67 (s, 3H, 4′-OCH_3_); 1.96 (s, 3H, CH_3_); ^13^C NMR (100 MHz, DMSO-*d*_6_) *δ*: 187.03, 159.45, 150.38, 147.81, 145.34, 139.95, 134.92, 134.07, 133.39, 129.13, 128.94, 114.26, 107.74, 61.05, 55.54, and 19.16; MS (EI) *m*/*z*: calcd for C_18_H_16_ N_4_O_2_S: 352.0994; found: 353.1012 [M + 1]^+^, and elemental analysis: calcd for C_18_H_16_ N_4_O_2_S: C, 61.35%; H, 4.58%; and N, 15.90%. Found: C, 61.22%; H, 4.27%; and N, 15.78%.

#### 4.2.15. (5-Methyl-7-(4′-nitrophenyl)-4,7-dihydro-[1,2,4]triazolo[1,5-*a*]pyrimidin-6-yl)(phenyl)methanone (5o)

White solid; m.p. 204 °C; yield 80%; IR (KBr) (*ν*_max_/cm^−1^): 1628 (C=O), 3450 (NH); ^1^H NMR (400 MHz, DMSO-*d*_6_) *δ*: 10.95 (s, 1H, NH); 8.17–8.15 (d, 2H, *J* = 8.4 Hz, 3′, 5′-H); 7.73 (s, 1H, triazole 3-H); 7.55–7.53 (d, 2H, *J* = 8.8 Hz, 2′, 6′-H); 7.52–7.50 (m, 3H, 3″, 4″,5″-H); 7.45–7.42 (t, 2H, *J* = 7.2 Hz, 2″,6″-H); 6.58 (s, 1H, C–H); 1.82 (s, 3H, CH_3_); ^13^C NMR (100 MHz, DMSO-*d*_6_) *δ*: 194.66, 150.97, 148.70, 147.59, 145.05, 141.30, 132.33, 129.22, 129.19, 128.37, 124.24, 106.83, 60.54, and 19.99; MS (EI) *m*/*z*: calcd for C_19_H_15_N_5_O_3_: 361.1175; found: 362.1158 [M + 1]^+^, and elemental analysis: calcd for C_19_H_15_N_5_O_3_: C, 63.15%; H, 4.18%; and N, 19.38%. Found: C, 63.29%; H, 4.27%; and N, 19.23%.

#### 4.2.16. (4″-Fluorophenyl)(5-methyl-7-(4′-nitrophenyl)-4,7-dihydro-[1,2,4]triazolo[1,5-*a*]pyrimidin-6-yl)methanone (5p)

White solid; m.p. 236 °C; yield 83%; IR (KBr) (*ν*_max_/cm^−1^): 1628 (C=O), 3495 (NH); ^1^H NMR (400 MHz, DMSO-*d*_6_) *δ*: 10.96 (s, 1H, NH); 8.16–8.14 (d, 2H, *J* = 8.8 Hz, 3′, 5′-H); 7.73 (s, 1H, triazole 3-H); 7.63–7.60 (dt, 2H, *J* = 7.2 Hz, *J* = 5.6 Hz, 2″, 6″-H); 7.55–7.53 (d, 2H, *J* = 8.4 Hz, 2′, 6′-H); 7.28–7.24 (t, 2H, *J* = 8.8 Hz, 3″, 5″-H); 6.57 (s, 1H, C–H); 1.85 (s, 3H, CH_3_); ^13^C NMR (100 MHz, DMSO-*d*_6_) *δ*: 193.32, 165.89–163.41 (d, ^1^*J*_C–F_ = 248 Hz, Ph-4″-C), 150.96, 148.64, 147.69, 147.61, 144.98, 137.70, 137.67, 131.36–131.27 (d, ^3^*J*_C–F_ = 9 Hz, Ph-2″, 6″-C), 129.20, 124.24, 116.37–116.16 (d, ^2^*J*_C–F_ = 21 Hz, Ph-3″, 5″-C), 106.63, 60.59, and 20.04; MS (EI) *m*/*z*: calcd for C_19_H_14_FN_5_O_3_: 379.1081; found: 380.1068 [M + 1]^+^, and elemental analysis: calcd for C_19_H_14_FN_5_O_3_: C, 60.16%; H, 3.72%; and N, 18.46%. Found: C, 60.27%; H, 3.57%; and N, 18.23%.

#### 4.2.17. (4″-Chlorophenyl)(5-methyl-7-(4′-nitrophenyl)-4,7-dihydro-[1,2,4]triazolo[1,5-*a*]pyrimidin-6-yl)methanone (5q)

White solid; m.p. 213 °C; yield 82%; IR (*ν*_max_/cm^−1^): 1628 (C=O); ^1^H NMR (400 MHz, DMSO-*d*_6_) *δ*: 11.02 (s, 1H, NH); 8.17–8.15 (d, 2H, *J* = 8.8 Hz, 3′, 5′-H); 7.74 (s, 1H, triazole 3-H); 7.57–7.49 (m, 6H, 2′, 6′, 2″, 6″, 3″, 5″-H); 6.58 (s, 1H, C–H); 1.85 (s, 3H, CH_3_); ^13^C NMR (100 MHz, DMSO-*d*_6_) *δ*: 193.45, 150.97, 148.66, 147.62, 145.67, 139.95, 137.08, 130.37, 129.35, 129.19, 124.24, 106.60, 60.46, and 20.18; MS (EI) *m*/*z*: calcd for C_19_H_14_ClN_5_O_3_: 395.0785; found: 396.0791 [M + 1]^+^, and elemental analysis: calcd for C_19_H_14_ClN_5_O_3_: C, 57.66%; H, 3.57%; and N, 17.69%. Found: C, 57.52%; H, 3.41%; and N, 17.75%.

#### 4.2.18. (4″-Bromophenyl)(5-methyl-7-(4′-nitrophenyl)-4,7-dihydro-[1,2,4]triazolo[1,5-*a*]pyrimidin-6-yl)methanone (5r)

White solid; m.p. 220 °C; yield 80%; IR (KBr) (*ν*_max_/cm^−1^): 1628 (C=O); ^1^H NMR (400 MHz, DMSO-*d*_6_) *δ*: 11.00 (s, 1H, NH); 8.14–8.16 (d, 2H, *J* = 8.8 Hz, 3′, 5′-H); 7.73 (s, 1H, triazole 3-H); 7.65–7.63 (d, 2H, *J* = 8.4 Hz, 2″, 6″-H); 7.55–7.53 (d, 2H, *J* = 8.8 Hz, 2′, 6′-H); 7.48–7.45 (d, 2H, *J* = 8.8 Hz, 3″, 5″-H); 6.55 (s, 1H, C–H); 1.85 (s, 3H, CH_3_); ^13^C NMR (100 MHz, DMSO-*d*_6_) *δ*: 192.90, 150.35, 148.02, 146.98, 145.07, 139.69, 131.65, 129.88, 128.55, 125.44, 123.61, 105.97, 59.82, and 19.57; MS (EI) *m*/*z*: calcd for C_19_H_14_BrN_5_O_3_: 439.0280; found: 440.0268 [M + 1]^+^, and elemental analysis: calcd for C_19_H_14_BrN_5_O_3_: C, 51.84%; H, 3.21%; and N, 15.91%. Found: C, 51.68%; H, 3.34%; and N, 15.77%.

#### 4.2.19. (4″-Methoxyphenyl)(5-methyl-7-(4′-nitrophenyl)-4,7-dihydro-[1,2,4]triazolo[1,5-*a*]pyrimidin-6-yl)methanone (5s)

White solid; m.p. 241 °C; yield 89%; IR (KBr) (*ν*_max_/cm^−1^): 1628 (C=O); ^1^H NMR (400 MHz, DMSO-*d*_6_) *δ*: 10.76 (s, 1H, NH); 8.16–8.13 (d, 2H, *J* = 8.8 Hz, 2″, 6″-H); 7.72 (s, 1H, triazole 3-H); 7.57–7.55 (d, 2H, *J* = 9.2 Hz, 2′, 6′-H); 7.52–7.50 (d, 2H, *J* = 8.8 Hz, 3″, 5″-H); 6.98–6.95 (d, 2H, *J* = 9.2 Hz, 3′, 5′-H); 6.57 (s, 1H, C–H); 3.81 (s, 3H, 4″-OCH_3_); 1.84 (s, 3H, CH_3_); ^13^C NMR (100 MHz, DMSO-*d*_6_) *δ*: 193.53, 163.04, 150.89, 148.53, 148.01, 147.59, 142.31, 133.06, 131.10, 129.14, 124.80, 124.23, 114.49, 106.64, 60.85, 55.96, and 19.64; MS (EI) *m*/*z*: calcd for C_20_H_17_N_5_O_4_: 391.1281; found: 392.1272 [M + 1]^+^, and elemental analysis: calcd for C_20_H_17_N_5_O_4_: C, 61.38%; H, 4.38%; and N, 17.89%. Found: C, 61.25%; H, 4.22%; and N, 17.75%.

#### 4.2.20. (3″-Methoxyphenyl)(5-methyl-7-(4′-nitrophenyl)-4,7-dihydro-[1,2,4]triazolo[1,5-*a*]pyrimidin-6-yl)methanone (5t)

White solid; m.p. 202.5 °C; yield 85%; IR (KBr) (*ν*_max_/cm^−1^): 1628 (C=O); ^1^H NMR (400 MHz, DMSO-*d*_6_) *δ*: 10.94 (s, 1H, NH); 8.17–8.15 (d, 2H, *J* = 8.4 Hz, 3′, 5′-H); 7.73 (s, 1H, triazole 3-H); 7.55–7.53 (d, 2H, *J* = 8.8 Hz, 2′, 6′-H); 7.36–7.32 (t, 1H, *J* = 8 Hz, 5″-H); 7.08 (m, 2H, 4″, 6″-H); 6.99–6.98 (dd, 1H, *J* = 2.4 Hz, *J* = 1.2 Hz, 2″-H); 6.57 (s, 1H, C–H); 3.76 (s, 3H, 3″-OCH_3_); 1.85 (s, 3H, CH_3_); ^13^C NMR (100 MHz, DMSO-*d*_6_) *δ*: 194.33, 159.85, 150.96, 148.74, 147.69, 147.60, 145.23, 142.80, 130.40, 129.18, 124.22, 120.69, 118.16, 112.86, 106.87, 60.52, 55.82, and 19.88; MS (EI) *m*/*z*: calcd for C_20_H_17_N_5_O_4_: 391.1281; found: 392.1335 [M + 1]^+^, and elemental analysis: calcd for C_20_H_17_N_5_O_4_: C, 61.38%; H, 4.38%; and N, 17.89%. Found: C, 61.25%; H, 4.25%; and N, 17.72%.

#### 4.2.21. 5-Methyl-7-(4′-nitrophenyl)-4,7-dihydro-[1,2,4]triazolo[1,5-*a*]pyrimidin-6-yl)(thiophen-2″-yl)methanone (5u)

White solid; m.p. 222 °C; yield 78%; IR (KBr) (*ν*_max_/cm^−1^): 1628 (C=O); ^1^H NMR (400 MHz, DMSO-*d*_6_) *δ*: 10.79 (s, 1H, NH); 8.15–8.13 (d, 2H, *J* = 9.2 Hz, 3′, 5′-H); 7.94–7.93 (d, 1H, *J* = 6 Hz, 5″-H); 7.77–7.75 (d, 1H, *J* = 4.8 Hz, 3″-H); 7.71 (s, 1H, triazole 3-H); 7.53–7.51 (d, 2H, *J* = 8.8 Hz, 2′, 6′-H); 7.16–7.14 (t, 1H, *J*_1_ = 4.8 Hz, *J*_2_ = 4 Hz, 4″-H); 6.61 (s, 1H, C–H); 1.98 (s, 3H, CH_3_); ^13^C NMR (100 MHz, DMSO-*d*_6_) *δ*: 186.63, 150.89, 148.15, 147.95, 147.69, 145.16, 141.72, 135.17, 134.34, 129.30, 128.99, 124.25, 106.57, 60.83, and 19.56; MS (EI) *m*/*z*: calcd for C_17_H_13_N_5_O_3_S: 367.0739; found: 368.0716 [M + 1]^+^, and elemental analysis: calcd for C_17_H_13_N_5_O_3_S: C, 55.58%; H, 3.57%; and N, 19.06%. Found: C, 55.29%; H, 3.41%; and N, 18.99%.

### 4.3. Binding studies

#### 4.3.1. Docking studies

Molecular docking studies were conducted using the software AutoDock Vina and the Lamarckian Genetic Algorithm (LGA) to evaluate the binding affinity of the newly synthesized compounds towards BSA. The crystallographic structure of the BSA protein (PDB ID: 4f5s) was obtained from the RCSB Protein Data Bank (http://www.rcsb.org/PDB). The stages of molecular docking include protein identification and preparation, ligand preparation and grid generation. The protein file was opened in Discovery Studio Visualizer, where heteroatoms and water molecules were removed. Polar hydrogens were added and Kollman charges were assigned, and the prepared protein structure was then saved in PDB format. The 3D structures of the ligands (5a–u) were prepared in Chem3D by energy minimization using the force field MMFF94 with the maximum number of iteration of 500 and a minimum RMS gradient of 0.1. The protein file was then prepared for docking by removing water molecules and packing with polar hydrogen with Kollman charges. After preparing the PDBQT files of protein and ligands, the ligands were docked with BSA with defined grid parameters. The grid box dimensions (93.95 Å × 61.72 Å × 84.48 Å) centered at (9.93, 20.81, and 99.21) were generated to cover the whole protein structure of BSA. The docked conformation with the lowest energy was selected for additional examination of the docking simulations, and the outcomes were examined and visualized using Discovery Studio Visualizer.

#### 4.3.2. BSA‐binding studies

BSA was purchased directly from Sigma Aldrich Company and used without further purification. For binding interaction studies, analytical grade reagents were used. A BSA stock solution of 15 µM (1 mg/1 mL) concentration was prepared in 10 mM phosphate buffer saline (prepared using Na_2_HPO_4_ and NaH_2_PO_4_) of pH 7.2, and then kept at 2–6 °C for further use. Stock solutions of [1,2,4]triazolo[1,5-*a*]pyrimidine (5f, 5m and 5t) of 1 mM concentration were prepared in DMSO as a solvent and further diluted with the buffer as per requirement depending upon the mode of interaction study.

Fluorescence emission spectroscopy was used for site marker investigation at an excitation wavelength of 280 nm. Phenylbutazone (PBZ) and ibuprofen (IBP) were utilized as particular site-specific probes in this experiment. Solutions of each site marker (15 µM) were prepared separately in PBS. Equimolar BSA was then added to each probe solution at a 1 : 1 (v/v) ratio, and the mixtures were maintained at 2–6 °C. The BSA + site marker binary solution was subsequently titrated with increasing concentrations of compound 5t (0–40 µM), while keeping the concentrations of BSA + IBP and BSA + PBZ constant.

## Author contributions

Ranjana Aggarwal: supervision, writing – review & editing, resources, conceptualization, data curation. Manisha Sharma: writing – original draft, methodology, investigation, data curation. Garima Sumran: writing – original draft, methodology, formal analysis, visualization. Suresh Kumar: investigation, software, visualization. Parvin Kumar: supervision, writing – review & editing, conceptualization.

## Conflicts of interest

The author(s) confirm that this article's content has no conflicts of interest.

## Supplementary Material

RA-016-D5RA09673A-s001

## Data Availability

The data for this article have been included as part of the supplementary information (SI). Supplementary information: experimental data (^1^H, ^13^C, [^1^H–^13^C] HMBC, [^1^H–^13^C] HSQC, [^1^H–^15^N] HMBC and HRMS) for final compounds, as well as 2D and 3D diagrams showing the interactions of ligands 5a–u, PBZ and IBP with BSA protein. See DOI: https://doi.org/10.1039/d5ra09673a.
