# Supplementary material for: Regioselective synthesis, structural investigation and binding behavior of 6-aroyl-7-aryl-5-methyl-4,7-dihydro-[1,2,4]triazolo[1,5-a]pyrimidines with BSA using various spectroscopic and in silico methods
Source: RSC Adv. 2026 Mar 10;16(15):13454–70. doi: 10.1039/d5ra09673a (PMC12974574; doi:10.1039/d5ra09673a)

Supplementary Information (SI) for RSC Advances.
This journal is © The Royal Society of Chemistry 2026

SUPPLEMENTARY DATA

Regioselective Synthesis, Structural Investigation and Binding Behavior of 6-Aroyl-7-aryl-5-methyl-4,7-dihydro-[1,2,4]triazolo[1,5-*a*]pyrimidines with BSA using Various Spectroscopic and *In Silico* Methods

Ranjana Aggarwal,[a,b]* Manisha Sharma,[a] Garima Sumran[c], Suresh Kumar[d], Parvin Kumar[a]

[a] Department of Chemistry, Kurukshetra University, Kurukshetra-136119, Haryana, India

[b] Council of Scientific and Industrial Research (CSIR) HQ, Vigyan Suchna Bhawan, 14, Satsang Vihar Marg, New Delhi-110067, India

[c] Department of Chemistry, D. A. V. College (Lahore), Ambala City, Haryana 134 003, India

[d] Department of Chemistry, SUS Govt. College, Matak Majri, Karnal, Haryana, 132041, India

*Corresponding author:

Prof. Ranjana Aggarwal, Tel: +91-9896740740

E-mails: *ranjana67in@yahoo.com*, *ranjanaaggarwal67@gmail.com*

Table of Contents

1. **Table S1.** 2D and 3D diagram showing of the interaction of ligands **5a-u**, PBZ and IBP with BSA protein. S7-S24

2. **Table S2.** 2D and 3D diagram showing of the interaction of *R*- and *S*-enantiomers of compounds (**5a**, **5f**, **5m**, and **5t**) with BSA protein. S25-S28

3. NMR Data

- ^{1}H NMR spectrum of (5-Methyl-7-phenyl-4,7-dihydro-[1,2,4]triazolo[1,5-*a*]pyrimidin-6-yl)(phenyl)methanone **(5a)** S29
- ^{13}C NMR spectrum of (5-Methyl-7-phenyl-4,7-dihydro-[1,2,4]triazolo[1,5-*a*]pyrimidin-6-yl)(phenyl)methanone **(5a)** S30
- ^{1}H NMR spectrum of (4''-Fluorophenyl)(5-methyl-7-phenyl-4,7-dihydro-[1,2,4]triazolo[1,5-*a*]pyrimidin-6-yl)methanone **(5b)** S31
- ^{13}C NMR spectrum of (4''-Fluorophenyl)(5-methyl-7-phenyl-4,7-dihydro-[1,2,4]triazolo[1,5-*a*]pyrimidin-6-yl)methanone **(5b)** S32
- ^{1}H NMR spectrum of (4''-Chlorophenyl)(5-methyl-7-phenyl-4,7-dihydro-[1,2,4]triazolo[1,5-*a*]pyrimidin-6-yl)methanone **(5c)** S33
- ^{13}C NMR spectrum of (4''-Chlorophenyl)(5-methyl-7-phenyl-4,7-dihydro-[1,2,4]triazolo[1,5-*a*]pyrimidin-6-yl)methanone **(5c)** S34
- HRMS of (4''-Chlorophenyl)(5-methyl-7-phenyl-4,7-dihydro-[1,2,4]triazolo[1,5-*a*]pyrimidin-6-yl)methanone **(5c)** S35
- ^{1}H NMR spectrum of (4''-Bromophenyl)(5-methyl-7-phenyl-4,7-dihydro-[1,2,4]triazolo[1,5-*a*]pyrimidin-6-yl)methanone **(5d)** S36
- ^{13}C NMR spectrum of (4''-Bromophenyl)(5-methyl-7-phenyl-4,7-dihydro-[1,2,4]triazolo[1,5-*a*]pyrimidin-6-yl)methanone **(5d)** S37
- ^{1}H NMR spectrum of (4''-Methoxyphenyl)(5-methyl-7-phenyl-4,7-dihydro-[1,2,4]triazolo[1,5-*a*]pyrimidin-6-yl)methanone **(5e)** S38

- ^{13}C NMR spectrum of (4''-Methoxyphenyl)(5-methyl-7-phenyl-4,7-dihydro-[1,2,4]triazolo[1,5-*a*]pyrimidin-6-yl)methanone **(5e)** S39
- ^{1}H NMR spectrum of (3''-Methoxyphenyl)(5-methyl-7-phenyl-4,7-dihydro-[1,2,4]triazolo[1,5-*a*]pyrimidin-6-yl)methanone **(5f)** S40
- ^{13}C NMR spectrum of (3''-Methoxyphenyl)(5-methyl-7-phenyl-4,7-dihydro-[1,2,4]triazolo[1,5-*a*]pyrimidin-6-yl)methanone **(5f)** S41
- HRMS of (3''-Methoxyphenyl)(5-methyl-7-phenyl-4,7-dihydro-[1,2,4]triazolo[1,5-*a*]pyrimidin-6-yl)methanone **(5f)** S42
- ^{1}H NMR spectrum of (5-Methyl-7-phenyl-4,7-dihydro-[1,2,4]triazolo[1,5-*a*]pyrimidin-6-yl)(thiophen-2''-yl)methanone **(5g)** S43
- ^{13}C NMR spectrum of (5-Methyl-7-phenyl-4,7-dihydro-[1,2,4]triazolo[1,5-*a*]pyrimidin-6-yl)(thiophen-2''-yl)methanone **(5g)** S44
- ^{1}H NMR spectrum of (7-(4'-Methoxyphenyl)-5-methyl-4,7-dihydro-[1,2,4]triazolo[1,5-*a*]pyrimidin-6-yl)(phenyl)methanone **(5h)** S45
- ^{13}C NMR spectrum of (7-(4'-Methoxyphenyl)-5-methyl-4,7-dihydro-[1,2,4]triazolo[1,5-*a*]pyrimidin-6-yl)(phenyl)methanone **(5h)** S46
- ^{1}H NMR spectrum of (4''-Fluorophenyl)(7-(4'-methoxyphenyl)-5-methyl-4,7-dihydro-[1,2,4]triazolo[1,5-*a*]pyrimidin-6-yl)methanone **(5i)** S47
- ^{13}C NMR spectrum of (4''-Fluorophenyl)(7-(4'-methoxyphenyl)-5-methyl-4,7-dihydro-[1,2,4]triazolo[1,5-*a*]pyrimidin-6-yl)methanone **(5i)** S48
- ^{1}H NMR spectrum of (4''-Chlorophenyl)(7-(4'-methoxyphenyl)-5-methyl-4,7-dihydro-[1,2,4]triazolo[1,5-*a*]pyrimidin-6-yl)methanone **(5j)** S49

- ^{13}C NMR spectrum of (4''-Chlorophenyl)(7-(4'-methoxyphenyl)-5-methyl-4,7-dihydro-[1,2,4]triazolo[1,5-*a*]pyrimidin-6-yl)methanone **(5j)** S50
- HRMS of (4''-Chlorophenyl)(7-(4'-methoxyphenyl)-5-methyl-4,7-dihydro-[1,2,4]triazolo[1,5-*a*]pyrimidin-6-yl)methanone **(5j)** S51
- ^{1}H NMR spectrum of (4''-Bromophenyl)(7-(4'-methoxyphenyl)-5-methyl-4,7-dihydro-[1,2,4]triazolo[1,5-*a*]pyrimidin-6-yl)methanone **(5k)** S52
- ^{13}C NMR spectrum of (4''-Bromophenyl)(7-(4'-methoxyphenyl)-5-methyl-4,7-dihydro-[1,2,4]triazolo[1,5-*a*]pyrimidin-6-yl)methanone **(5k)** S53
- ^{1}H NMR spectrum of (4''-Methoxyphenyl)(7-(4'-methoxyphenyl)-5-methyl-4,7-dihydro-[1,2,4]triazolo[1,5-*a*]pyrimidin-6-yl)methanone **(5l)** S54
- ^{13}C NMR spectrum of (4''-Methoxyphenyl)(7-(4'-methoxyphenyl)-5-methyl-4,7-dihydro-[1,2,4]triazolo[1,5-*a*]pyrimidin-6-yl)methanone **(5l)** S55
- ^{1}H NMR spectrum of (3''-Methoxyphenyl)(7-(4'-methoxyphenyl)-5-methyl-4,7-dihydro-[1,2,4]triazolo[1,5-*a*]pyrimidin-6-yl)methanone **(5m)** S56
- ^{13}C NMR spectrum of (3''-Methoxyphenyl)(7-(4'-methoxyphenyl)-5-methyl-4,7-dihydro-[1,2,4]triazolo[1,5-*a*]pyrimidin-6-yl)methanone **(5m)** S57
- HRMS of (3''-Methoxyphenyl)(7-(4'-methoxyphenyl)-5-methyl-4,7-dihydro-[1,2,4]triazolo[1,5-*a*]pyrimidin-6-yl)methanone **(5m)** S58
- ^{1}H NMR spectrum of (7-(4'-Methoxyphenyl)-5-methyl-4,7-dihydro-[1,2,4]triazolo[1,5-*a*]pyrimidin-6-yl)(thiophen-2''-yl)methanone **(5n)** S59
- ^{13}C NMR spectrum of (7-(4'-Methoxyphenyl)-5-methyl-4,7-dihydro-[1,2,4]triazolo[1,5-*a*]pyrimidin-6-yl)(thiophen-2''-yl)methanone **(5n)** S60

- ^{1}H NMR spectrum of (5-Methyl-7-(4'-nitrophenyl)-4,7-dihydro-[1,2,4]triazolo[1,5-*a*]pyrimidin-6-yl)(phenyl)methanone **(5o)** S61
- ^{13}C NMR spectrum of (5-Methyl-7-(4'-nitrophenyl)-4,7-dihydro-[1,2,4]triazolo[1,5-*a*]pyrimidin-6-yl)(phenyl)methanone **(5o)** S62
- ^{1}H NMR spectrum of (4''-Fluorophenyl)(5-methyl-7-(4'-nitrophenyl)-4,7-dihydro-[1,2,4]triazolo[1,5-*a*]pyrimidin-6-yl)methanone **(5p)** S63
- ^{13}C NMR spectrum of (4''-Fluorophenyl)(5-methyl-7-(4'-nitrophenyl)-4,7-dihydro-[1,2,4]triazolo[1,5-*a*]pyrimidin-6-yl)methanone **(5p)** S64
- ^{1}H NMR spectrum of (4''-Chlorophenyl)(5-methyl-7-(4'-nitrophenyl)-4,7-dihydro-[1,2,4]triazolo[1,5-*a*]pyrimidin-6-yl)methanone **(5q)** S65
- ^{13}C NMR spectrum of (4''-Chlorophenyl)(5-methyl-7-(4'-nitrophenyl)-4,7-dihydro-[1,2,4]triazolo[1,5-*a*]pyrimidin-6-yl)methanone **(5q)** S66
- ^{1}H NMR spectrum of (4''-Bromophenyl)(5-methyl-7-(4'-nitrophenyl)-4,7-dihydro-[1,2,4]triazolo[1,5-*a*]pyrimidin-6-yl)methanone **(5r)** S67
- ^{13}C NMR spectrum of (4''-Bromophenyl)(5-methyl-7-(4'-nitrophenyl)-4,7-dihydro-[1,2,4]triazolo[1,5-*a*]pyrimidin-6-yl)methanone **(5r)** S68
- ^{1}H NMR spectrum of (4''-Methoxyphenyl)(5-methyl-7-(4'-nitrophenyl)-4,7-dihydro-[1,2,4]triazolo[1,5-*a*]pyrimidin-6-yl)methanone **(5s)** S69
- ^{13}C NMR spectrum of (4''-Methoxyphenyl)(5-methyl-7-(4'-nitrophenyl)-4,7-dihydro-[1,2,4]triazolo[1,5-*a*]pyrimidin-6-yl)methanone **(5s)** S70
- ^{1}H NMR spectrum of (3''-Methoxyphenyl)(5-methyl-7-(4'-nitrophenyl)-4,7-dihydro-[1,2,4]triazolo[1,5-*a*]pyrimidin-6-yl)methanone **(5t)** S71

- ^{13}C NMR spectrum of (3''-Methoxyphenyl)(5-methyl-7-(4'-nitrophenyl)-4,7-dihydro-[1,2,4]triazolo[1,5-*a*]pyrimidin-6-yl)methanone **(5t)** S72
- HRMS of (3''-Methoxyphenyl)(5-methyl-7-(4'-nitrophenyl)-4,7-dihydro-[1,2,4]triazolo[1,5-*a*]pyrimidin-6-yl)methanone **(5t)** S73
- ^{1}H NMR spectrum of 5-Methyl-7-(4'-nitrophenyl)-4,7-dihydro-[1,2,4]triazolo[1,5-*a*]pyrimidin-6-yl)(thiophen-2''-yl)methanone **(5u)** S74
- ^{13}C NMR spectrum of 5-Methyl-7-(4'-nitrophenyl)-4,7-dihydro-[1,2,4]triazolo[1,5-*a*]pyrimidin-6-yl)(thiophen-2''-yl)methanone **(5u)** S75

3. 2D NMR

- **Fig. S1.** ^{1}H-^{13}C HMBC NMR spectrum of (4''-Methoxyphenyl)(7-(4'-methoxyphenyl)-5-methyl-4,7-dihydro-[1,2,4]triazolo[1,5-*a*]pyrimidin-6-yl)methanone **(5l)** S76
- **Fig. S2.** ^{1}H-^{13}C HSQC NMR spectrum of (4''-Methoxyphenyl)(7-(4'-methoxyphenyl)-5-methyl-4,7-dihydro-[1,2,4]triazolo[1,5-*a*]pyrimidin-6-yl)methanone **(5l)** S77
- **Fig. S3.** ^{1}H-^{15}N HMBC NMR spectrum of (4''-Methoxyphenyl)(7-(4'-methoxyphenyl)-5-methyl-4,7-dihydro-[1,2,4]triazolo[1,5-*a*]pyrimidin-6-yl)methanone **(5l)** S78

Table S1. 2D and 3D diagram showing of the interaction of ligands **5a-u**, PBZ and IBP with BSA protein.

S. no.	Compound	Dock Score/ Binding Energy	2D	3D
1.	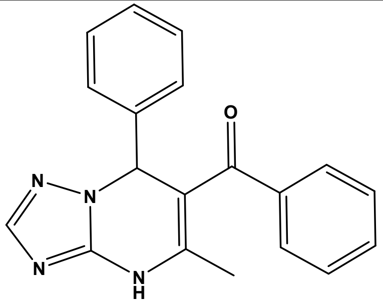	-7.4	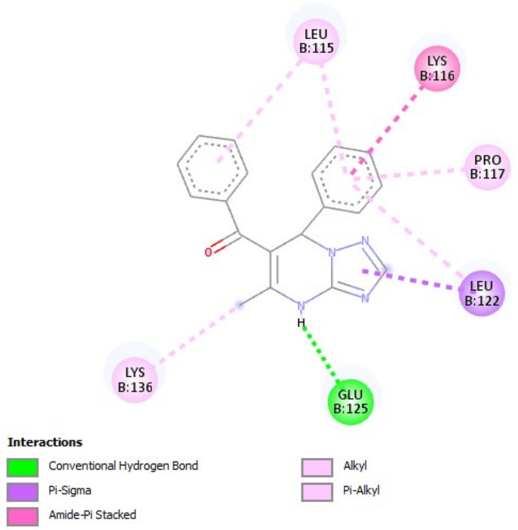	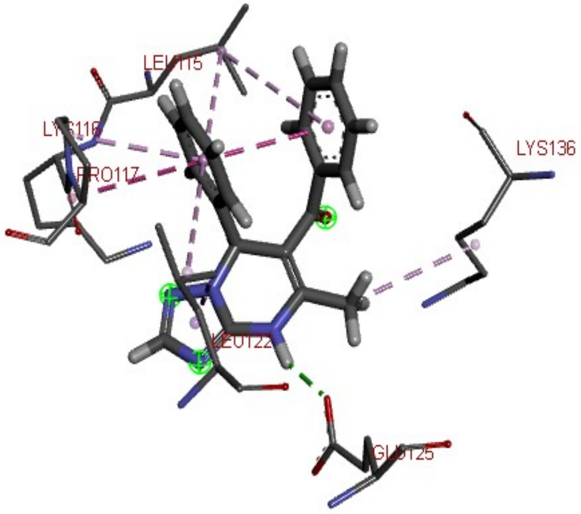

2.	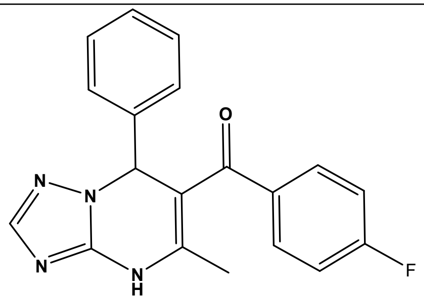O<br>N<br>N<br>N<br>N<br>H<br>F	-7.6	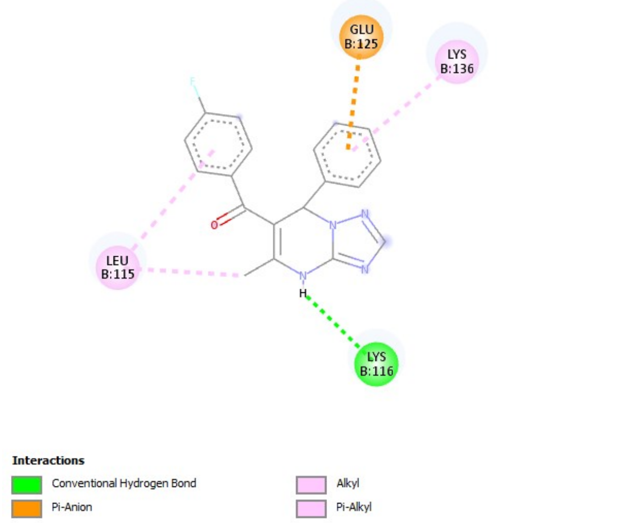GLU B:125<br>LYS B:136<br>LEU B:115<br>LYS B:116<br>Interactions<br>Conventional Hydrogen Bond<br>Pi-Anion<br>Alkyl<br>Pi-Alkyl	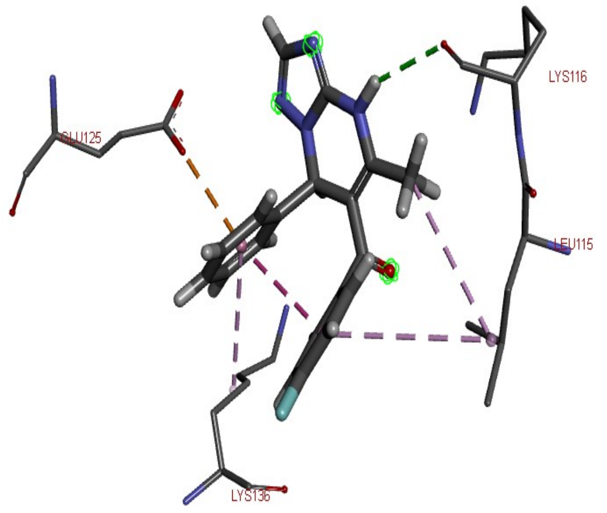LYS116<br>LEU115<br>LYS136
3.	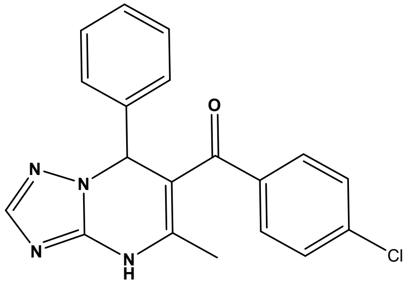O<br>N<br>N<br>N<br>N<br>H<br>Cl	-7.9	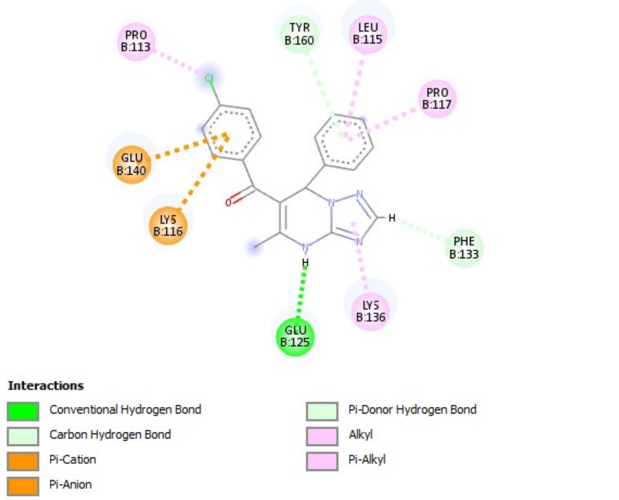PRO B:113<br>TYR B:160<br>LEU B:115<br>PRO B:117<br>GLU B:140<br>LYS B:116<br>PHE B:133<br>LYS B:136<br>GLU B:125<br>Interactions<br>Conventional Hydrogen Bond<br>Carbon Hydrogen Bond<br>Pi-Cation<br>Pi-Anion<br>Pi-Donor Hydrogen Bond<br>Alkyl<br>Pi-Alkyl	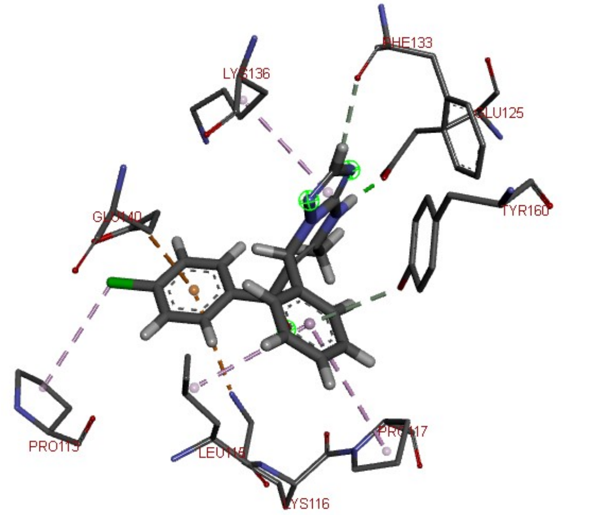TYR160

4.	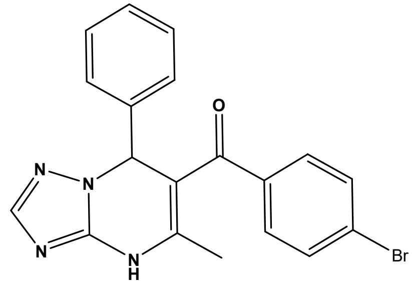 O<br>N<br>N<br>N<br>N<br>H<br>Br	-7.9	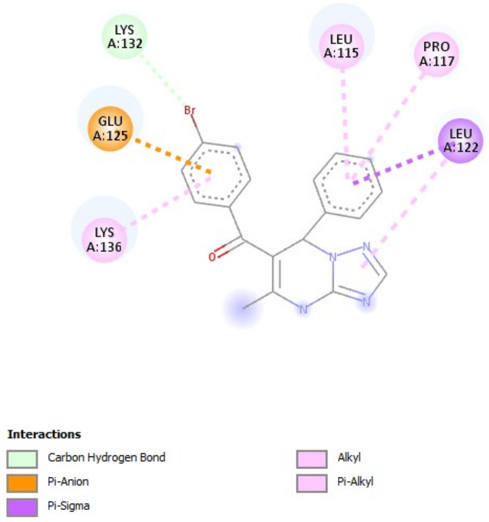 LYS A:132<br>LEU A:115<br>PRO A:117<br>GLU A:125<br>LEU A:122<br>LYS A:136<br>Br<br>Interactions<br>Carbon Hydrogen Bond<br>Pi-Anion<br>Pi-Sigma<br>Alkyl<br>Pi-Alkyl	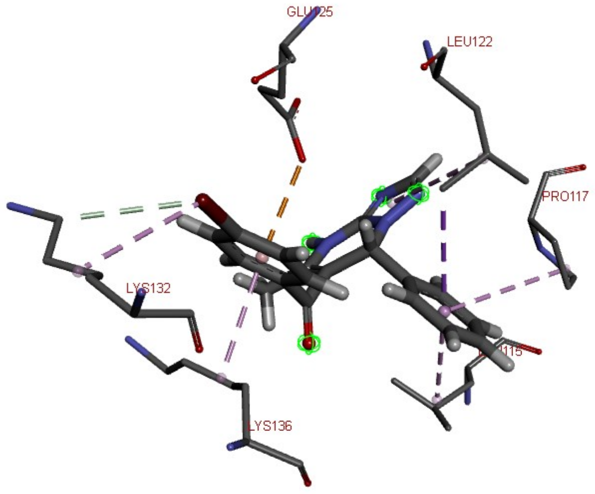 LEU122<br>PRO117<br>LYS136

5.	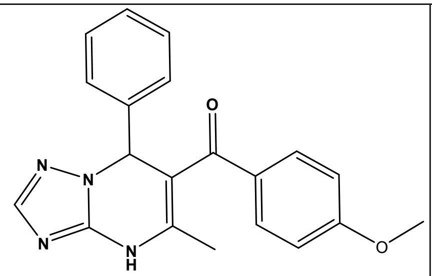 N N N O NH O	-7.4	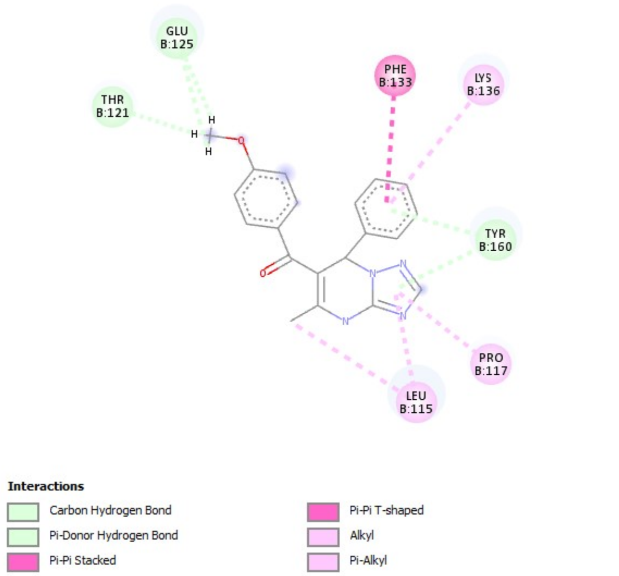 GLU B:125<br>THR B:121<br>PHE B:133<br>LYS B:136<br>TYR B:160<br>PRO B:117<br>LEU B:115<br>Interactions<br>Carbon Hydrogen Bond<br>Pi-Donor Hydrogen Bond<br>Pi-Pi Stacked<br>Pi-Pi T-shaped<br>Alkyl<br>Pi-Alkyl	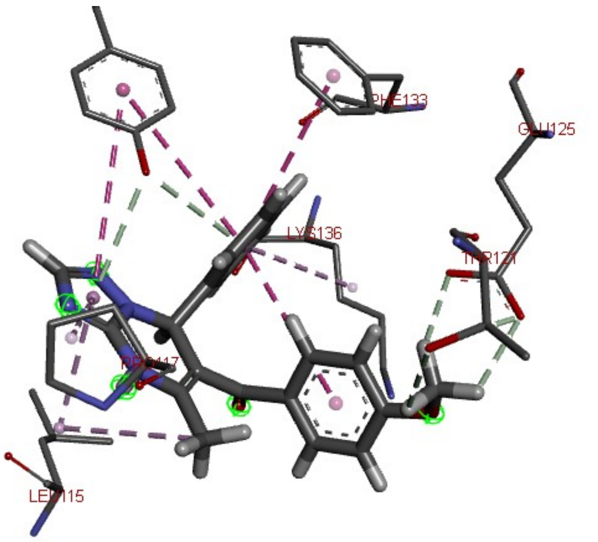
6.	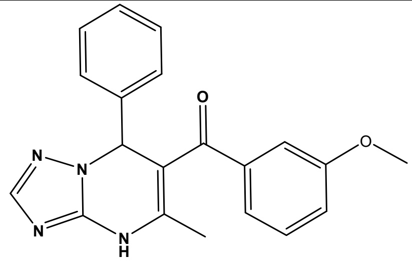 N N N O O NH	-7.9	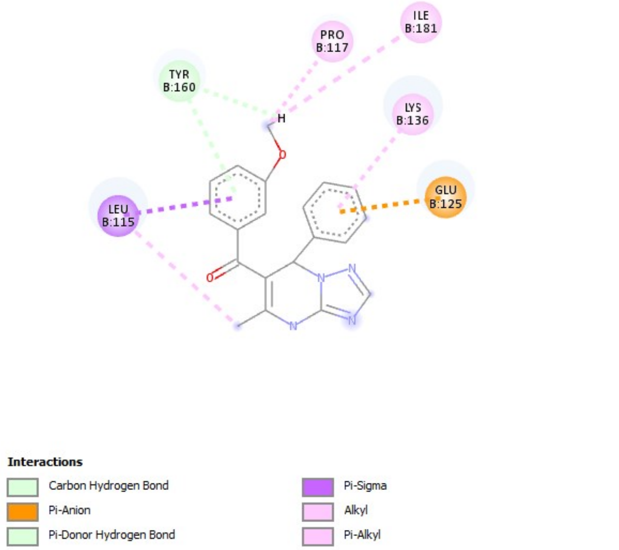 PRO B:117<br>ILE B:181<br>TYR B:160<br>LYS B:136<br>LEU B:115<br>GLU B:125<br>Interactions<br>Carbon Hydrogen Bond<br>Pi-Anion<br>Pi-Donor Hydrogen Bond<br>Pi-Sigma<br>Alkyl<br>Pi-Alkyl	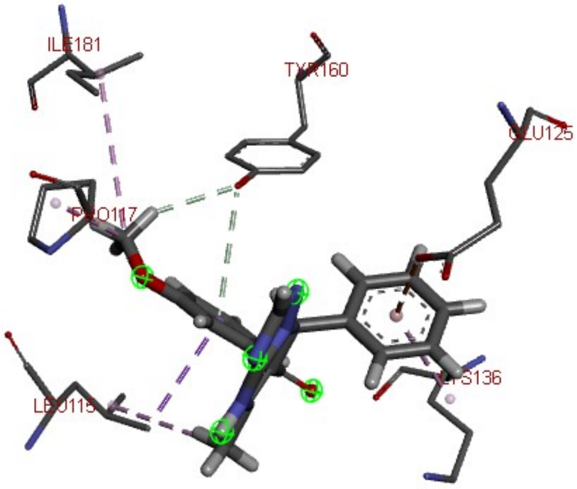 ILE181<br>TYR160<br>GLU125

7.	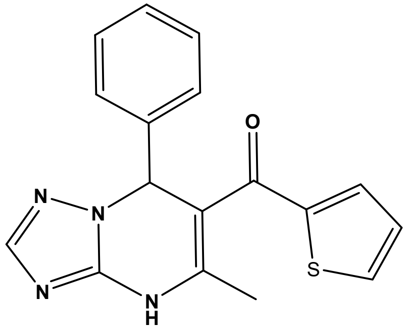	-7.1	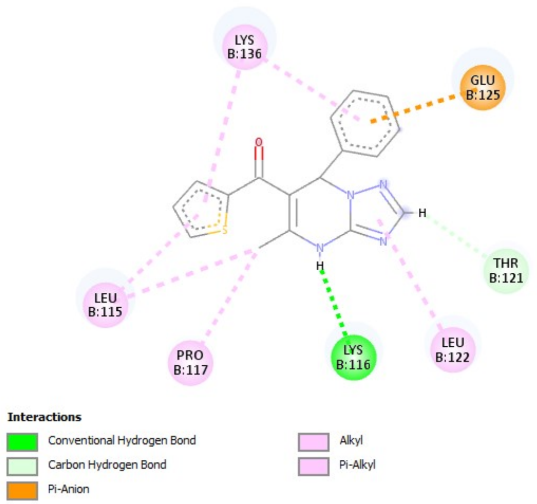LYS B:136<br>GLU B:125<br>THR B:121<br>LEU B:115<br>PRO B:117<br>LYS B:116<br>LEU B:122<br>Interactions<br>Conventional Hydrogen Bond<br>Carbon Hydrogen Bond<br>Pi-Anion<br>Alkyl<br>Pi-Alkyl	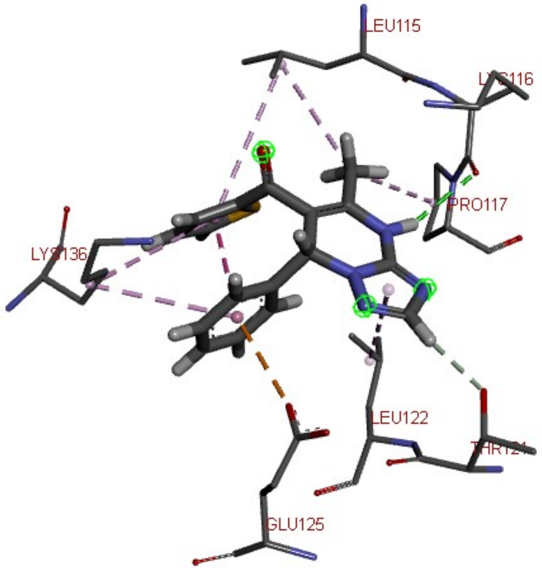LEU115<br>PRO117<br>LEU122<br>GLU125

8.	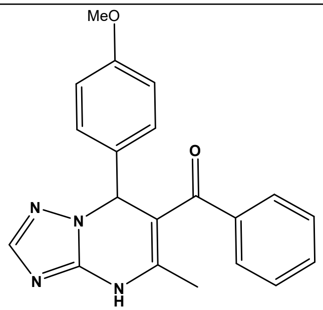MeO<br>O<br>N<br>N<br>N<br>H	-7.4	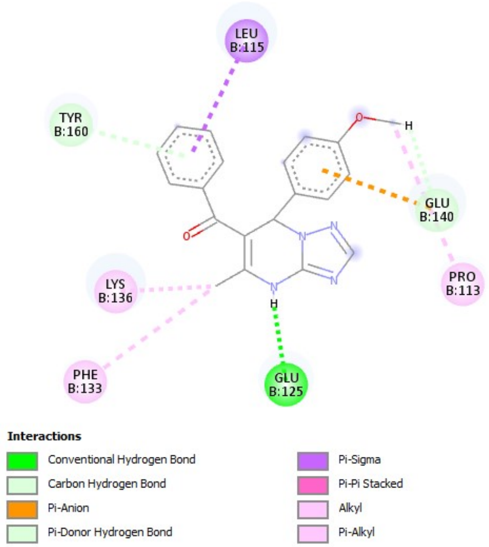LEU B:115<br>TYR B:160<br>GLU B:140<br>PRO B:113<br>LYS B:136<br>PHE B:133<br>GLU B:125<br>Interactions<br>Conventional Hydrogen Bond<br>Carbon Hydrogen Bond<br>Pi-Anion<br>Pi-Donor Hydrogen Bond<br>Pi-Sigma<br>Pi-Pi Stacked<br>Alkyl<br>Pi-Alkyl	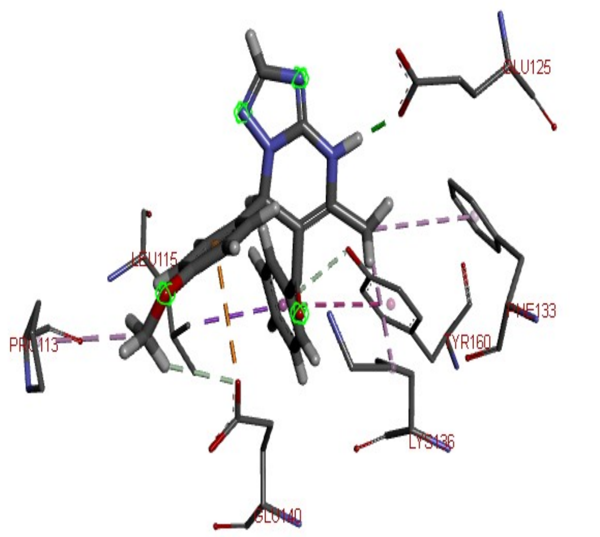PHE133<br>TYR160
9.	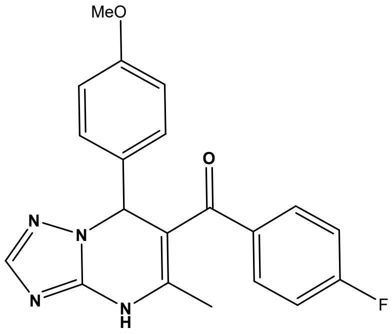MeO<br>O<br>N<br>N<br>N<br>H<br>F	-7.3	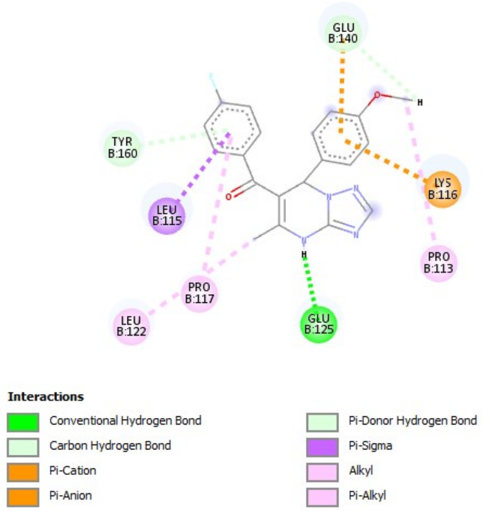GLU B:140<br>TYR B:160<br>LEU B:115<br>LYS B:116<br>PRO B:113<br>PRO B:117<br>LEU B:122<br>GLU B:125<br>Interactions<br>Conventional Hydrogen Bond<br>Carbon Hydrogen Bond<br>Pi-Cation<br>Pi-Anion<br>Pi-Donor Hydrogen Bond<br>Pi-Sigma<br>Alkyl<br>Pi-Alkyl	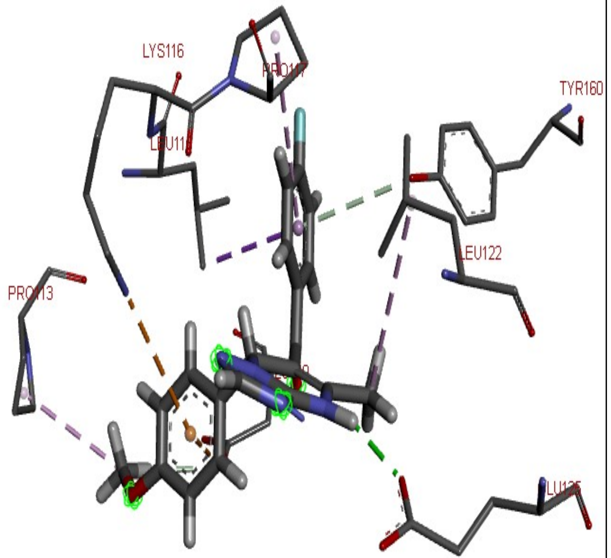LYS116<br>TYR160<br>LEU122

10.	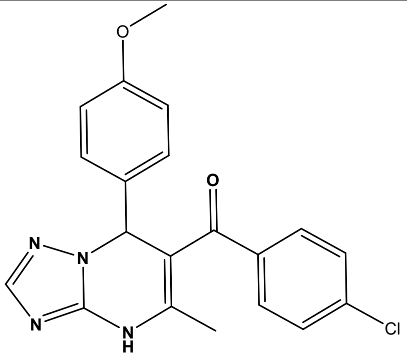	-6.4	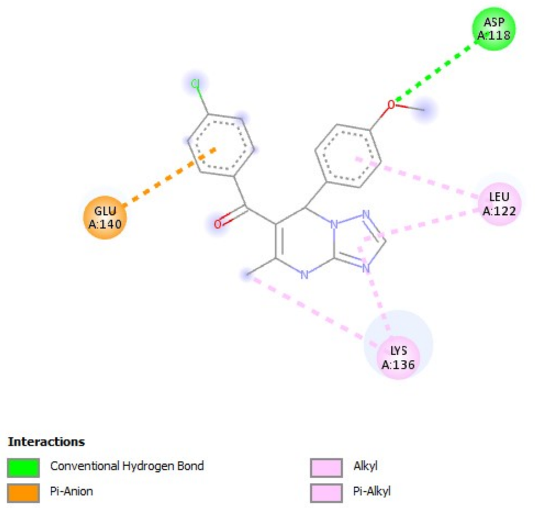ASP A:118<br>GLU A:140<br>LEU A:122<br>LYS A:136<br>Interactions<br>Conventional Hydrogen Bond<br>Pi-Anion<br>Alkyl<br>Pi-Alkyl	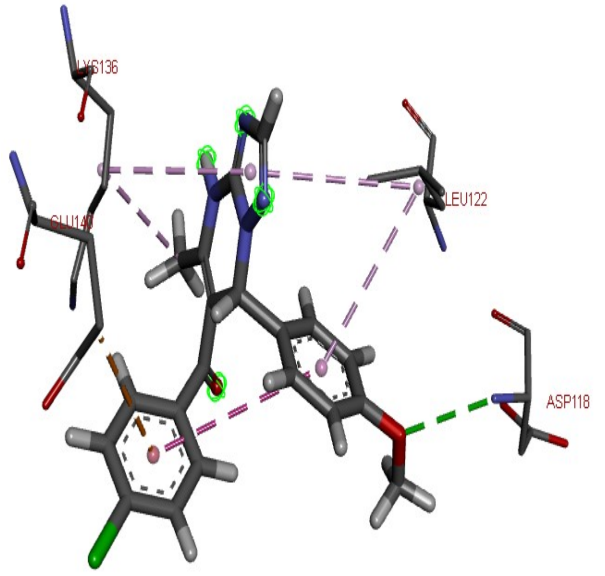LYS136<br>LEU122<br>ASP118

11.	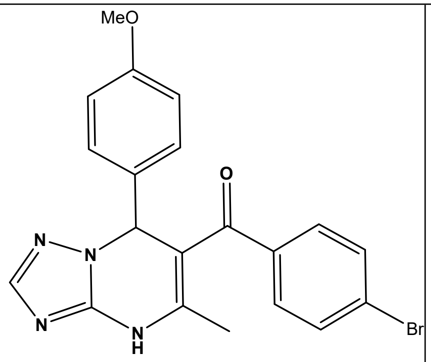MeO O N N N N H Br	-6.5	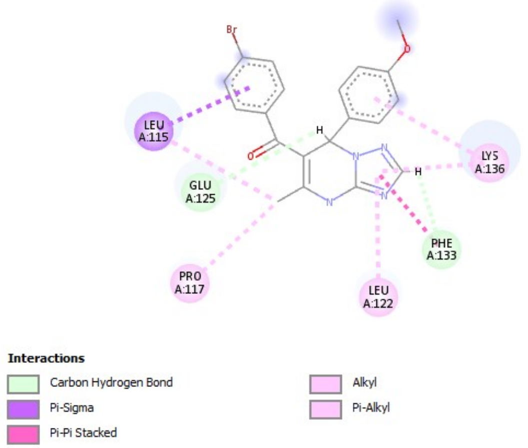LEU A:115 GLU A:125 PRO A:117 LEU A:122 PHE A:133 LYS A:136 Interactions Carbon Hydrogen Bond Pi-Sigma Pi-Pi Stacked Alkyl Pi-Alkyl	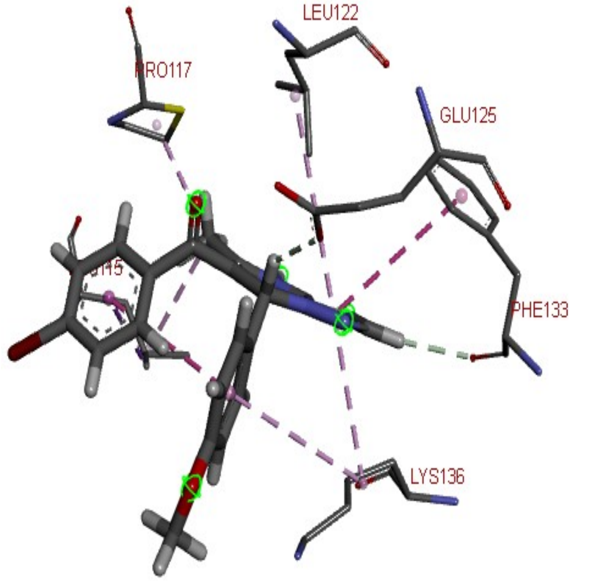PRO117 LEU122 GLU125 PHE133 LYS136

12.	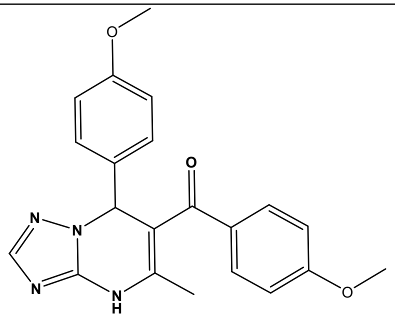	-6.4	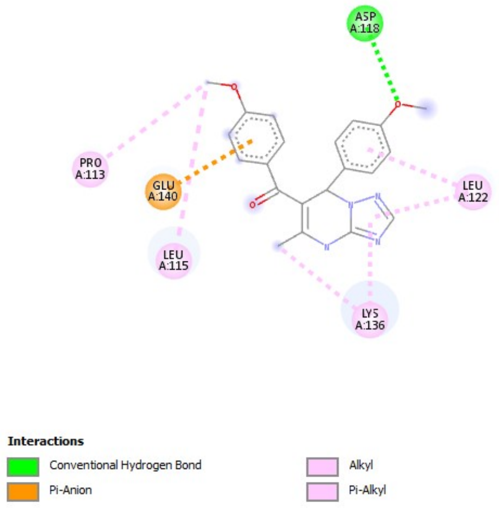ASP A:118<br>PRO A:113<br>GLU A:140<br>LEU A:122<br>LEU A:115<br>LYS A:136<br>Interactions<br>Conventional Hydrogen Bond<br>Pi-Anion<br>Alkyl<br>Pi-Alkyl	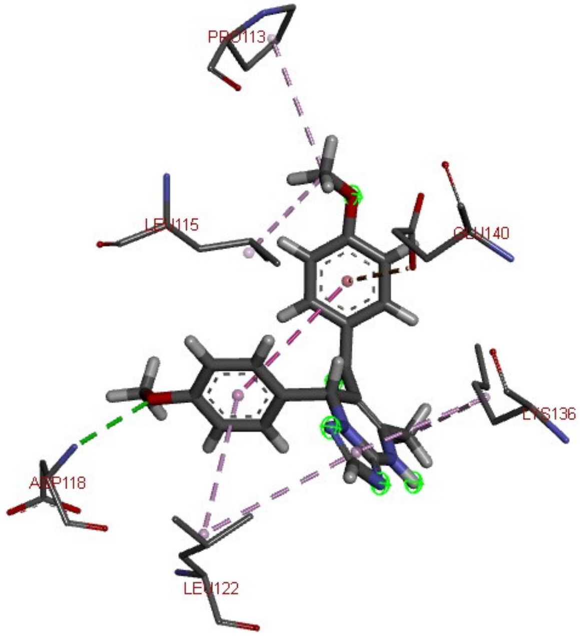

13.	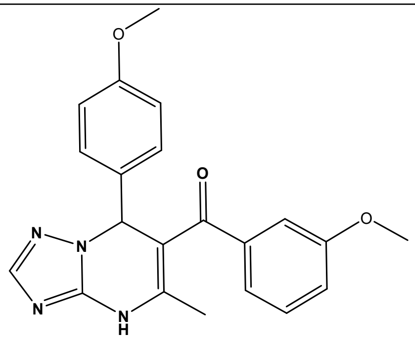	-7.0	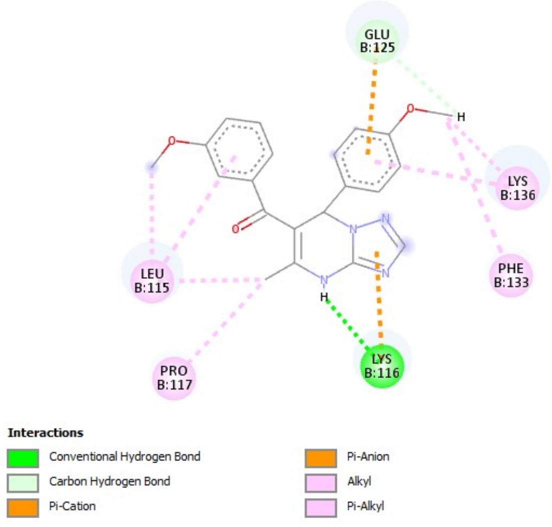GLU B:125 LYS B:136 PHE B:133 LEU B:115 PRO B:117 LYS B:116 Interactions Conventional Hydrogen Bond Carbon Hydrogen Bond Pi-Cation Pi-Anion Alkyl Pi-Alkyl	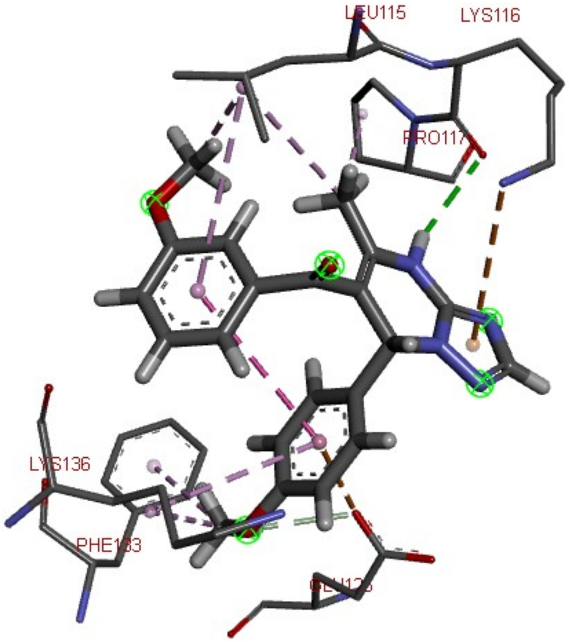LEU115 LYS116

14.	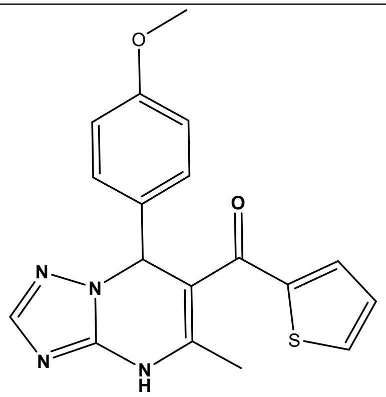	-6.0	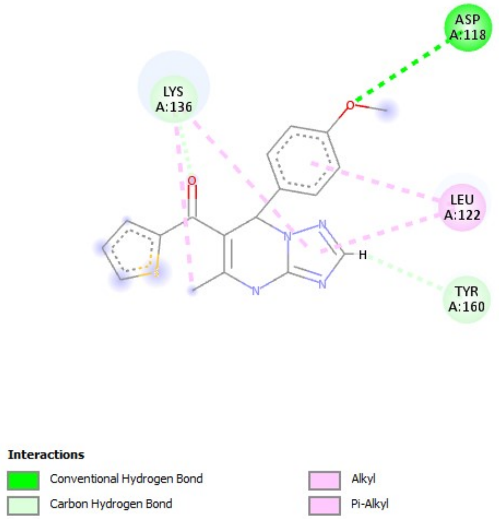ASP A:118<br>LYS A:136<br>LEU A:122<br>TYR A:160<br>Interactions<br>Conventional Hydrogen Bond<br>Carbon Hydrogen Bond<br>Alkyl<br>Pi-Alkyl	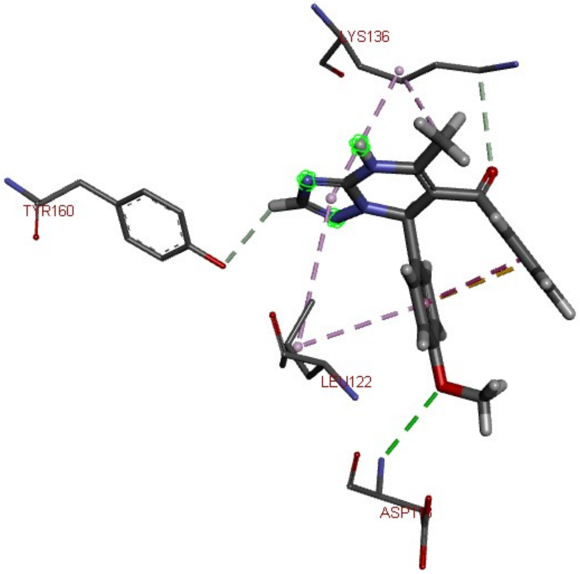

15.	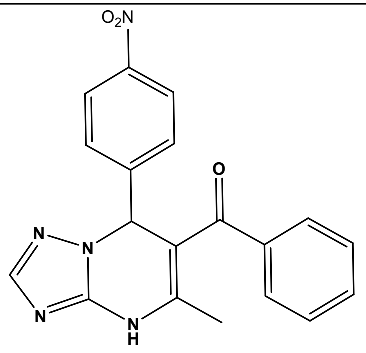	-6.4	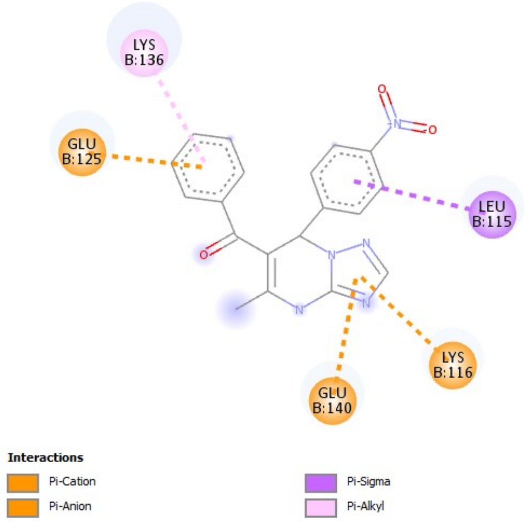LYS B:136<br>GLU B:125<br>LEU B:115<br>LYS B:116<br>GLU B:140<br>Interactions<br>Pi-Cation<br>Pi-Anion<br>Pi-Sigma<br>Pi-Alkyl	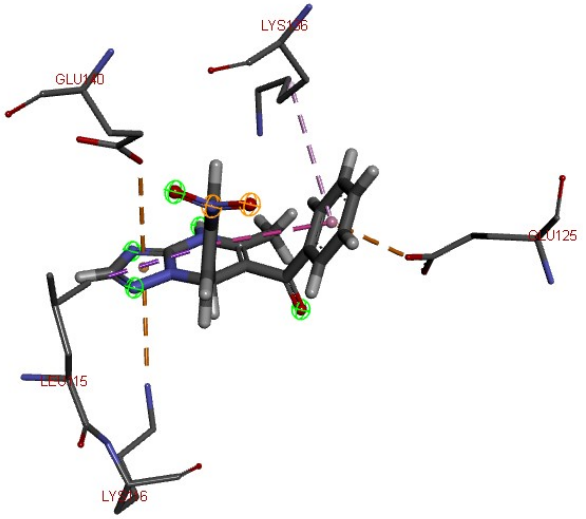

<table>
<tr>
<td>16.</td>
<td>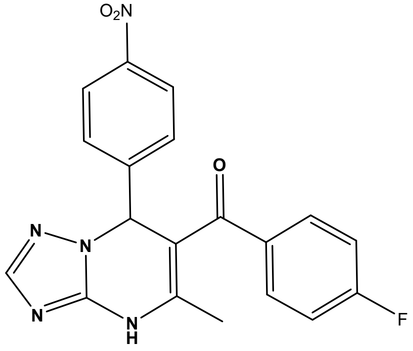</td>
<td>-7.5</td>
<td>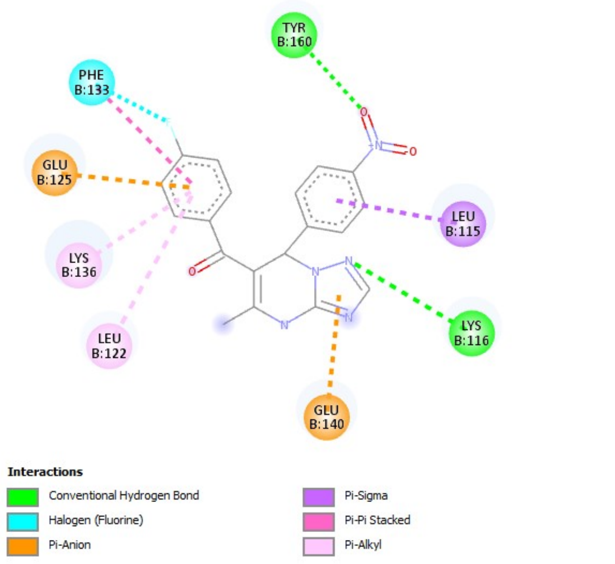</td>
<td>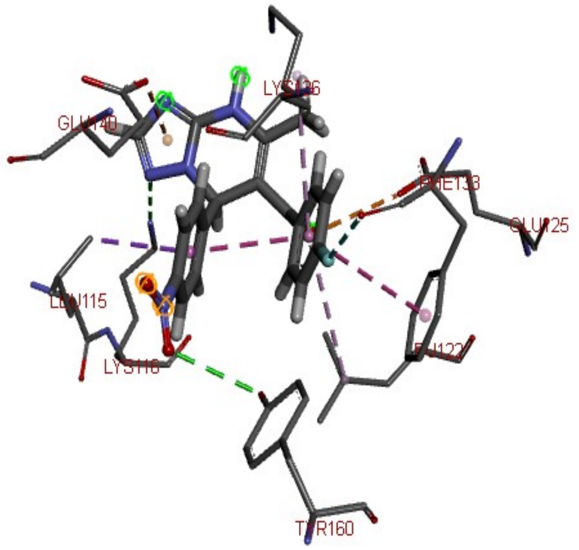</td>
</tr>
</table>

17.	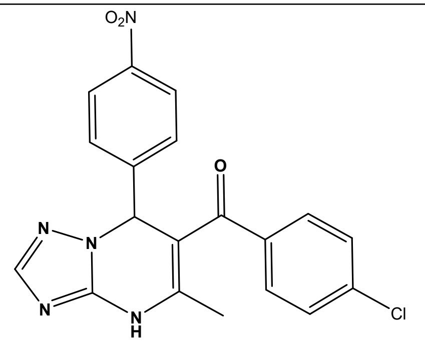	-6.6	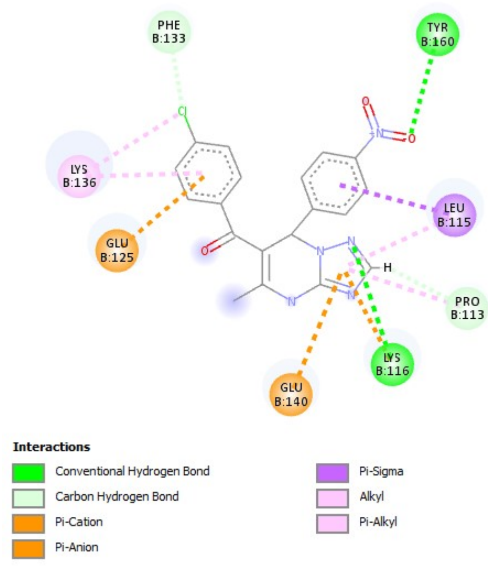PHE B:133<br>TYR B:160<br>LYS B:136<br>LEU B:115<br>GLU B:125<br>PRO B:113<br>LYS B:116<br>GLU B:140<br>Interactions<br>Conventional Hydrogen Bond<br>Carbon Hydrogen Bond<br>Pi-Cation<br>Pi-Anion<br>Pi-Sigma<br>Alkyl<br>Pi-Alkyl	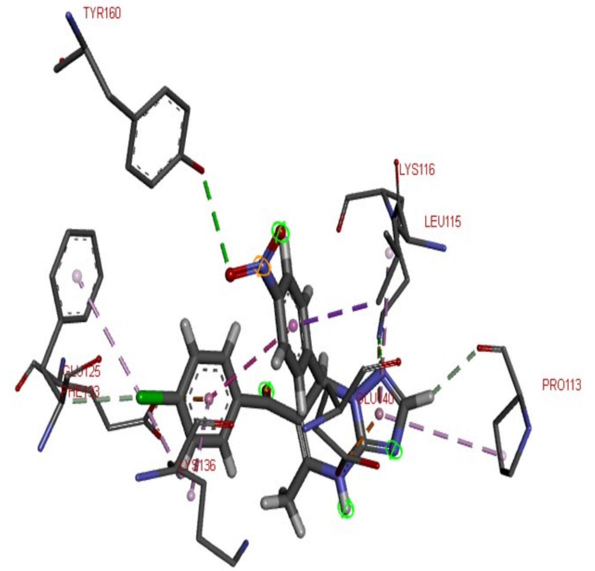TYR160<br>LYS116<br>LEU115<br>PRO113

<table>
<tr>
<td>18.</td>
<td>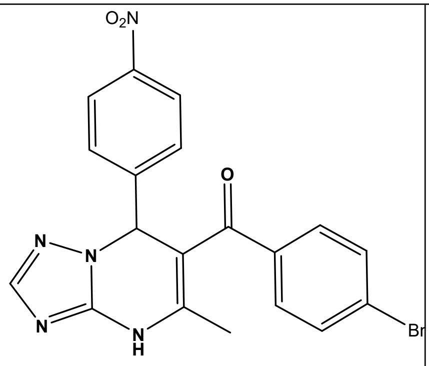</td>
<td>-7.3</td>
<td>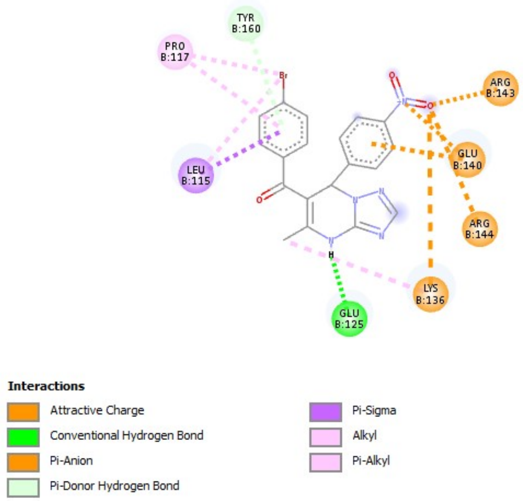
</td>
<td>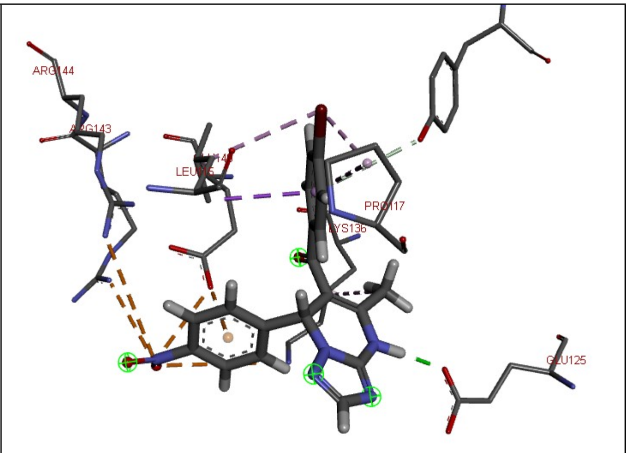
</td>
</tr>
</table>

19.	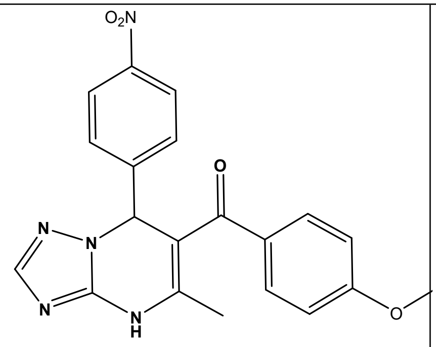	-6.2	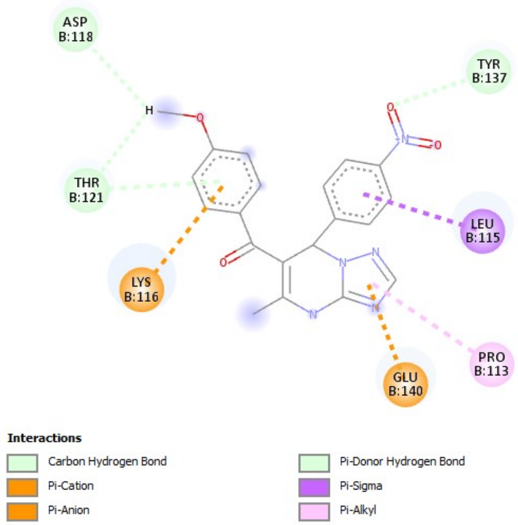ASP B:118<br>TYR B:137<br>THR B:121<br>LEU B:115<br>LYS B:116<br>PRO B:113<br>GLU B:140<br>Interactions<br>Carbon Hydrogen Bond<br>Pi-Cation<br>Pi-Anion<br>Pi-Donor Hydrogen Bond<br>Pi-Sigma<br>Pi-Alkyl	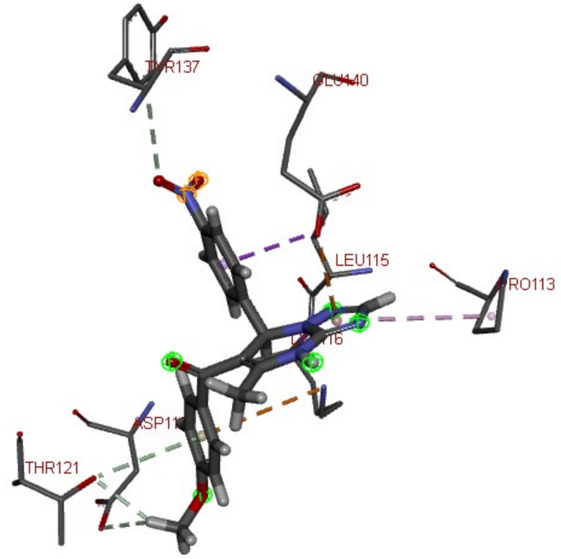LEU115<br>THR121

<table>
<tr>
<td>20.</td>
<td>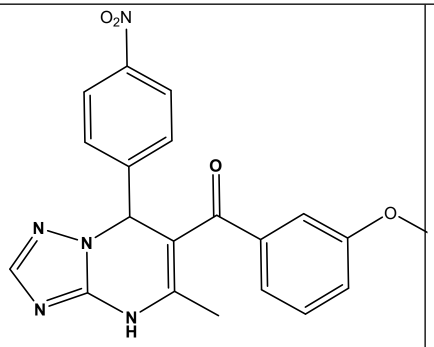
</td>
<td>-7.4</td>
<td>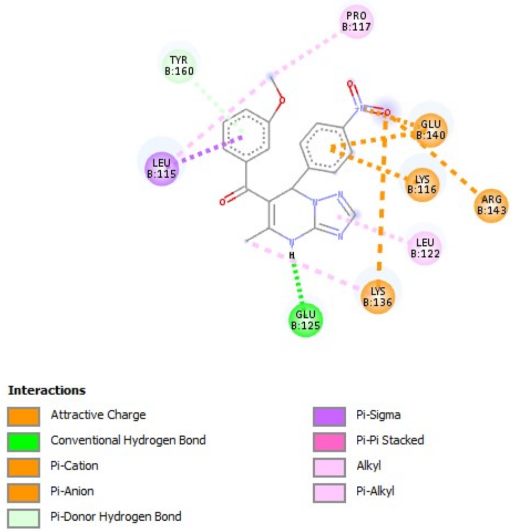
</td>
<td>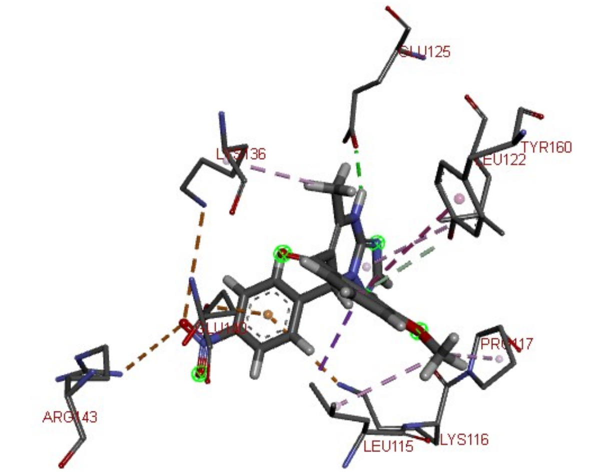
</td>
</tr>
<tr>
<td>21.</td>
<td>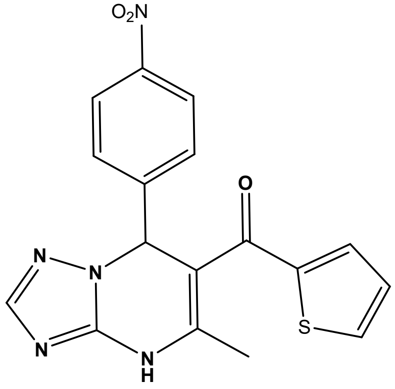
</td>
<td>-6.5</td>
<td>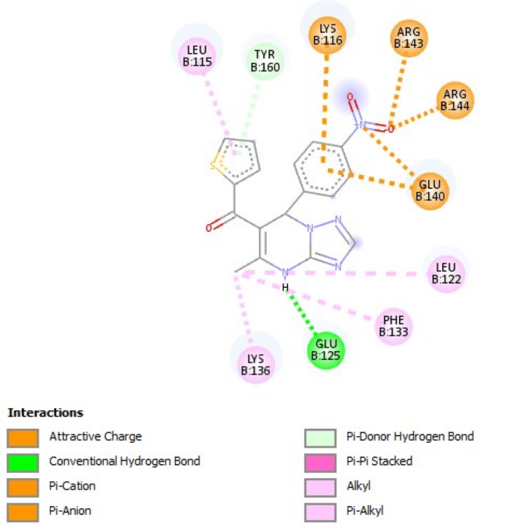
</td>
<td>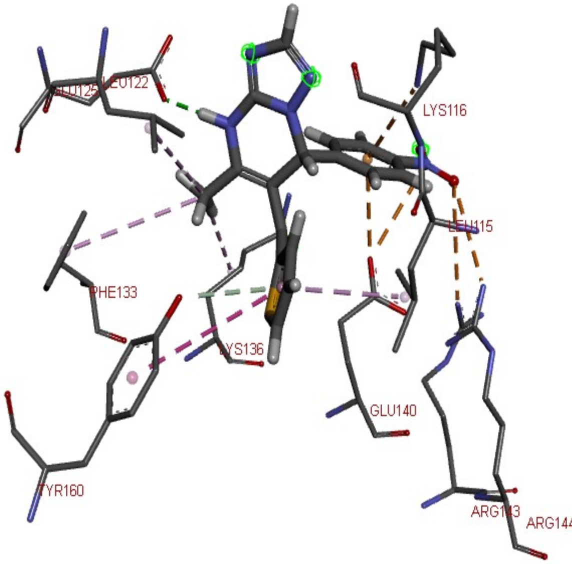
</td>
</tr>
</table>

22.	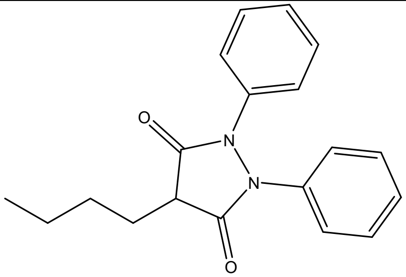	-8.1	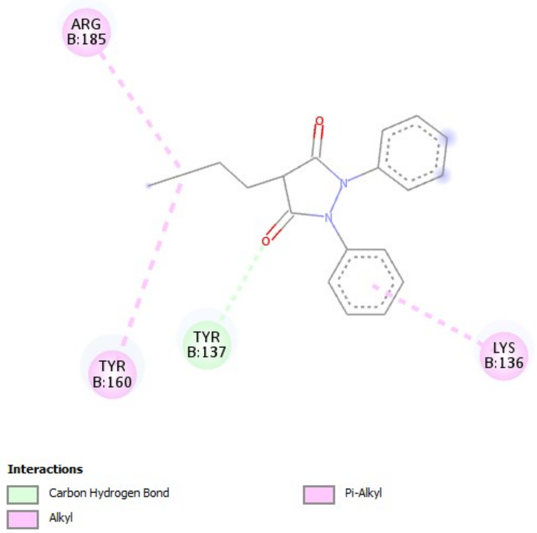ARG B:185<br>TYR B:160<br>TYR B:137<br>LYS B:136<br>Interactions<br>Carbon Hydrogen Bond<br>Alkyl<br>Pi-Alkyl	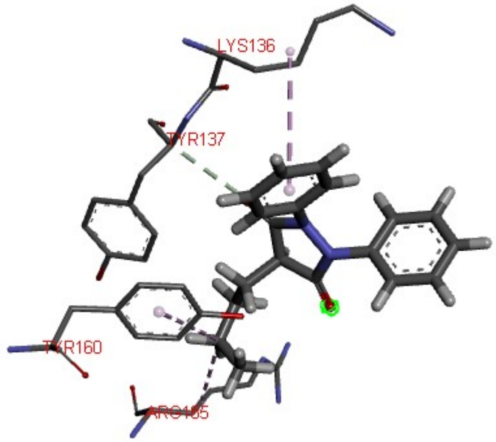LYS136<br>TYR137<br>TYR160
23.	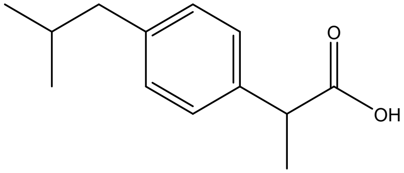	-6.6	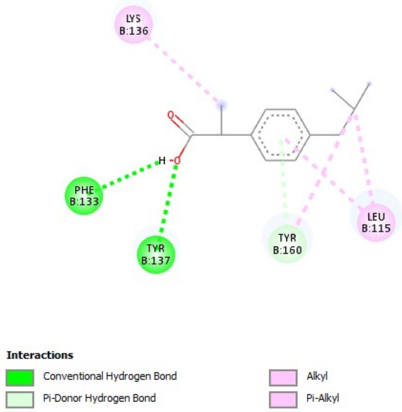LYS B:136<br>PHE B:133<br>TYR B:137<br>TYR B:160<br>LEU B:115<br>Interactions<br>Conventional Hydrogen Bond<br>Pi-Donor Hydrogen Bond<br>Alkyl<br>Pi-Alkyl	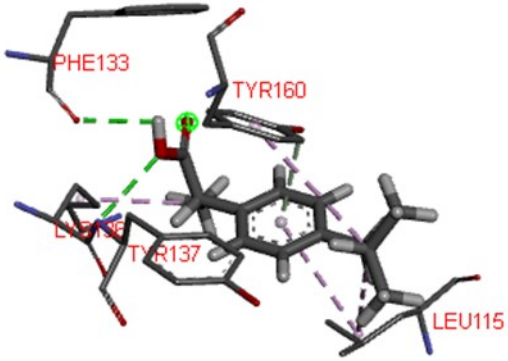PHE133<br>TYR160<br>TYR137<br>LEU115

Table S2. 2D and 3D diagram showing of the interaction of *R*- and *S*-enantiomers of compounds (**5a**, **5f**, **5m**, and **5t**) with BSA protein.

S. no.	Compound	Dock Score/ Binding Energy	2D	3D
1.	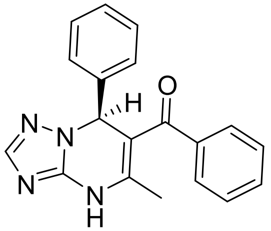 (*S*)-(5-methyl-7-phenyl-4,7-dihydro-[1,2,4]triazolo[1,5-*a*]pyrimidin-6-yl)(phenyl)methanone	-2.3	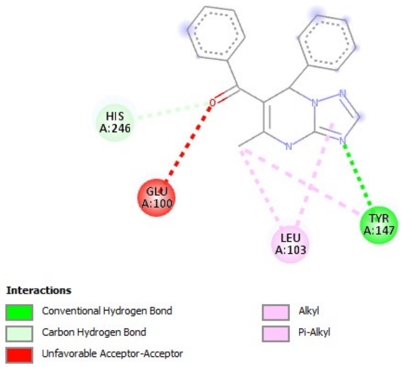	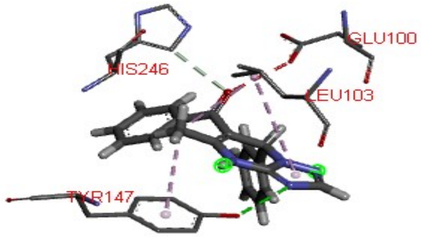
2.	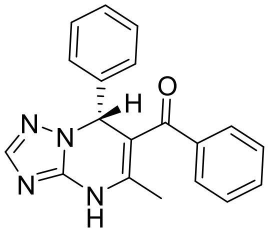 (*R*)-(5-methyl-7-phenyl-4,7-dihydro-[1,2,4]triazolo[1,5-*a*]pyrimidin-6-yl)(phenyl)methanone	-7.1	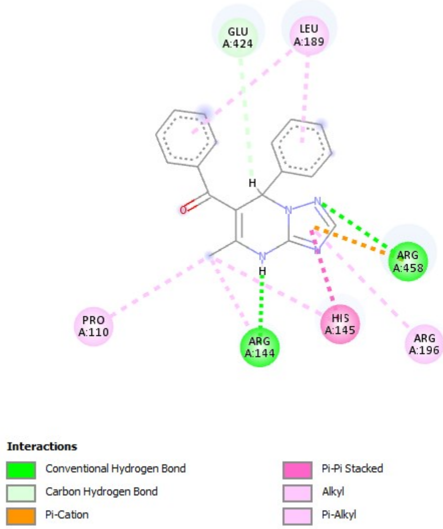	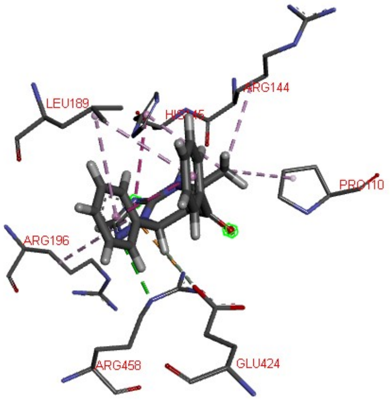

3.	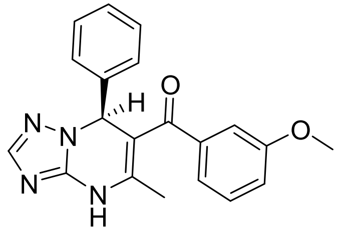 (*S*)-(3-methoxyphenyl)(5-methyl-7-phenyl-4,7-dihydro-[1,2,4]triazolo[1,5-*a*]pyrimidin-6-yl)methanone	-4.5	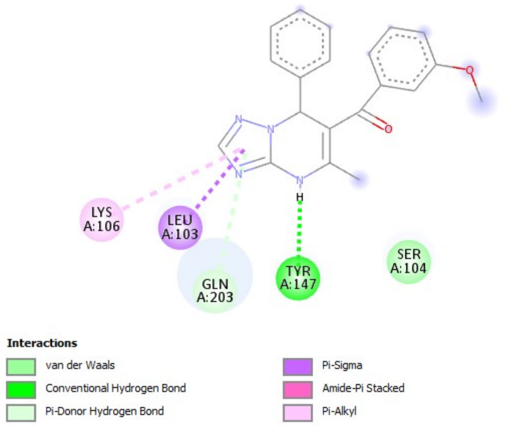	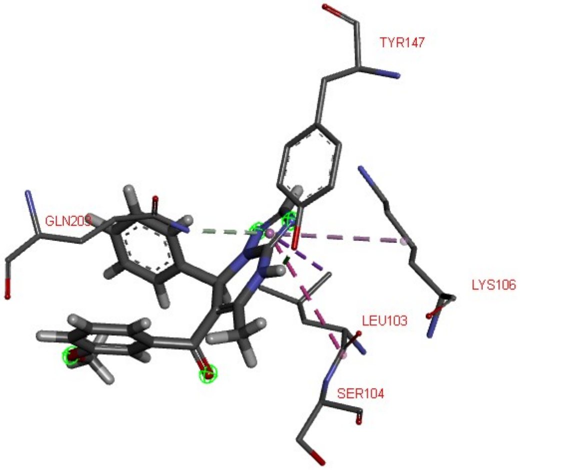
4.	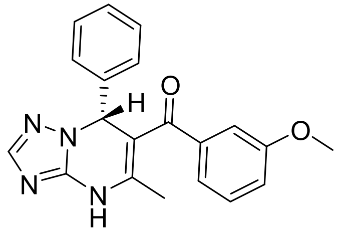 (*R*)-(3-methoxyphenyl)(5-methyl-7-phenyl-4,7-dihydro-[1,2,4]triazolo[1,5-*a*]pyrimidin-6-yl)methanone	-6.8	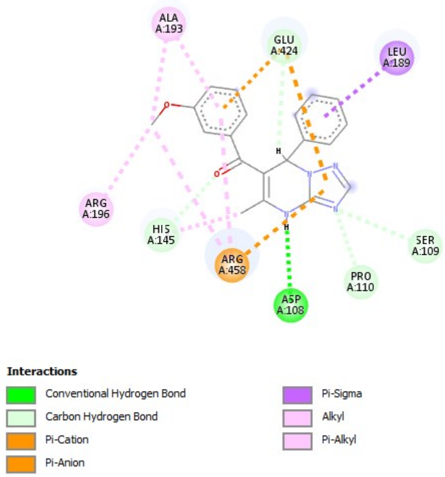	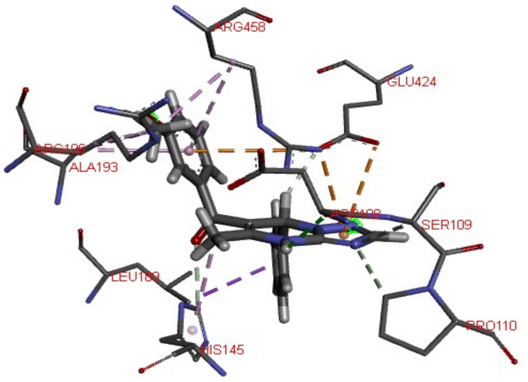

5.	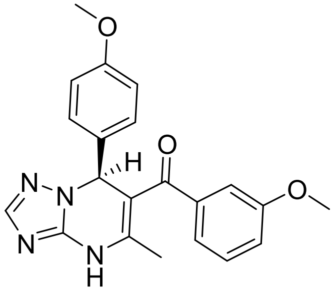 (*S*)-(3-methoxyphenyl)(7-(4-methoxyphenyl)-5-methyl-4,7-dihydro-[1,2,4]triazolo[1,5-*a*]pyrimidin-6-yl)methanone	-5.4	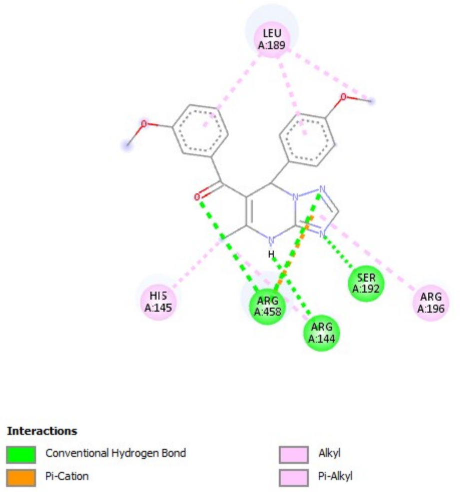	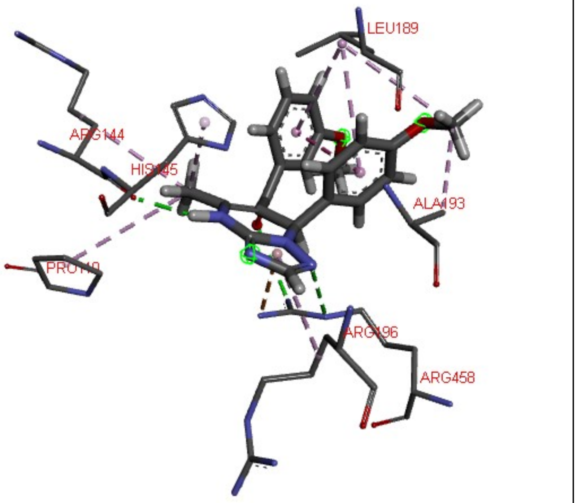
6.	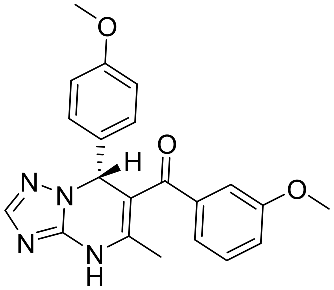 (*R*)-(3-methoxyphenyl)(7-(4-methoxyphenyl)-5-methyl-4,7-dihydro-[1,2,4]triazolo[1,5-*a*]pyrimidin-6-yl)methanone	-6.0	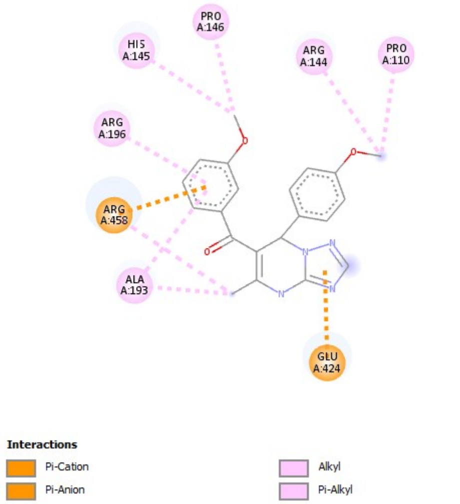	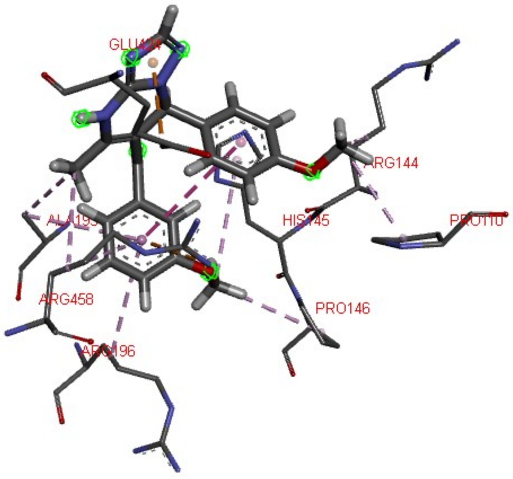

7.	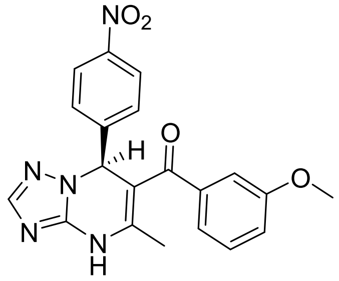<br>(*S*)-(3-methoxyphenyl)(5-methyl-7-(4-nitrophenyl)-4,7-dihydro-[1,2,4]triazolo[1,5-*a*]pyrimidin-6-yl)methanone	-5.1	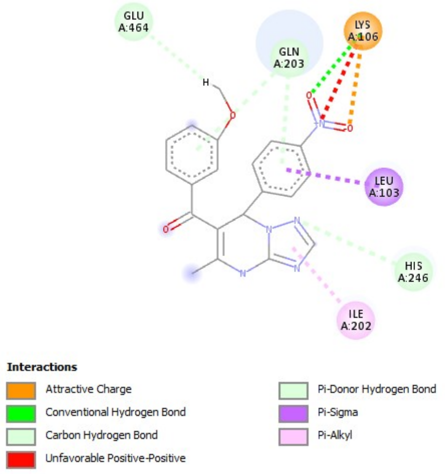	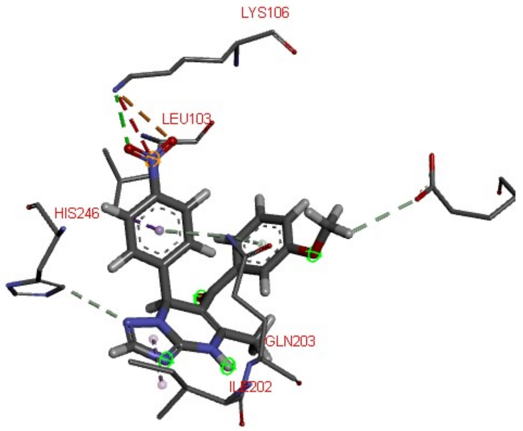
8.	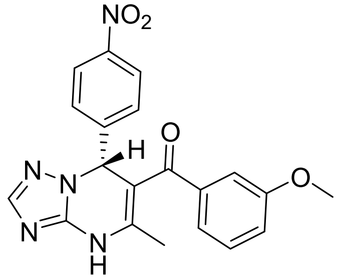<br>(*R*)-(3-methoxyphenyl)(5-methyl-7-(4-nitrophenyl)-4,7-dihydro-[1,2,4]triazolo[1,5-*a*]pyrimidin-6-yl)methanone	-5.8	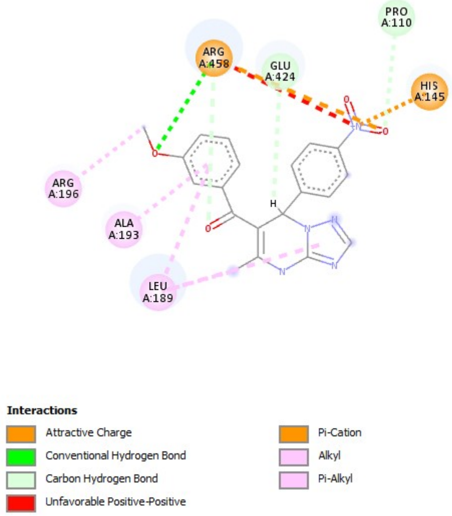	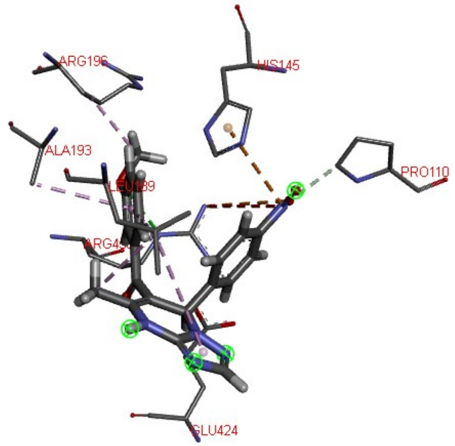

^{1}H NMR of (5-methyl-7-phenyl-4,7-dihydro-[1,2,4]triazolo[1,5-*a*]pyrimidin-6-yl)(phenyl)methanone (5a)

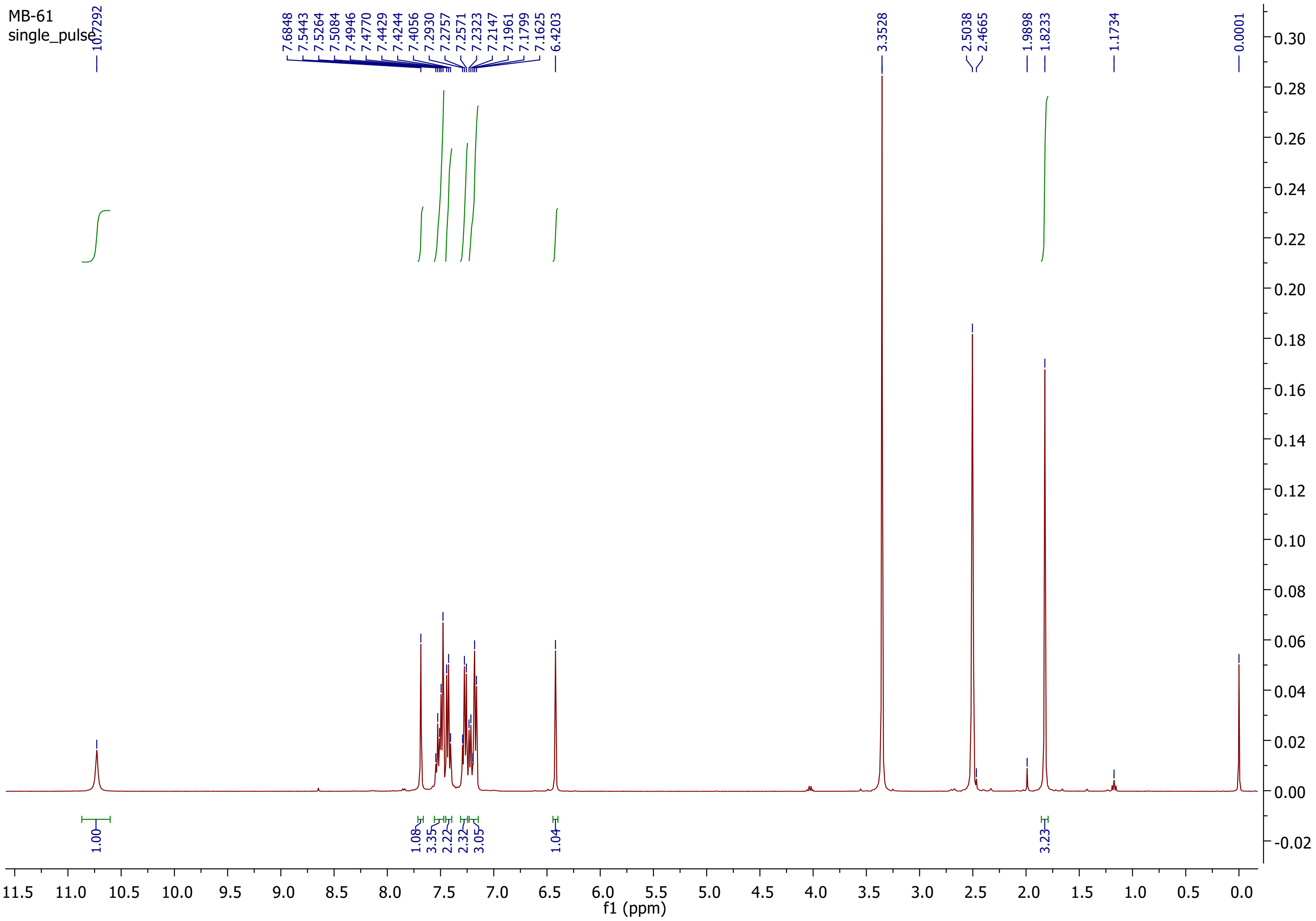

^{13}C NMR of (5-methyl-7-phenyl-4,7-dihydro-[1,2,4]triazolo[1,5-*a*]pyrimidin-6-yl)(phenyl)methanone (5a)

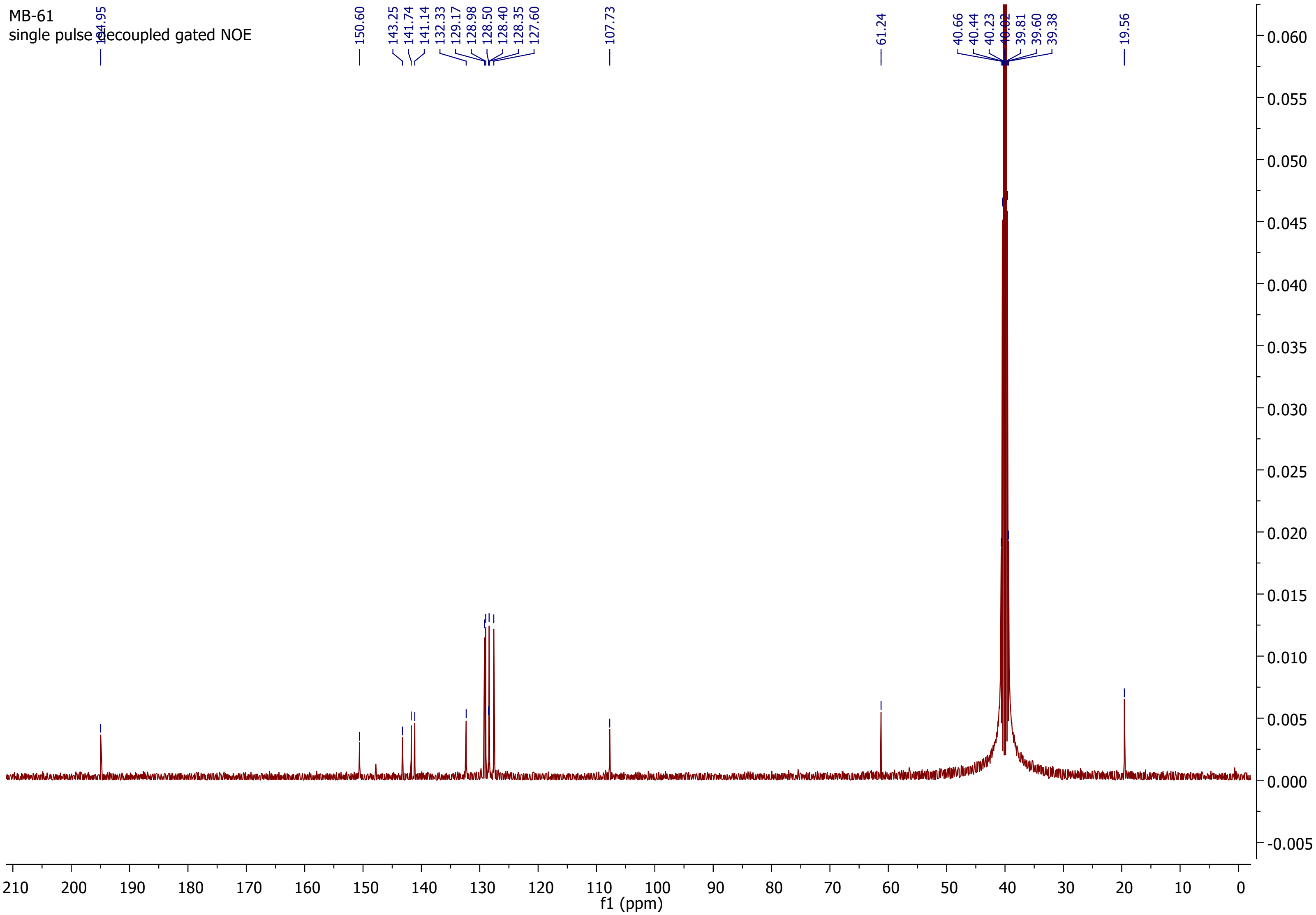

^{1}H NMR of (4"-Fluorophenyl)(5-methyl-7-phenyl-4,7-dihydro-[1,2,4]triazolo[1,5-*a*]pyrimidin-6-yl)methanone (5b)

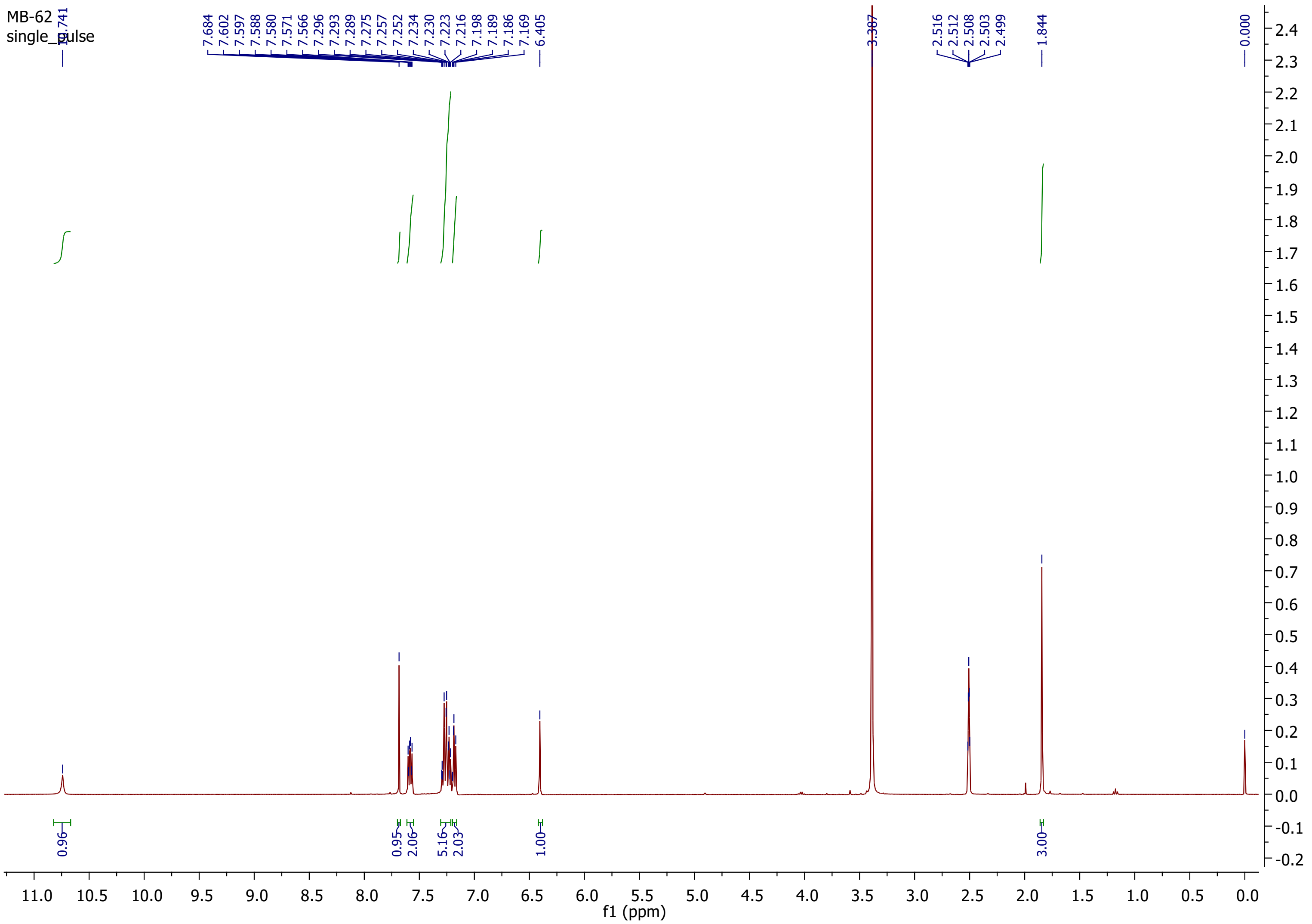

^{13}C NMR of (4''-Fluorophenyl)(5-methyl-7-phenyl-4,7-dihydro-[1,2,4]triazolo[1,5-*a*]pyrimidin-6-yl)methanone (5b)

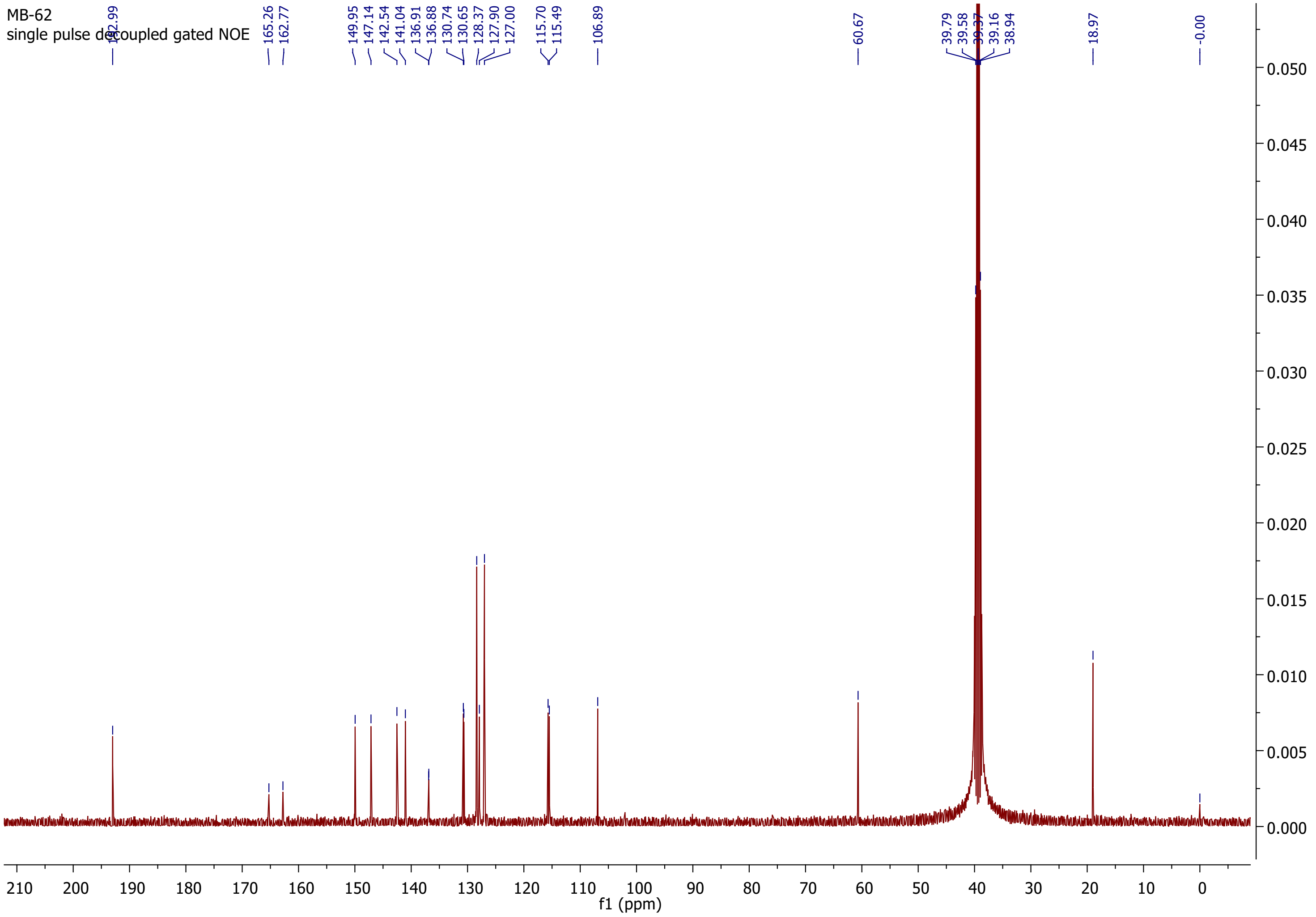

^{1}H NMR of (4''-Chlorophenyl)(5-methyl-7-phenyl-4,7-dihydro-[1,2,4]triazolo[1,5-*a*]pyrimidin-6-yl)methanone (5c)

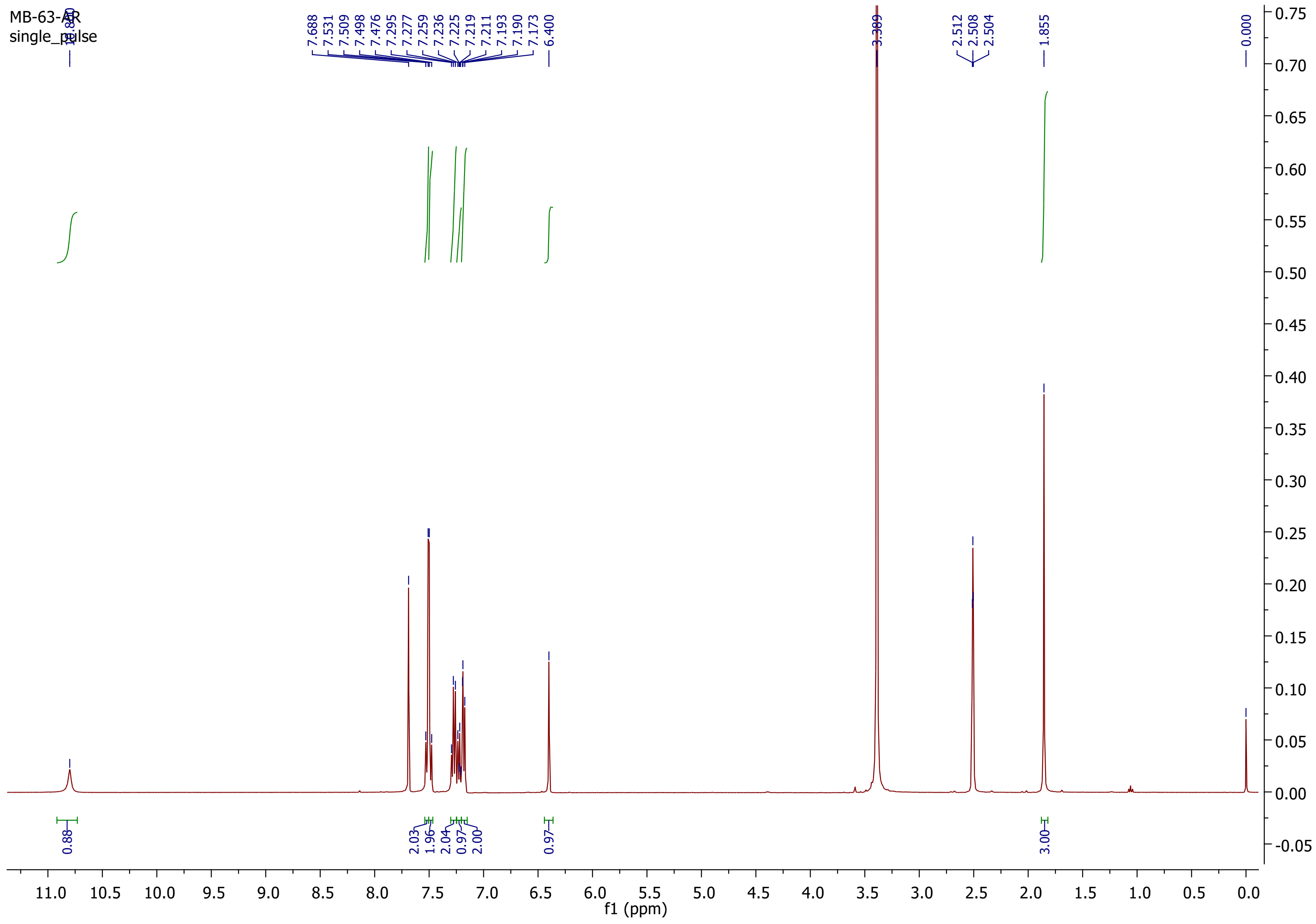

^{13}C NMR of (4''-Chlorophenyl)(5-methyl-7-phenyl-4,7-dihydro-[1,2,4]triazolo[1,5-*a*]pyrimidin-6-yl)methanone (5c)

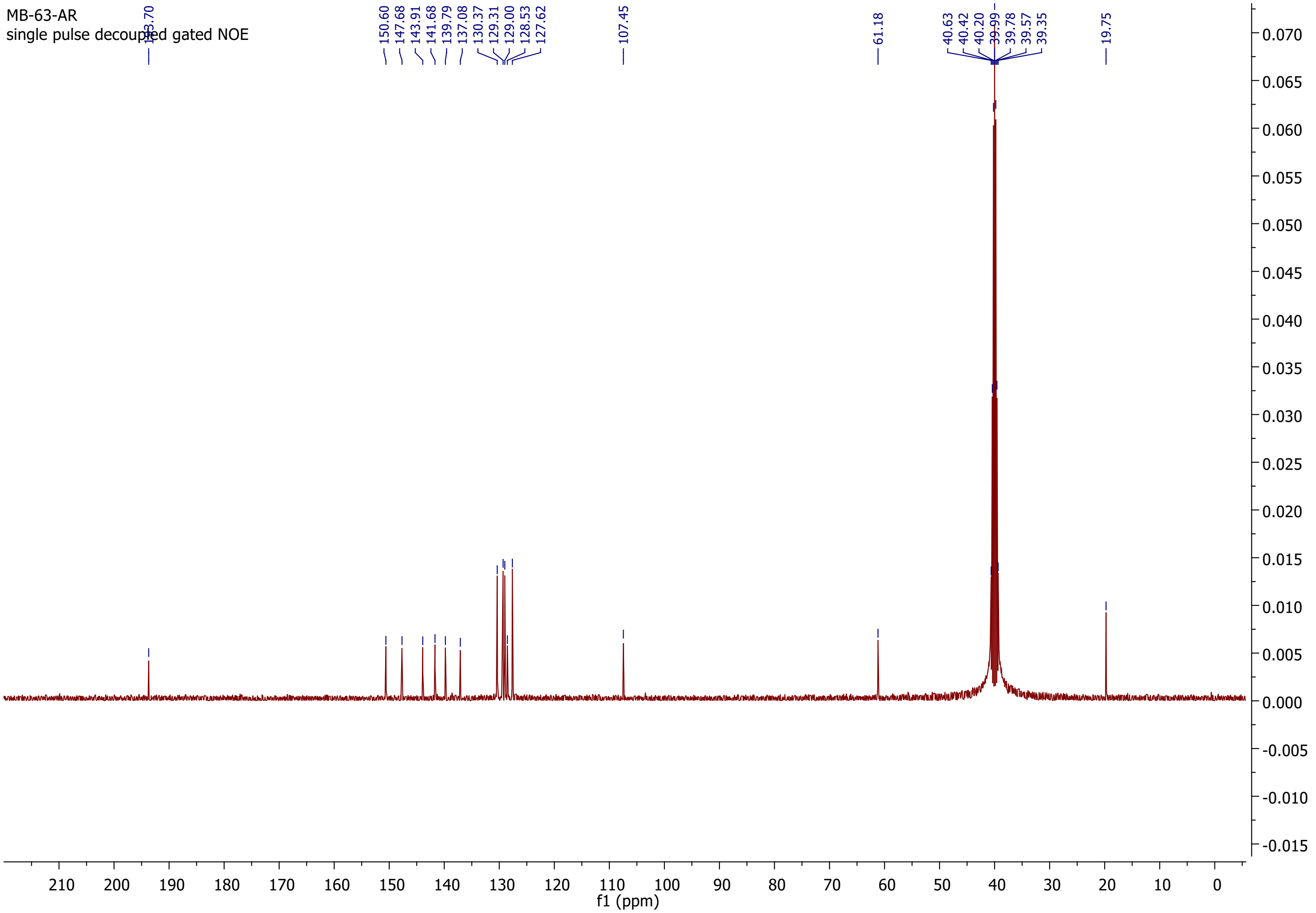

HRMS of (4''-Chlorophenyl)(5-methyl-7-phenyl-4,7-dihydro-[1,2,4]triazolo[1,5-*a*]pyrimidin-6-yl)methanone (5c)

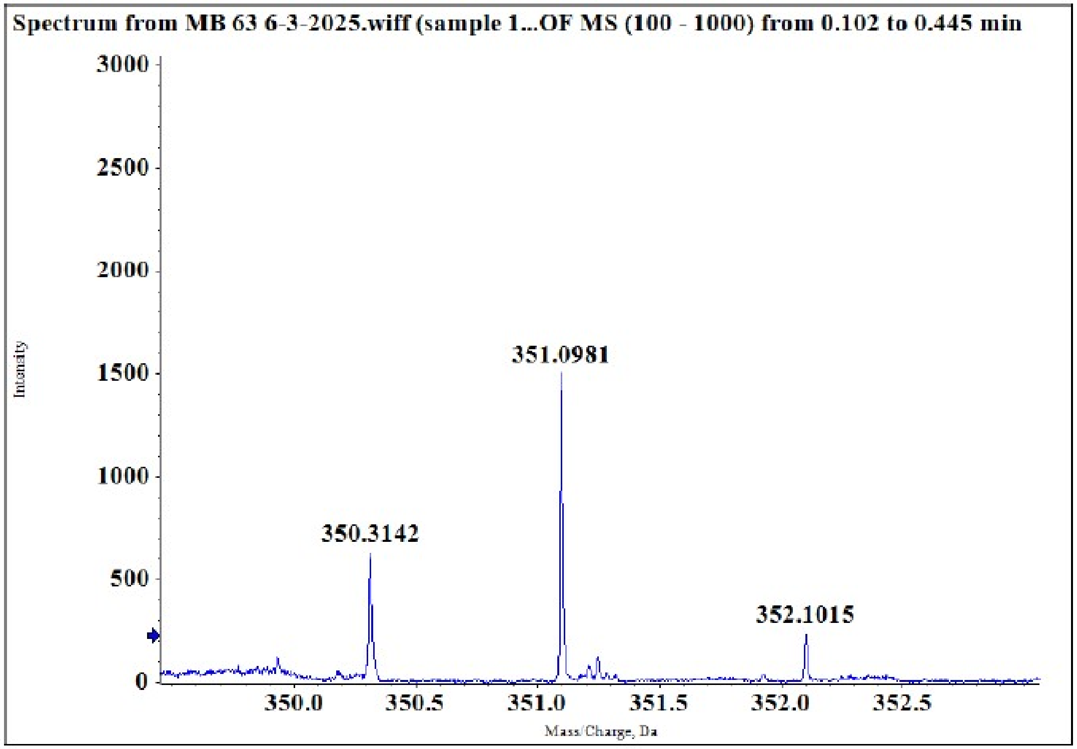

3/6/2025 3:56:34 PM

^{1}H NMR of (4''-Bromophenyl)(5-methyl-7-phenyl-4,7-dihydro-[1,2,4]triazolo[1,5-*a*]pyrimidin-6-yl)methanone (5d)

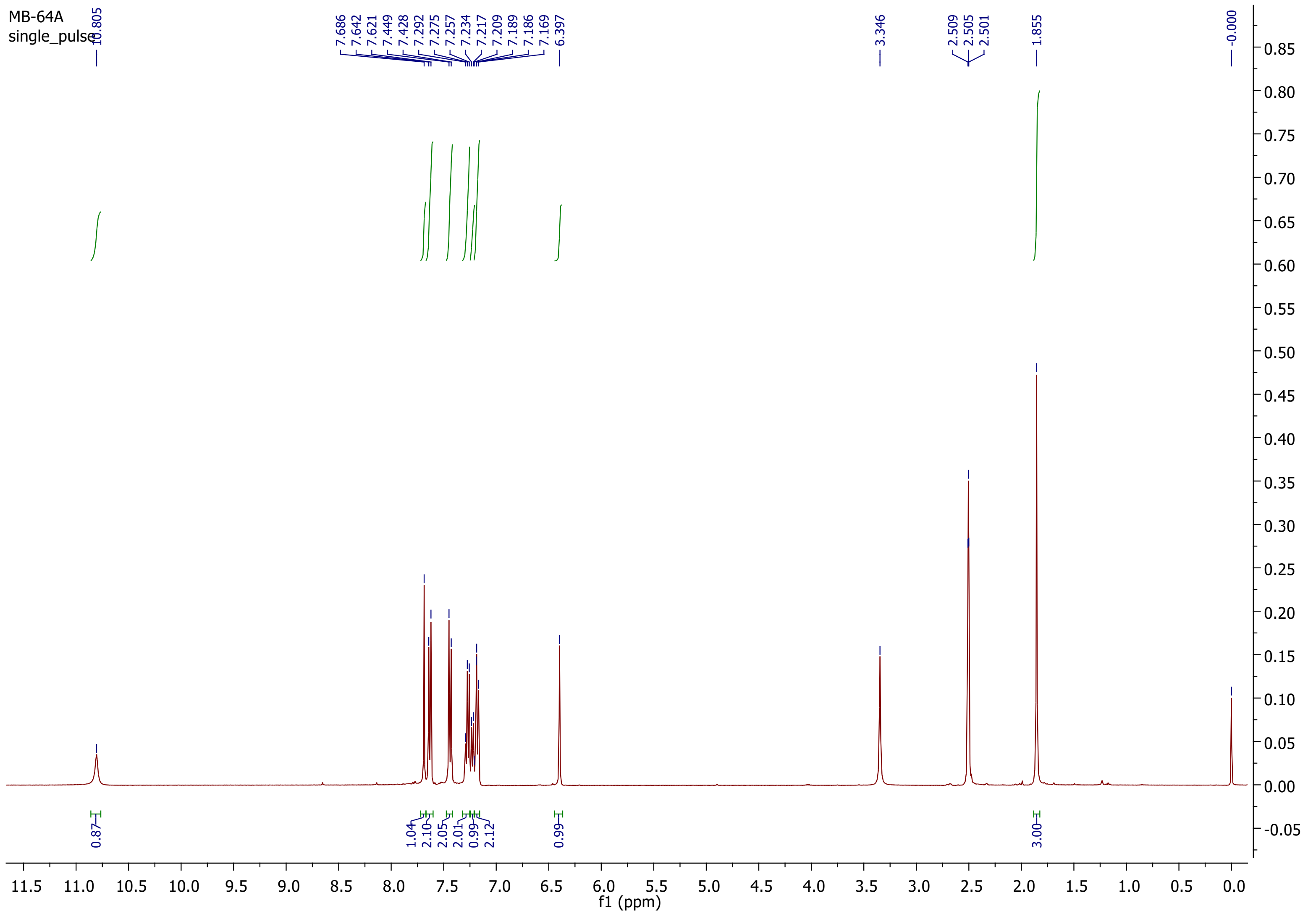

^{13}C NMR of (4''-Bromophenyl)(5-methyl-7-phenyl-4,7-dihydro-[1,2,4]triazolo[1,5-*a*]pyrimidin-6-yl)methanone (5d)

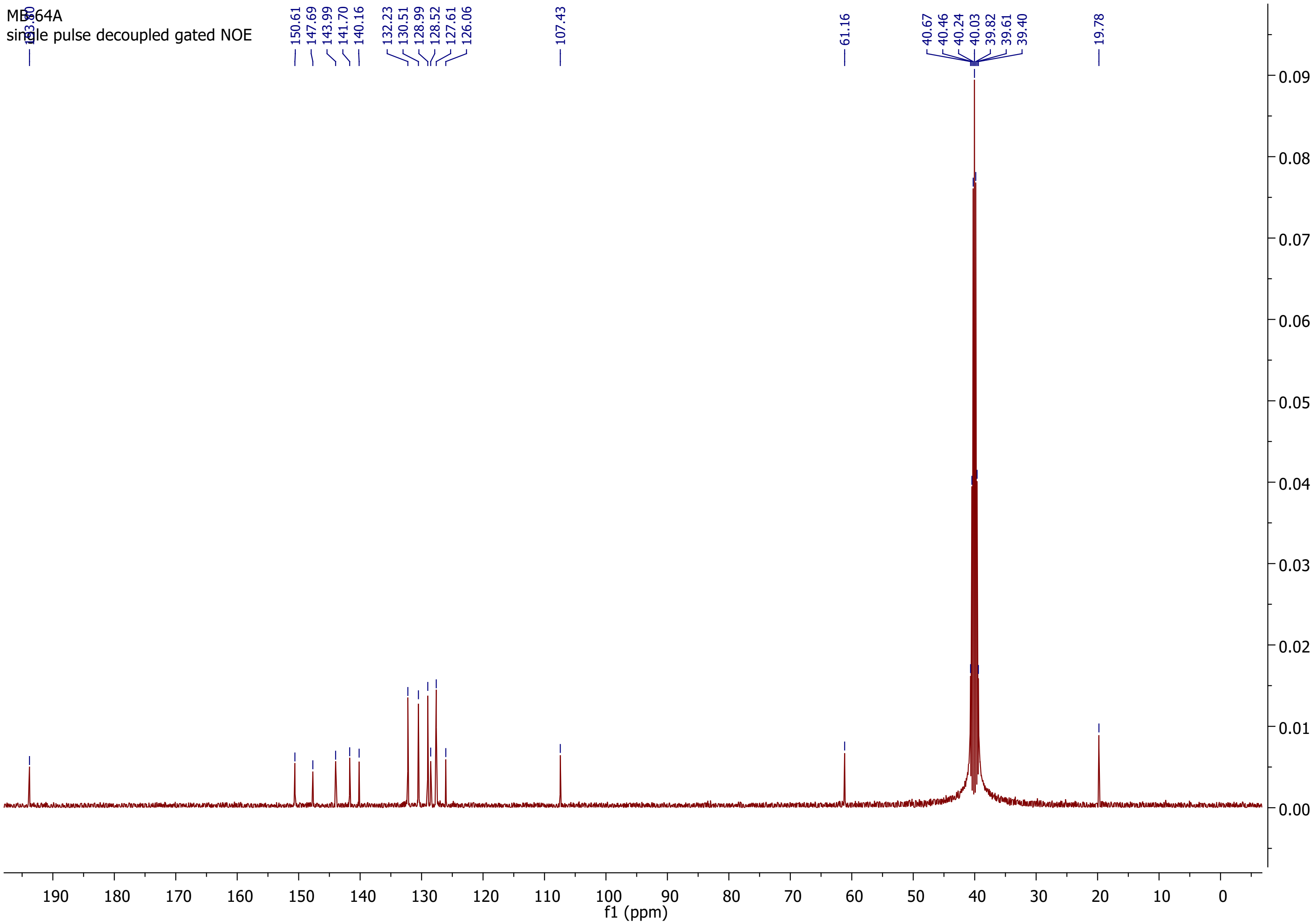

^{1}H NMR of (4''-Methoxyphenyl)(5-methyl-7-phenyl-4,7-dihydro-[1,2,4]triazolo[1,5-*a*]pyrimidin-6-yl)methanone (5e)

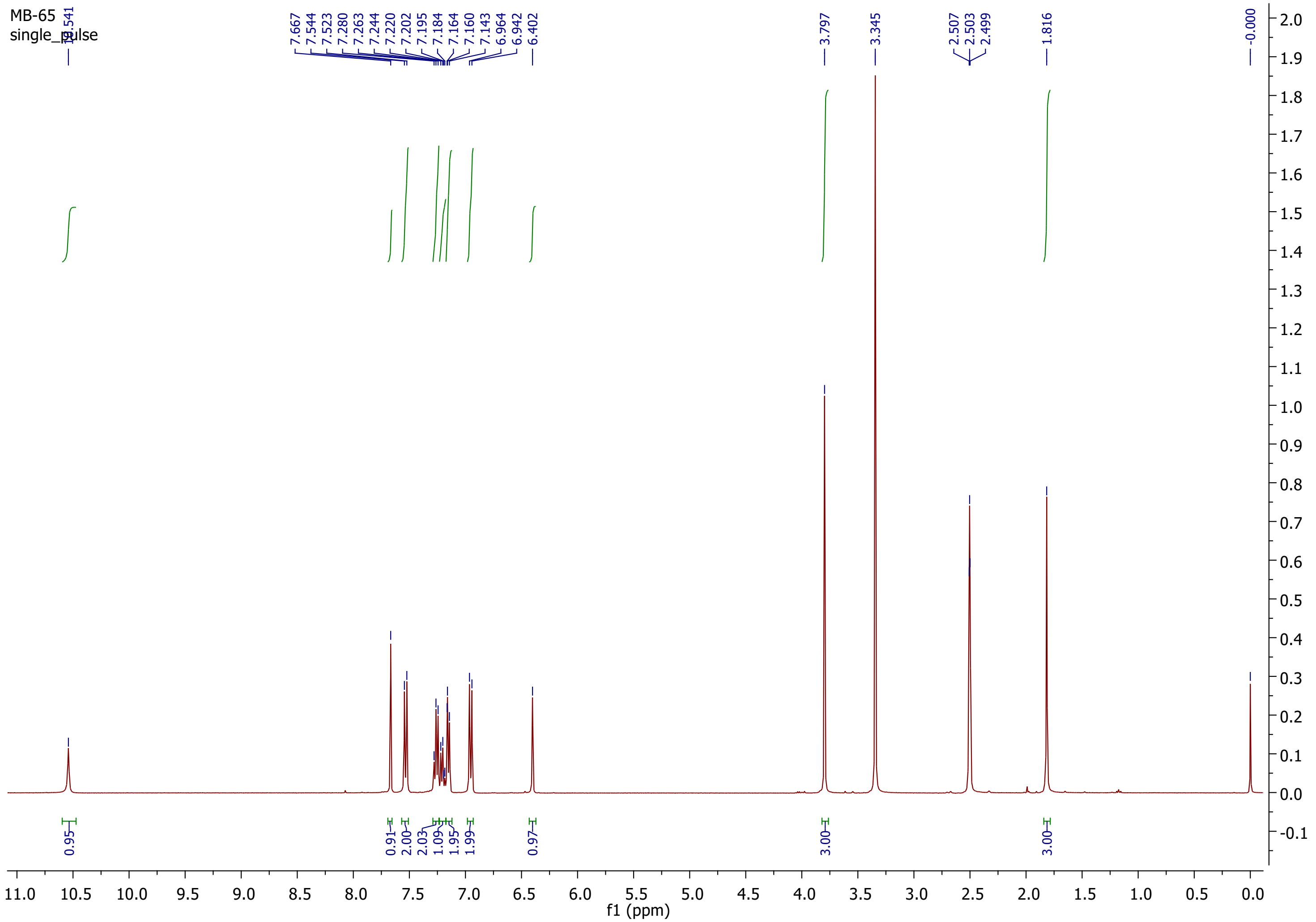

^{13}C NMR of (4''-Methoxyphenyl)(5-methyl-7-phenyl-4,7-dihydro-[1,2,4]triazolo[1,5-*a*]pyrimidin-6-yl)methanone (5e)

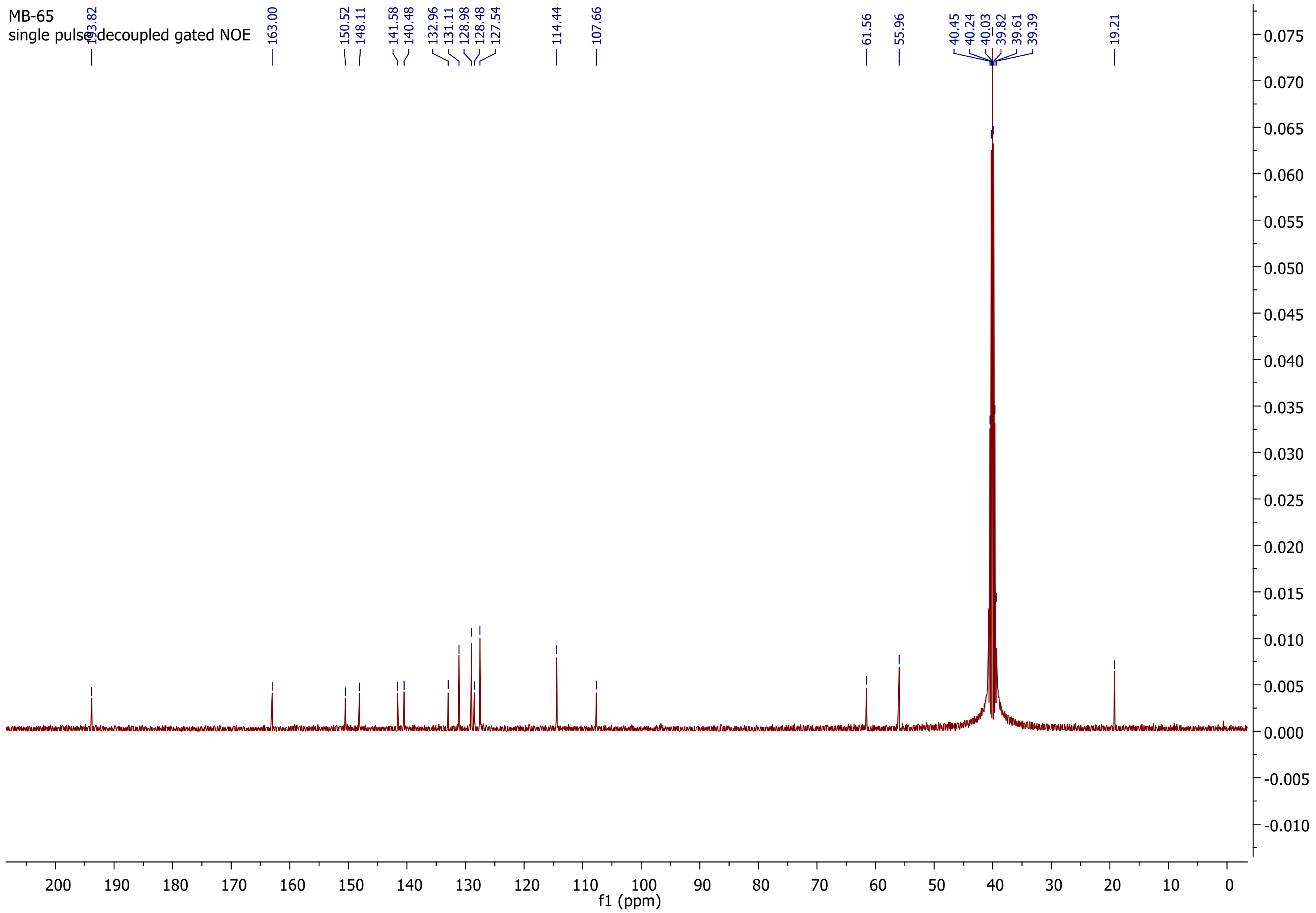

^{1}H NMR of (3''-Methoxyphenyl)(5-methyl-7-phenyl-4,7-dihydro-[1,2,4]triazolo[1,5-*a*]pyrimidin-6-yl)methanone (5f)

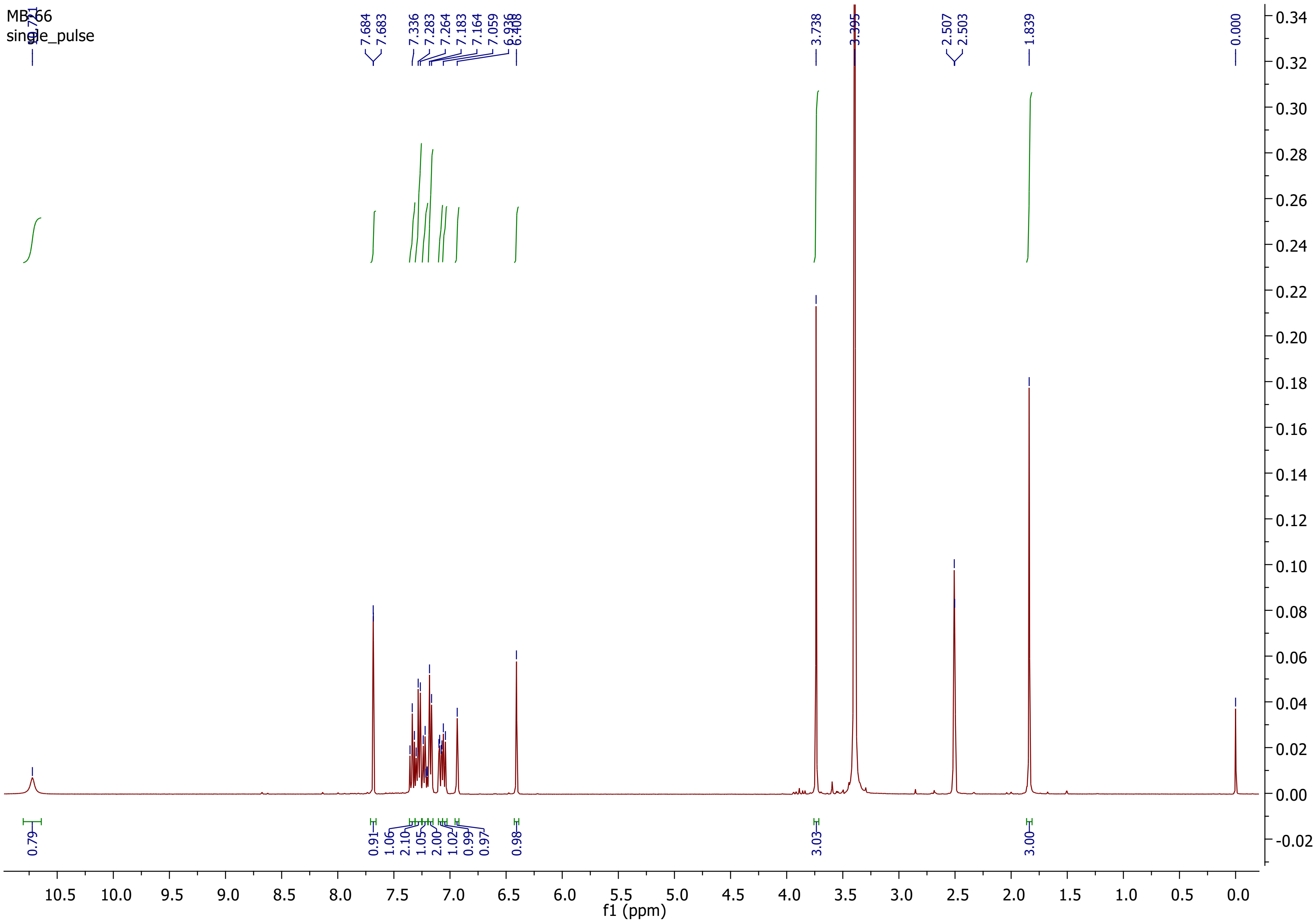

^{13}C NMR of (3''-Methoxyphenyl)(5-methyl-7-phenyl-4,7-dihydro-[1,2,4]triazolo[1,5-*a*]pyrimidin-6-yl)methanone (5f)

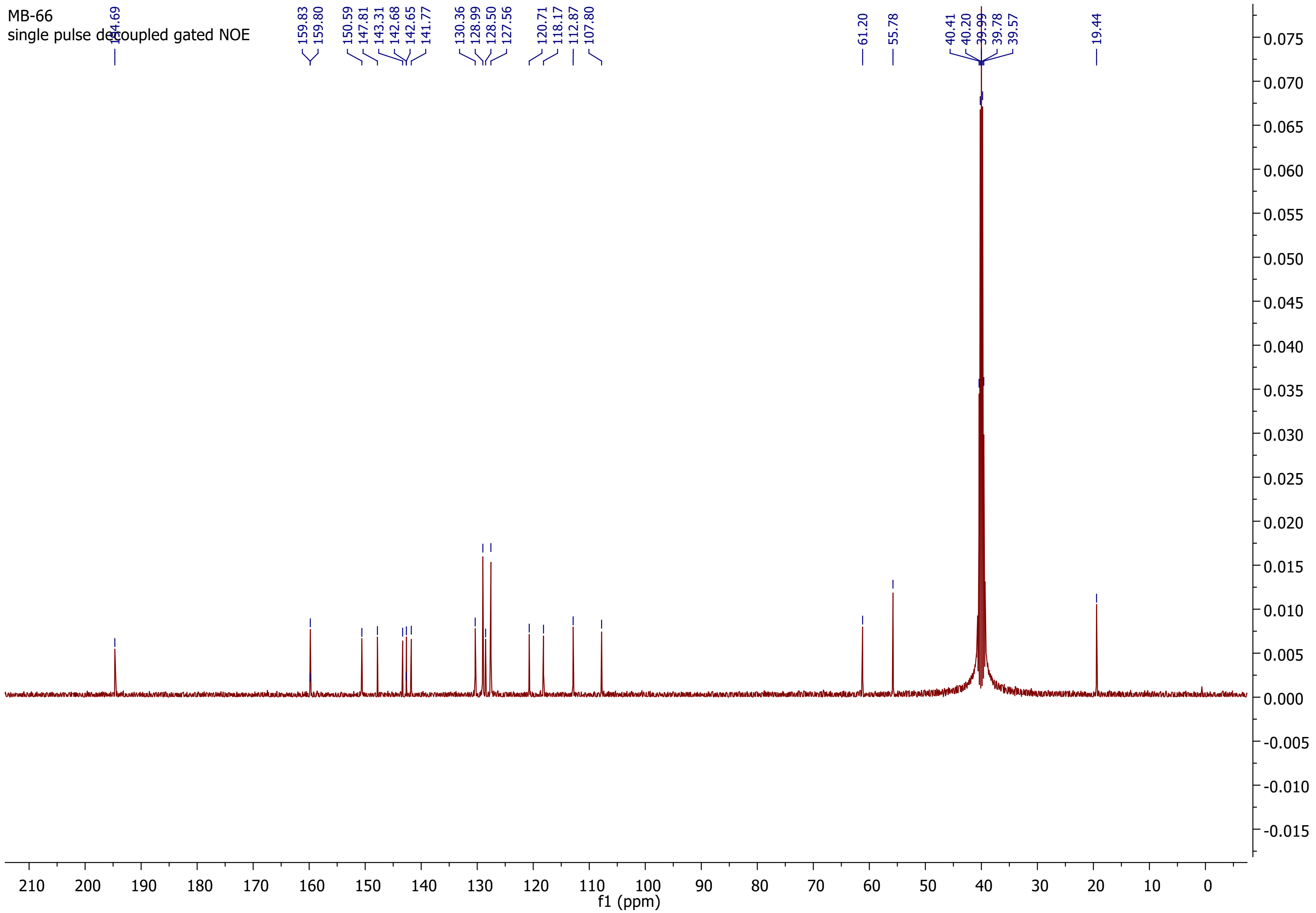

HRMS of (3''-Methoxyphenyl)(5-methyl-7-phenyl-4,7-dihydro-[1,2,4]triazolo[1,5-*a*]pyrimidin-6-yl)methanone (5f)

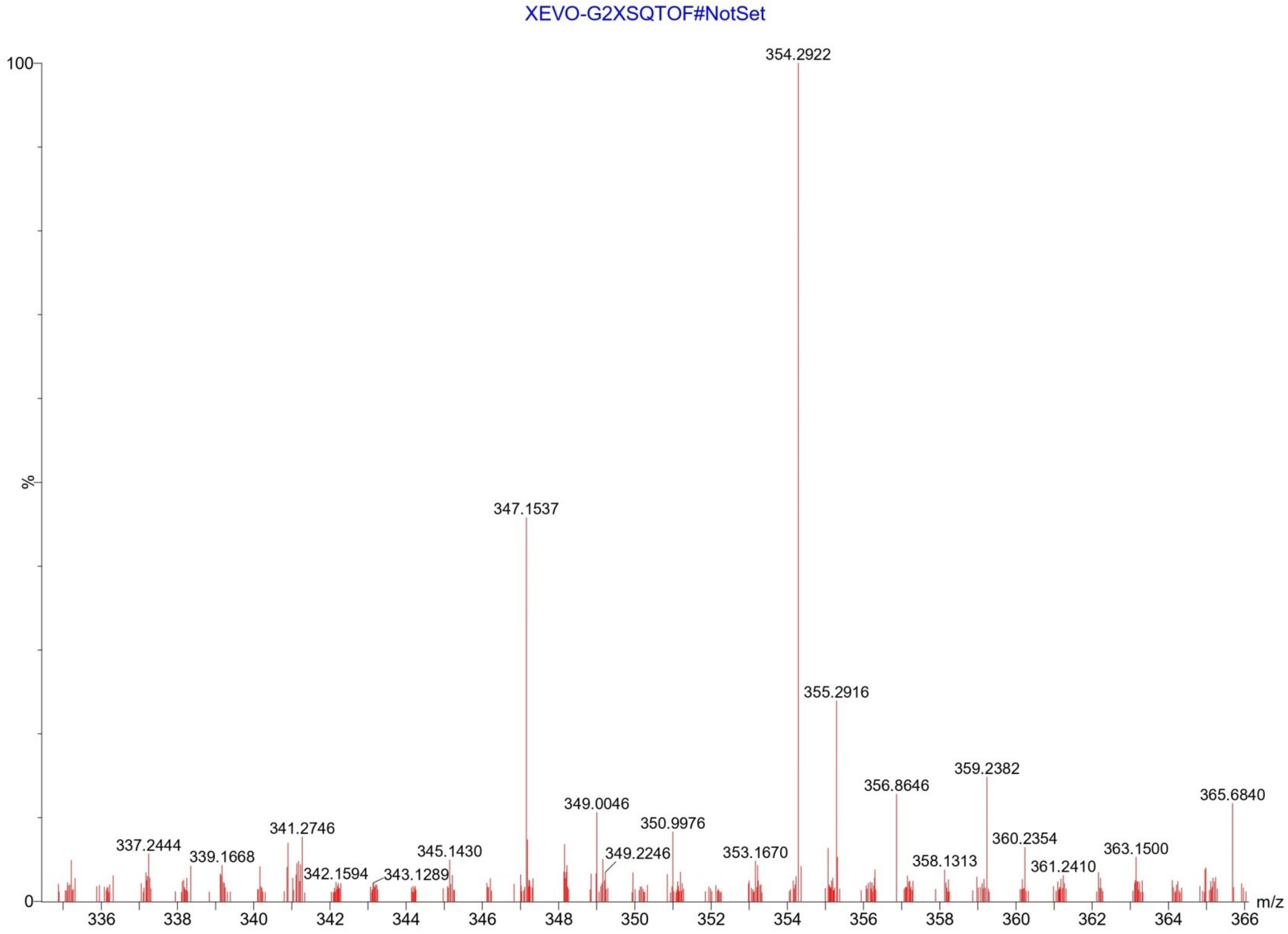

^{1}H NMR of (5-Methyl-7-phenyl-4,7-dihydro-[1,2,4]triazolo[1,5-*a*]pyrimidin-6-yl)(thiophen-2''-yl)methanone (5g)

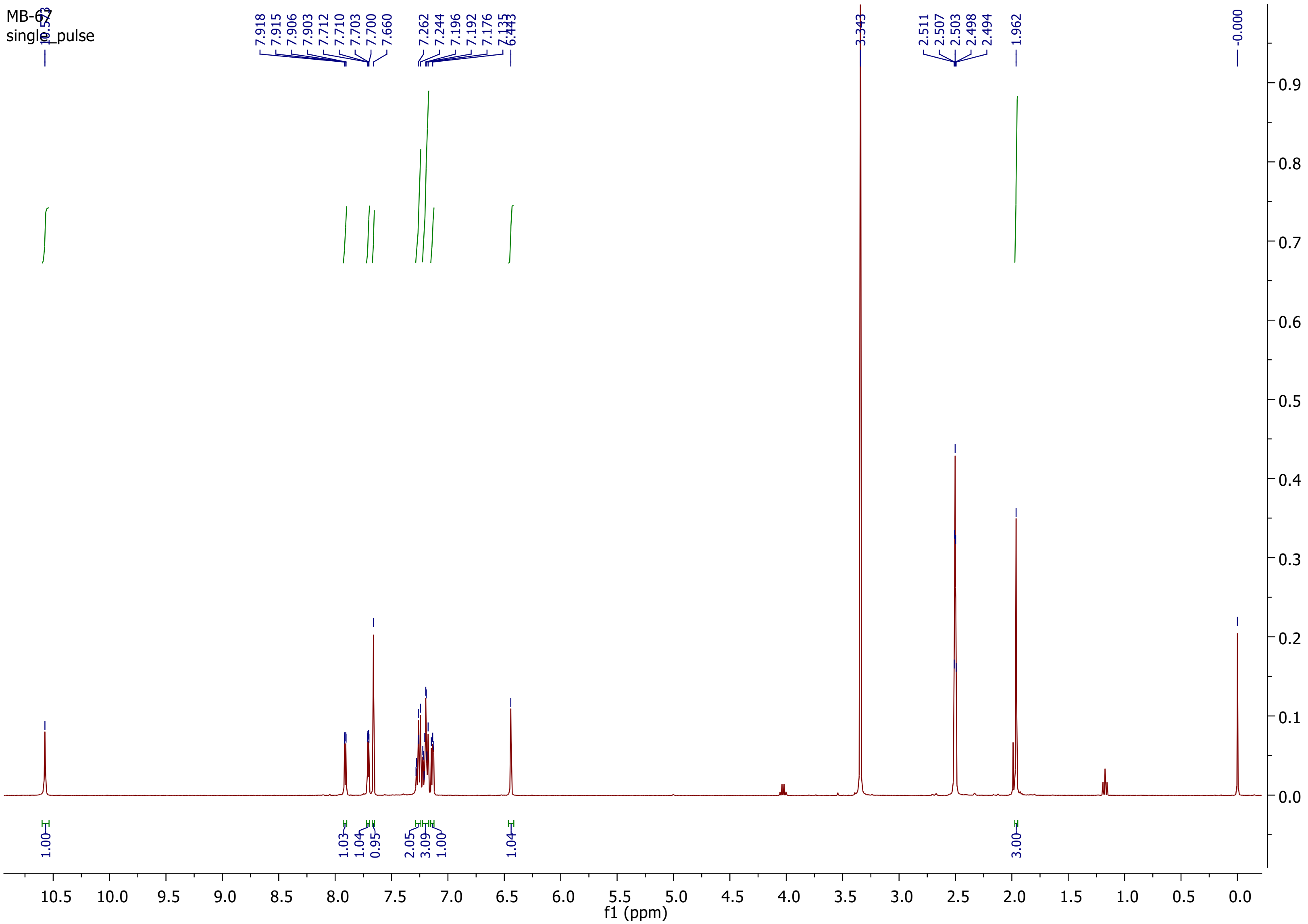

^{13}C NMR of (5-Methyl-7-phenyl-4,7-dihydro-[1,2,4]triazolo[1,5-*a*]pyrimidin-6-yl)(thiophen-2''-yl)methanone (5g)

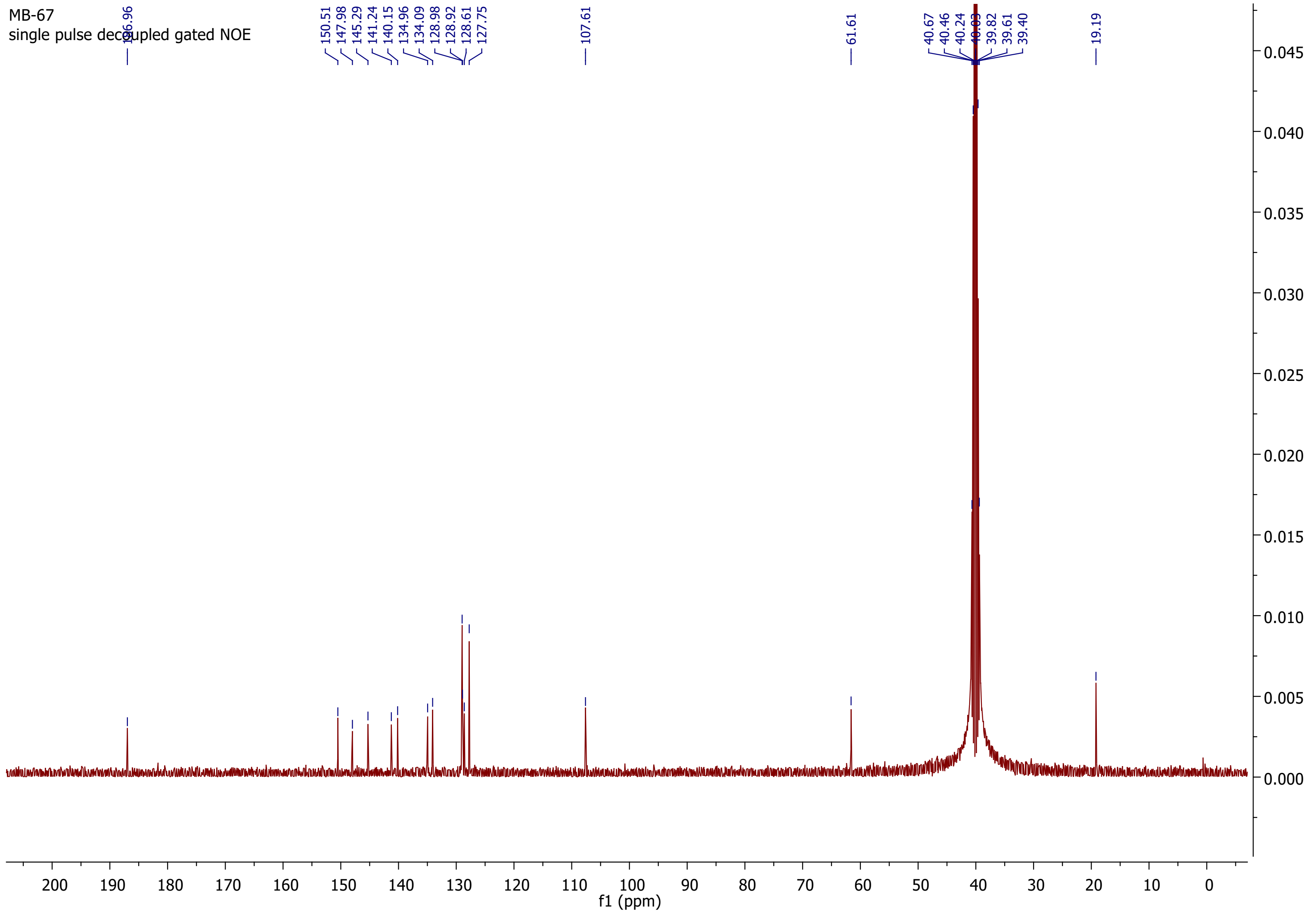

^{1}H NMR of (7-(4'-Methoxyphenyl)-5-methyl-4,7-dihydro-[1,2,4]triazolo[1,5-*a*]pyrimidin-6-yl) (phenyl)methanone (5h)

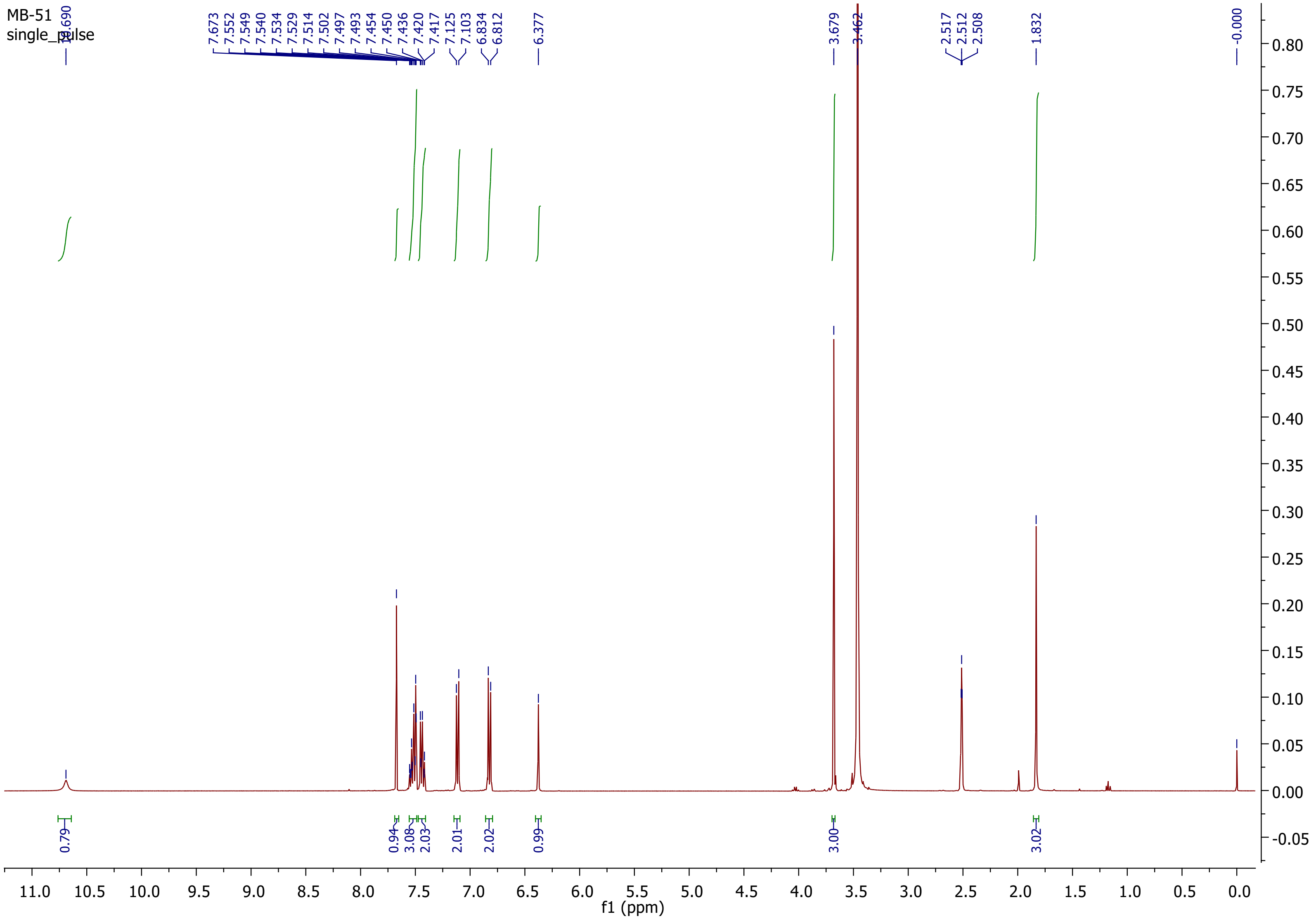

^{13}C NMR of (7-(4'-Methoxyphenyl)-5-methyl-4,7-dihydro-[1,2,4]triazolo[1,5-*a*]pyrimidin-6-yl) (phenyl)methanone (5h)

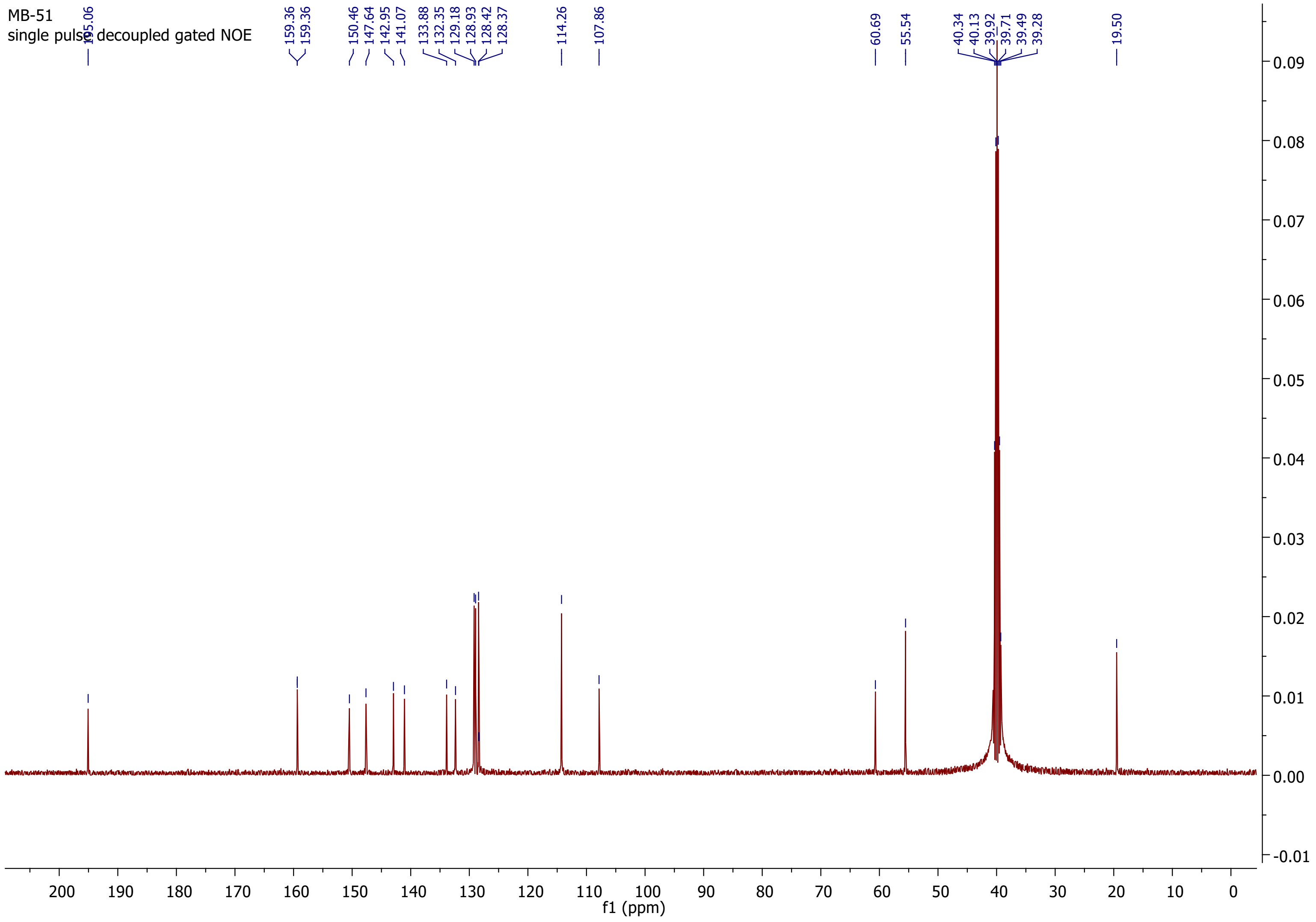

^{1}H NMR of (4''-Fluorophenyl)(7-(4'-methoxyphenyl)-5-methyl-4,7-dihydro-[1,2,4]triazolo[1,5-*a*]pyrimidin-6-yl)methanone (5i)

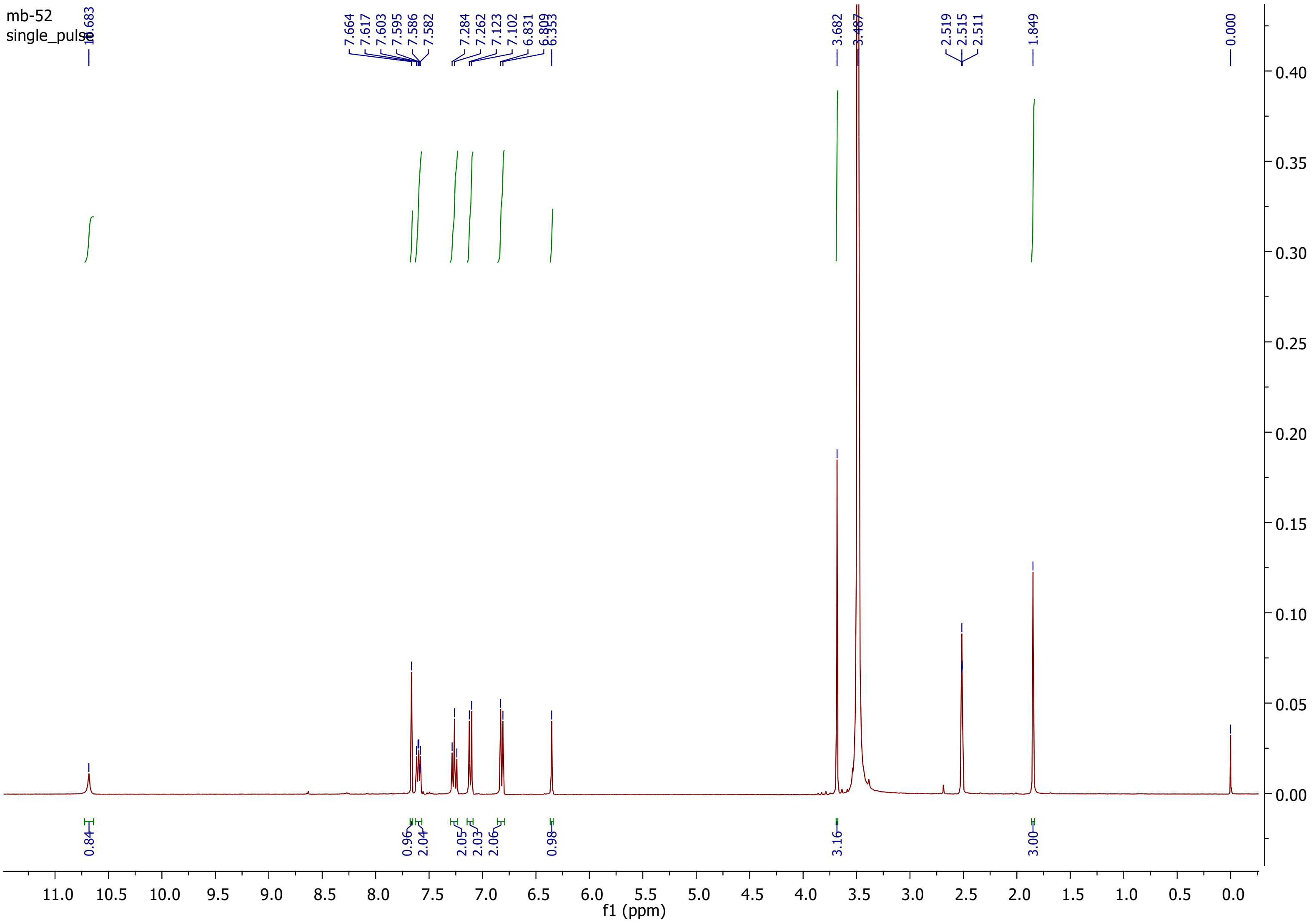

^{13}C NMR of (4''-Fluorophenyl)(7-(4'-methoxyphenyl)-5-methyl-4,7-dihydro-[1,2,4]triazolo[1,5-*a*]pyrimidin-6-yl)methanone (5i)

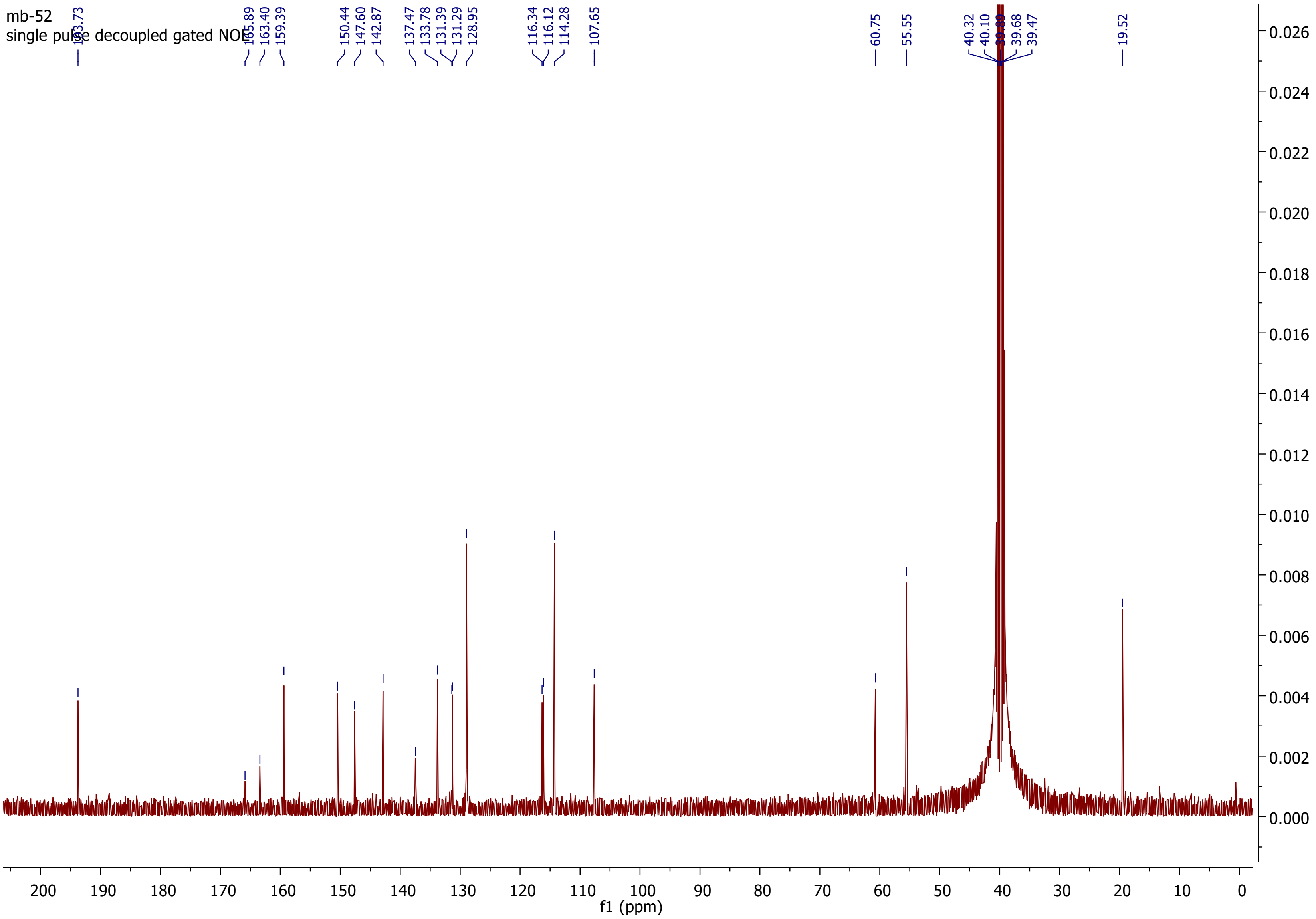

^{1}H NMR of (4''-Chlorophenyl)(7-(4'-methoxyphenyl)-5-methyl-4,7-dihydro-[1,2,4]triazolo[1,5-*a*]pyrimidin-6-yl)methanone (5j)

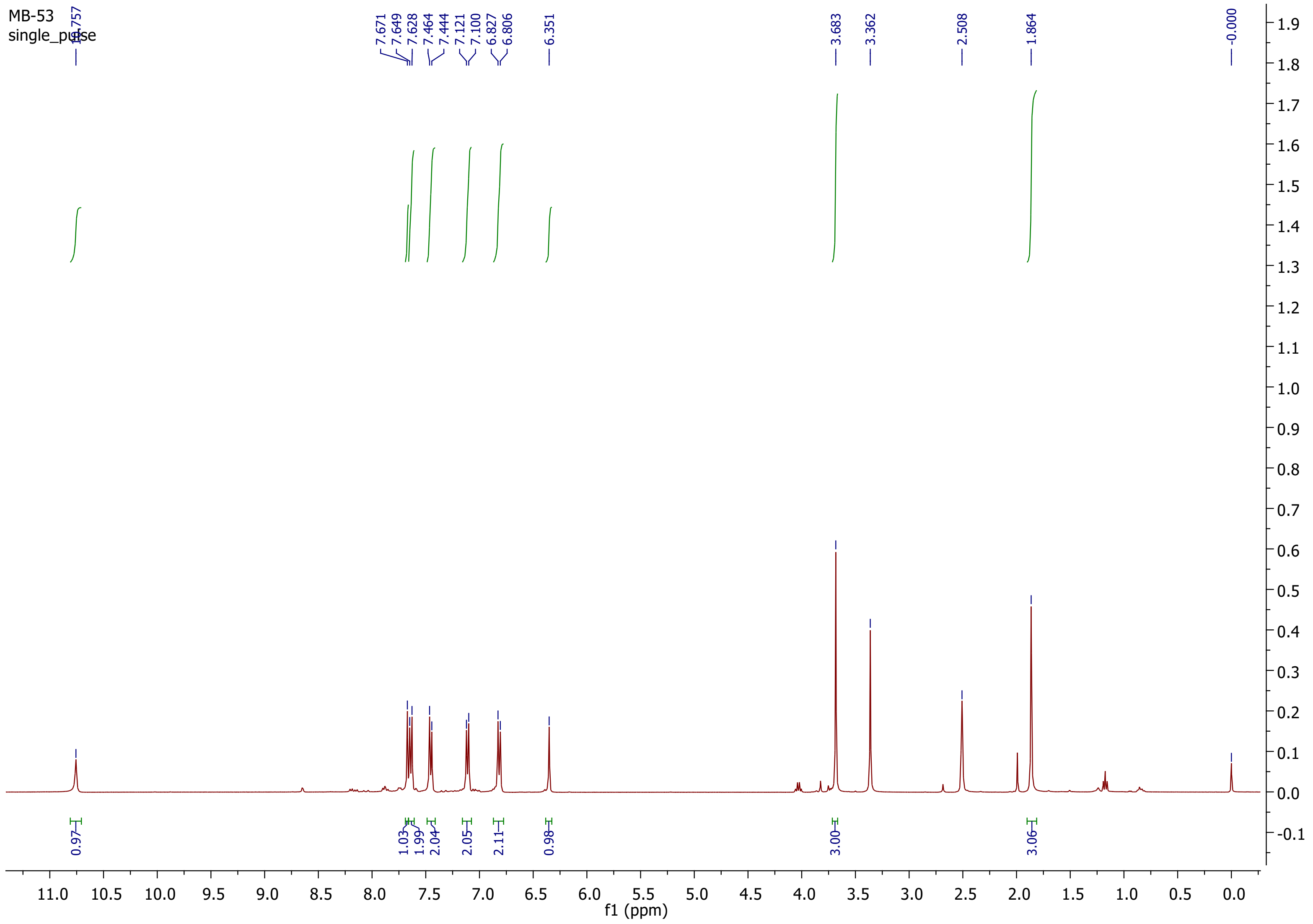

^{13}C NMR of (4''-Chlorophenyl)(7-(4'-methoxyphenyl)-5-methyl-4,7-dihydro-[1,2,4]triazolo[1,5-*a*]pyrimidin-6-yl)methanone (5j)

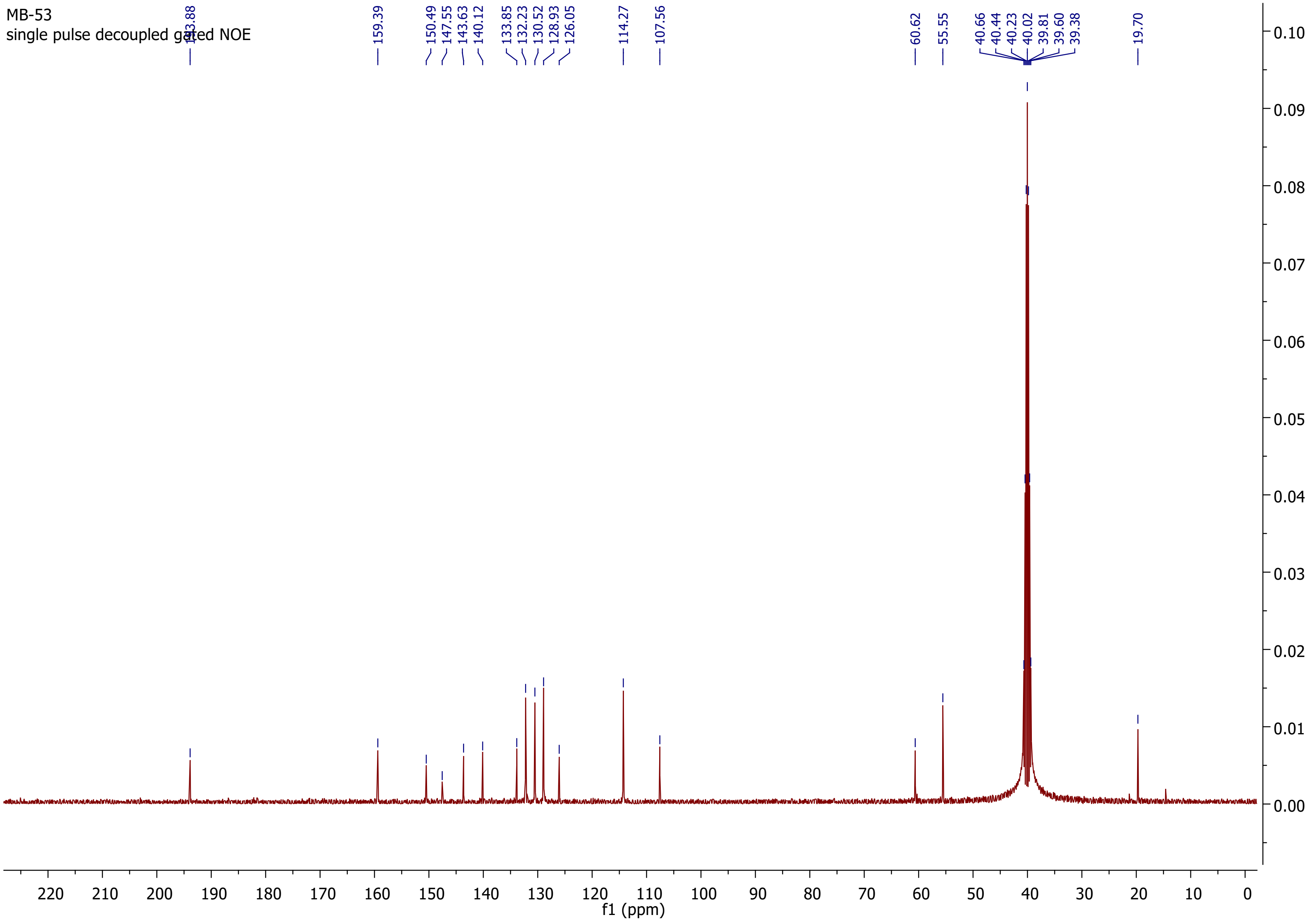

HRMS of (4''-Chlorophenyl)(7-(4'-methoxyphenyl)-5-methyl-4,7-dihydro-[1,2,4]triazolo[1,5-*a*]pyrimidin-6-yl)methanone (5j)

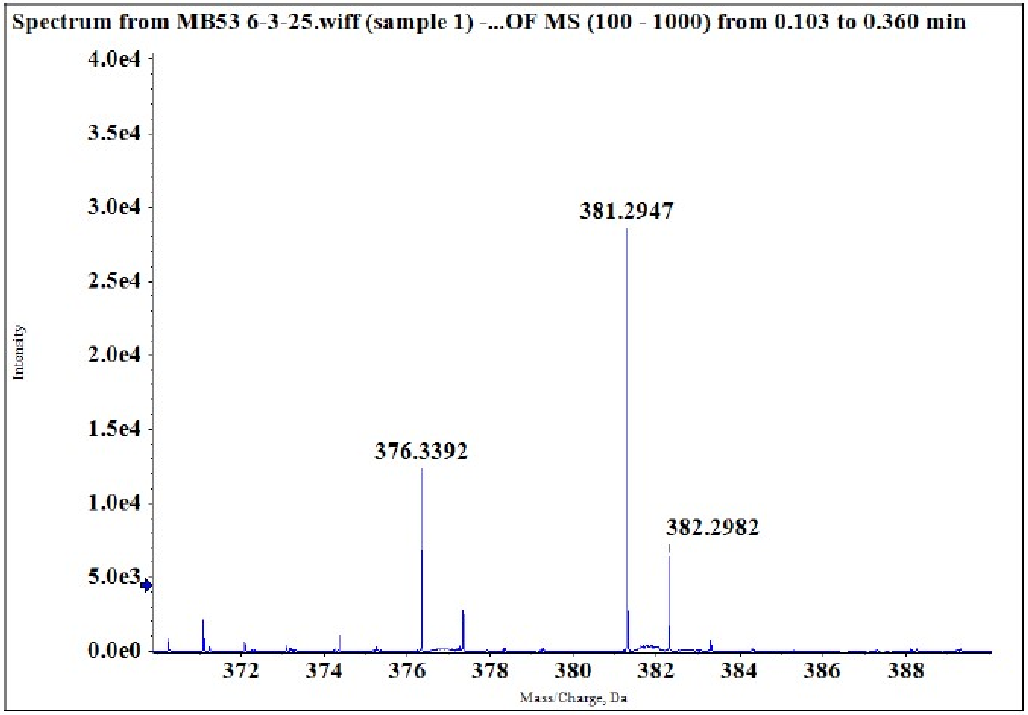

3/6/2025 3:54:50 PM

^{1}H NMR of (4''-Bromophenyl)(7-(4'-methoxyphenyl)-5-methyl-4,7-dihydro-[1,2,4]triazolo[1,5-*a*]pyrimidin-6-yl)methanone (5k)

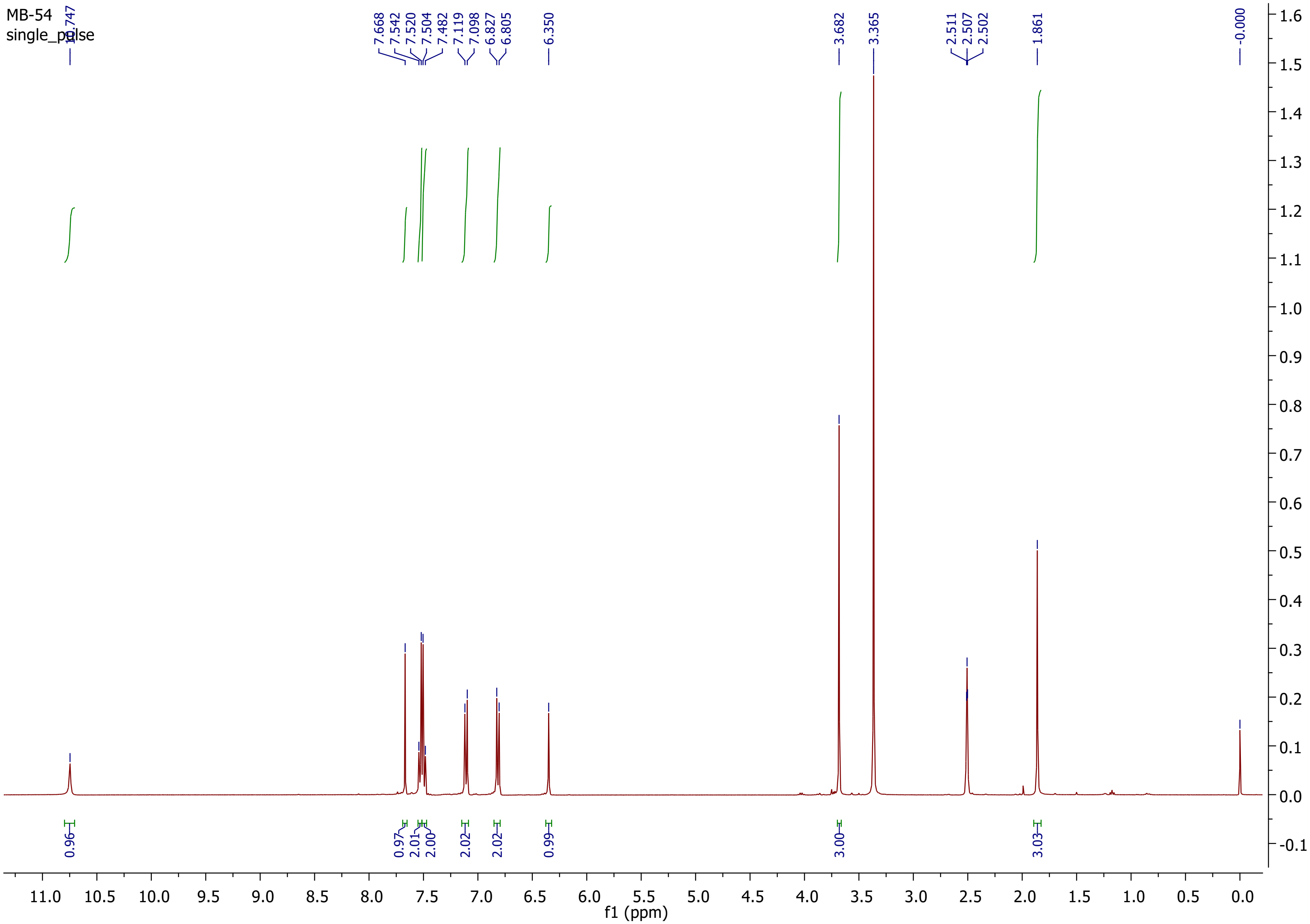

^{13}C NMR of (4''-Bromophenyl)(7-(4'-methoxyphenyl)-5-methyl-4,7-dihydro-[1,2,4]triazolo[1,5-*a*]pyrimidin-6-yl)methanone (5k)

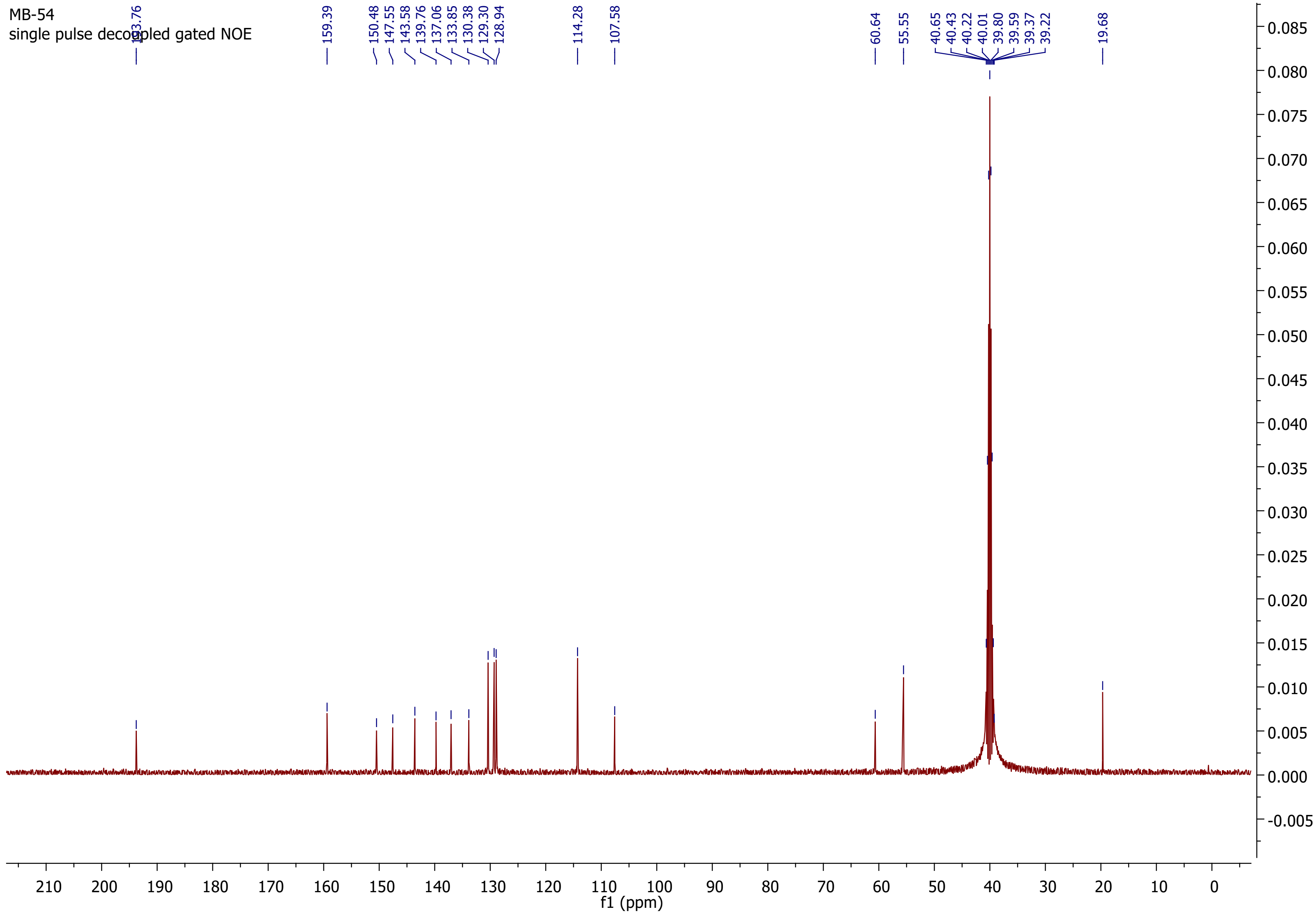

^{1}H NMR of (4''-Methoxyphenyl)(7-(4'-methoxyphenyl)-5-methyl-4,7-dihydro-[1,2,4]triazolo[1,5-*a*]pyrimidin-6-yl)methanone (5l)

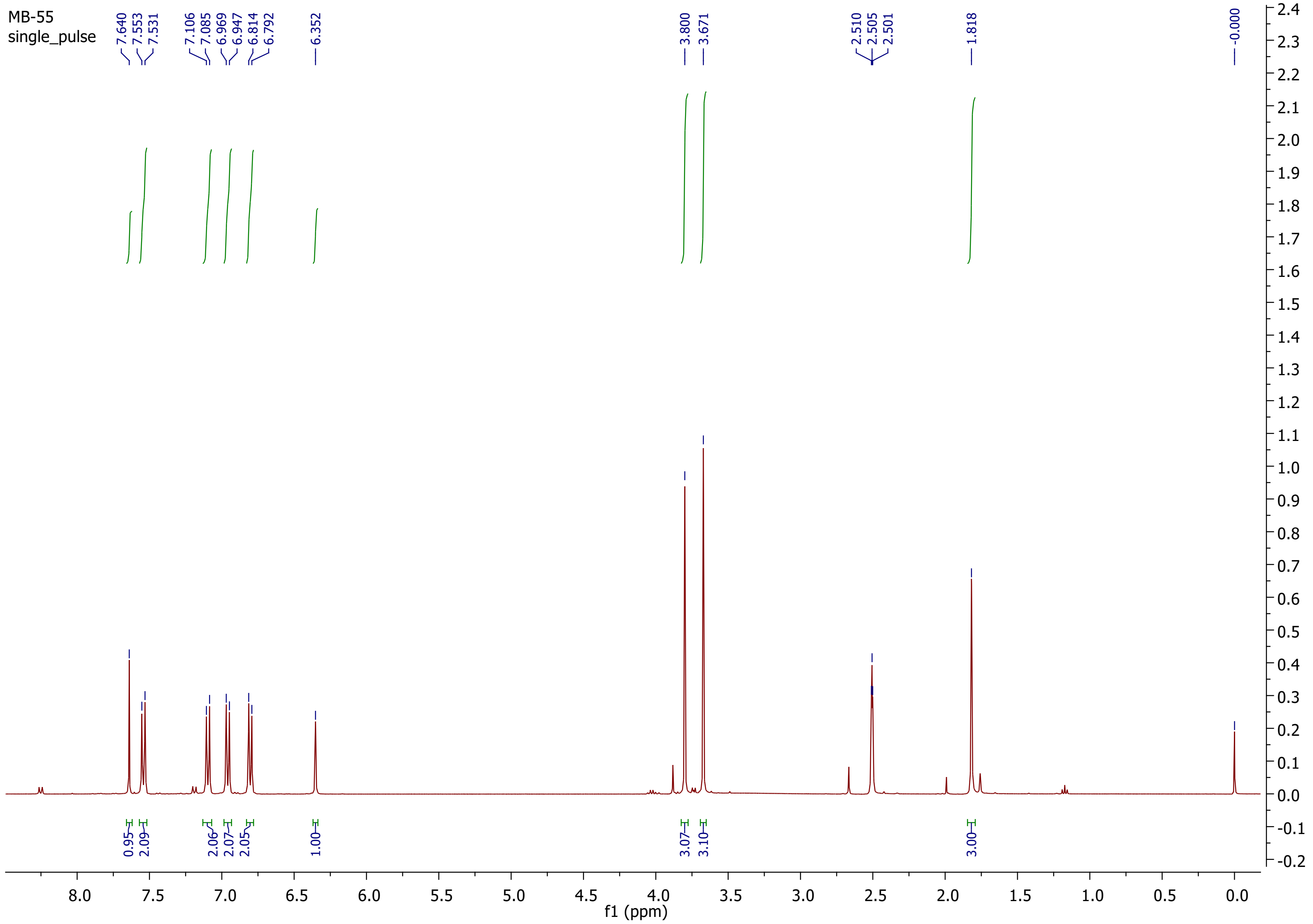

^{13}C NMR of (4''-Methoxyphenyl)(7-(4'-methoxyphenyl)-5-methyl-4,7-dihydro-[1,2,4]triazolo[1,5-*a*]pyrimidin-6-yl)methanone (5l)

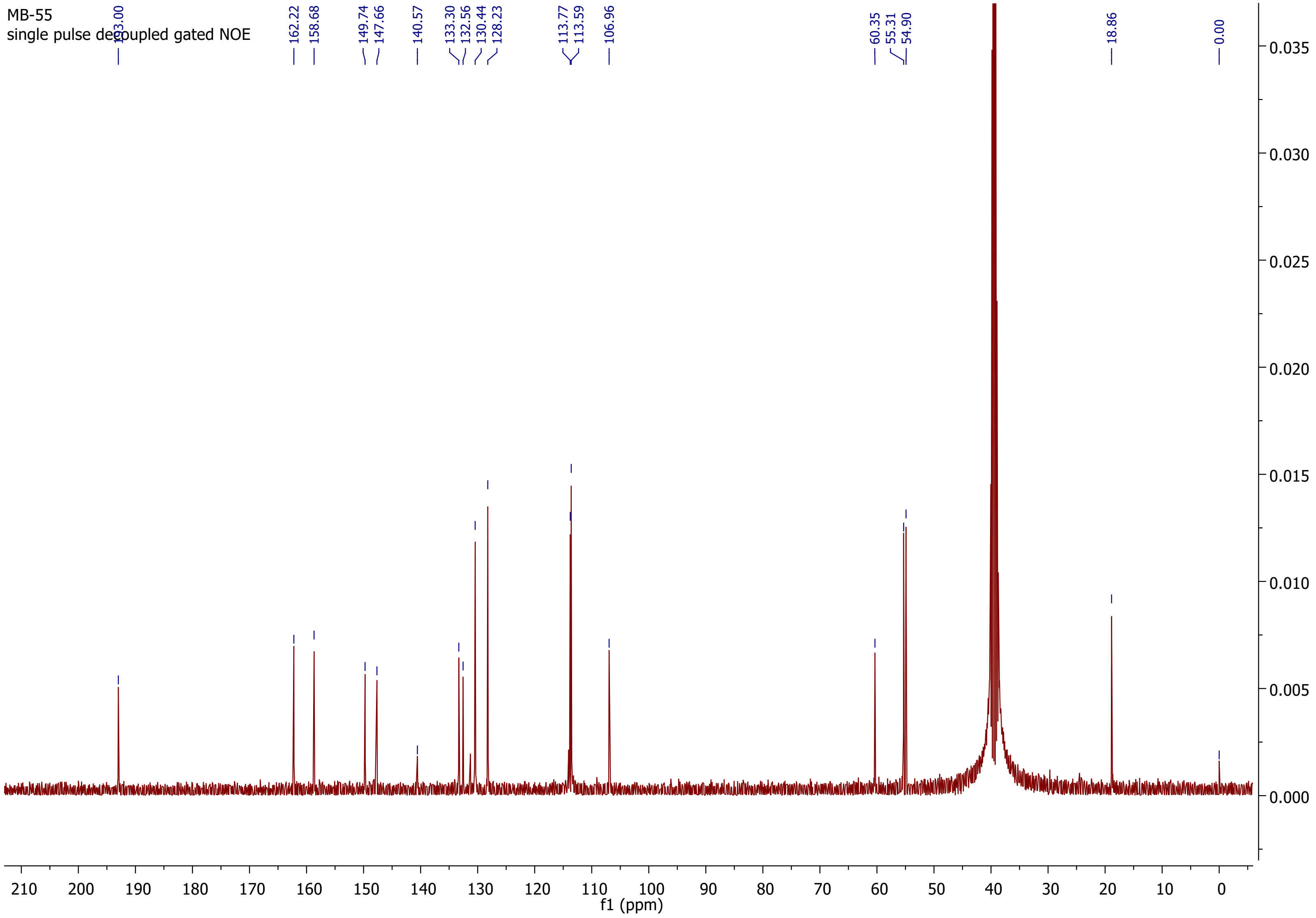

^{1}H NMR of (3''-Methoxyphenyl)(7-(4'-methoxyphenyl)-5-methyl-4,7-dihydro-[1,2,4]triazolo[1,5-*a*]pyrimidin-6-yl)methanone (5m)

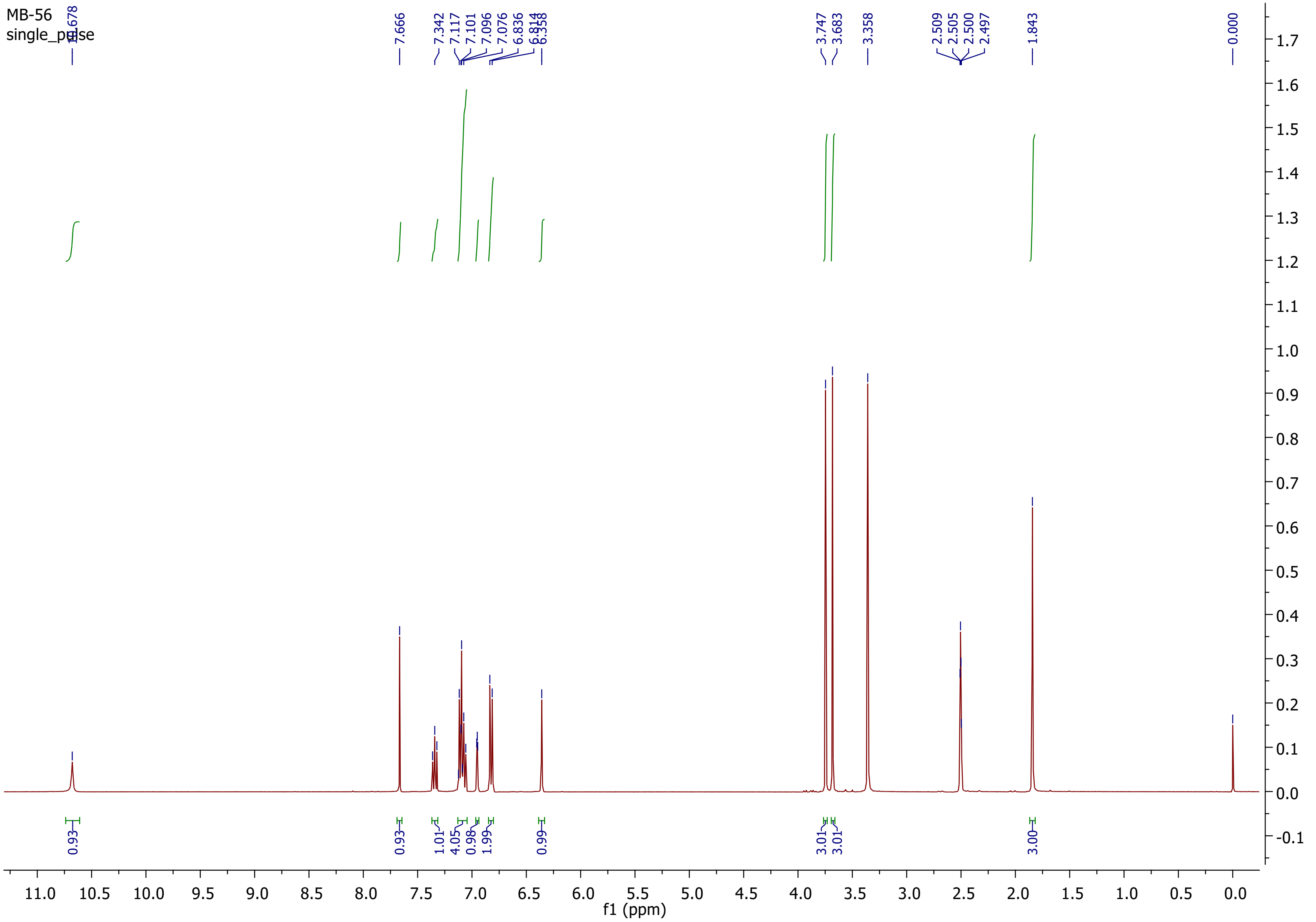

^{13}C NMR of (3''-Methoxyphenyl)(7-(4'-methoxyphenyl)-5-methyl-4,7-dihydro-[1,2,4]triazolo[1,5-*a*]pyrimidin-6-yl)methanone (5m)

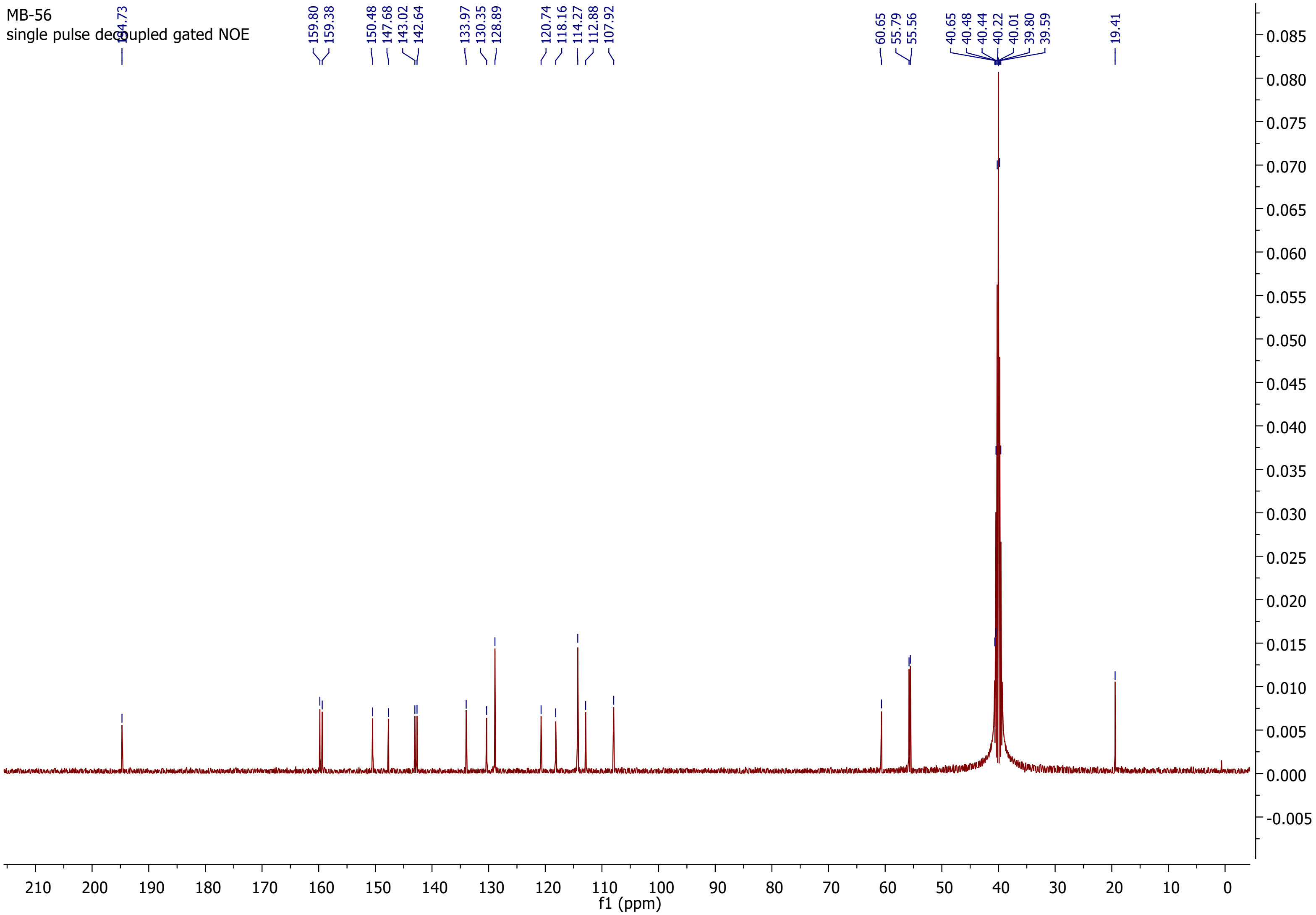

HRMS of (3''-Methoxyphenyl)(7-(4'-methoxyphenyl)-5-methyl-4,7-dihydro-[1,2,4]triazolo[1,5-*a*]pyrimidin-6-yl)methanone (5m)

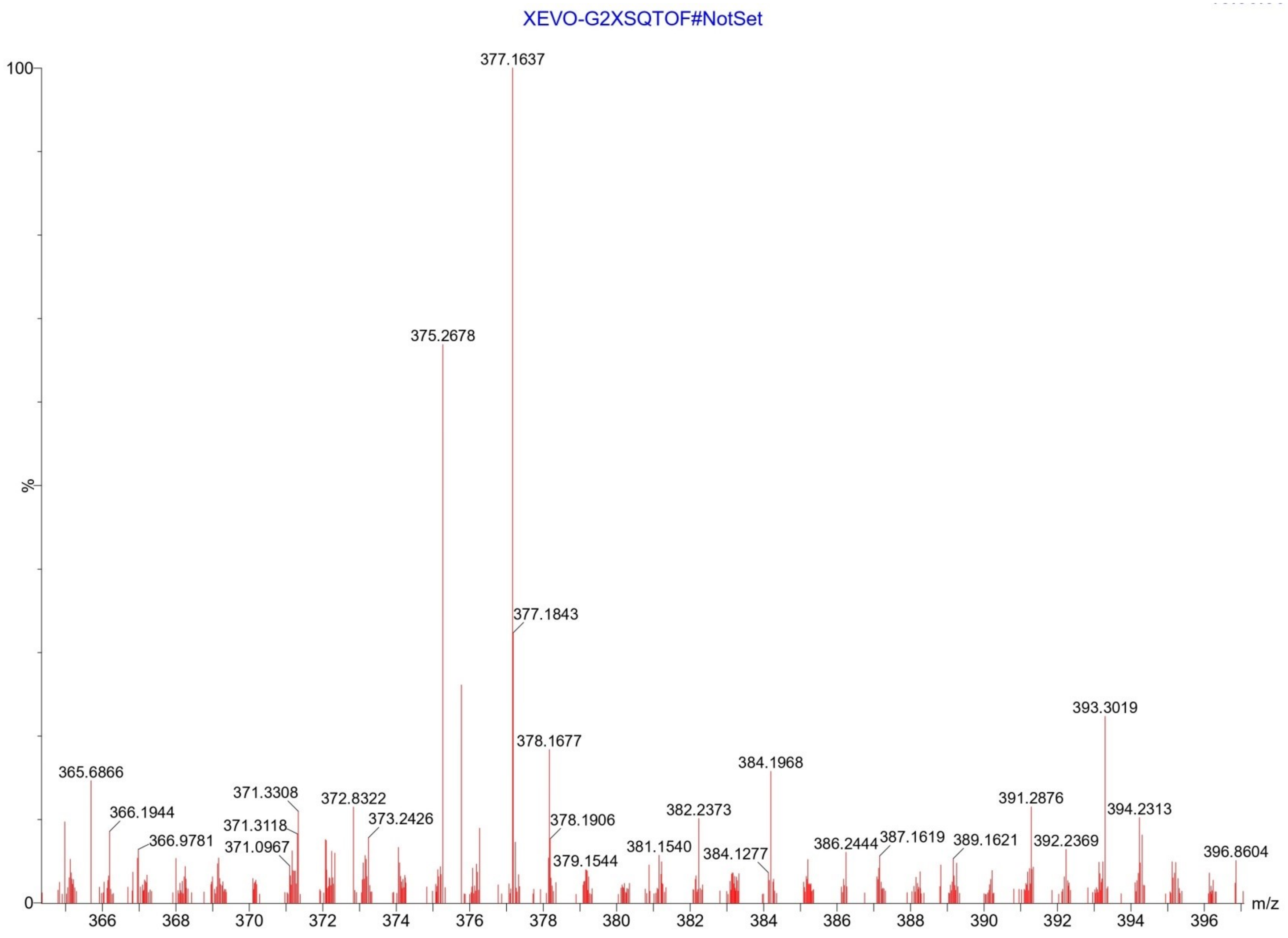

^{1}H NMR of (7-(4'-Methoxyphenyl)-5-methyl-4,7-dihydro-[1,2,4]triazolo[1,5-*a*]pyrimidin-6-yl)(thiophen-2''-yl)methanone (5n)

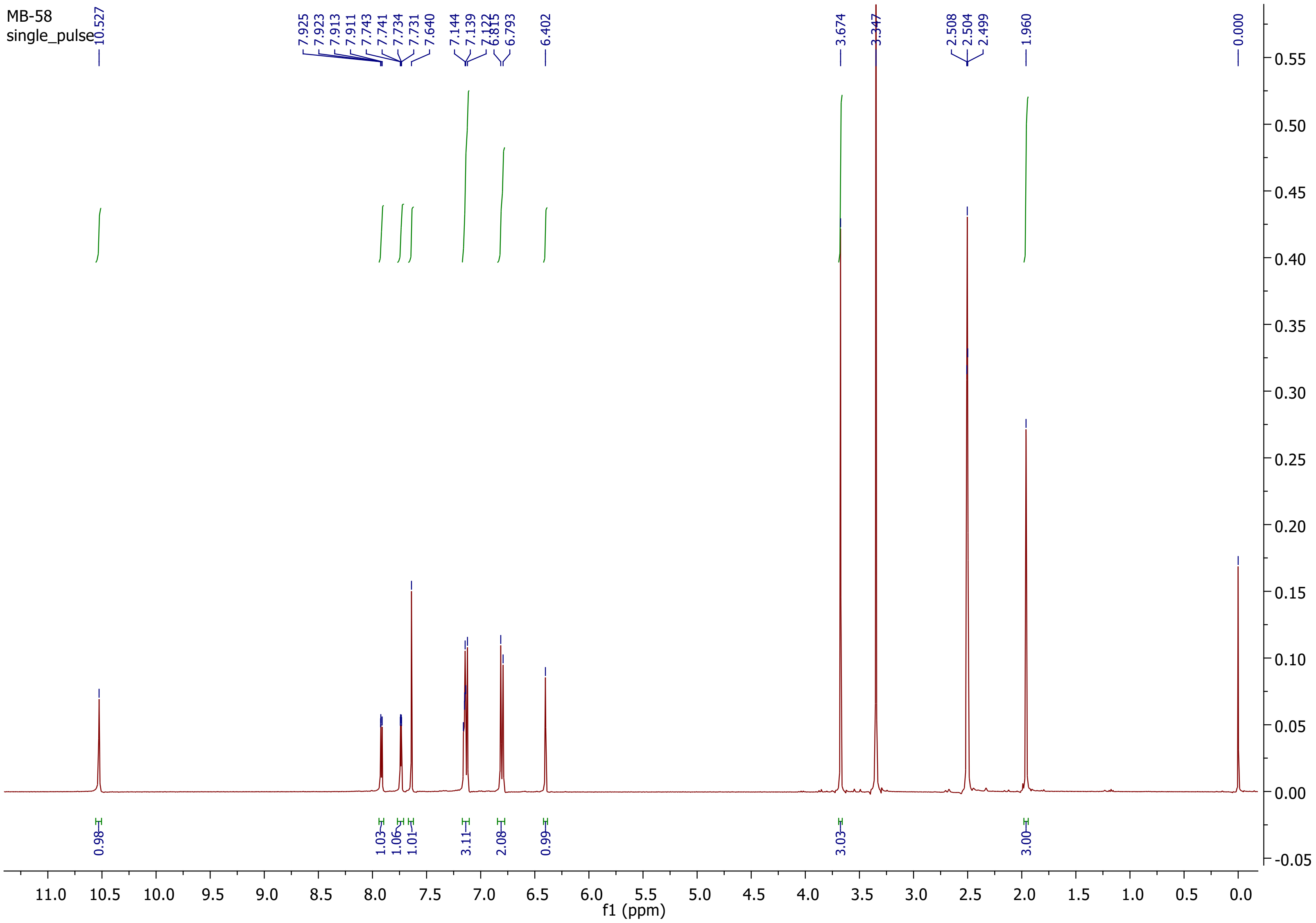

^{13}C NMR of (7-(4'-Methoxyphenyl)-5-methyl-4,7-dihydro-[1,2,4]triazolo[1,5-*a*]pyrimidin-6-yl)(thiophen-2''-yl)methanone (5n)

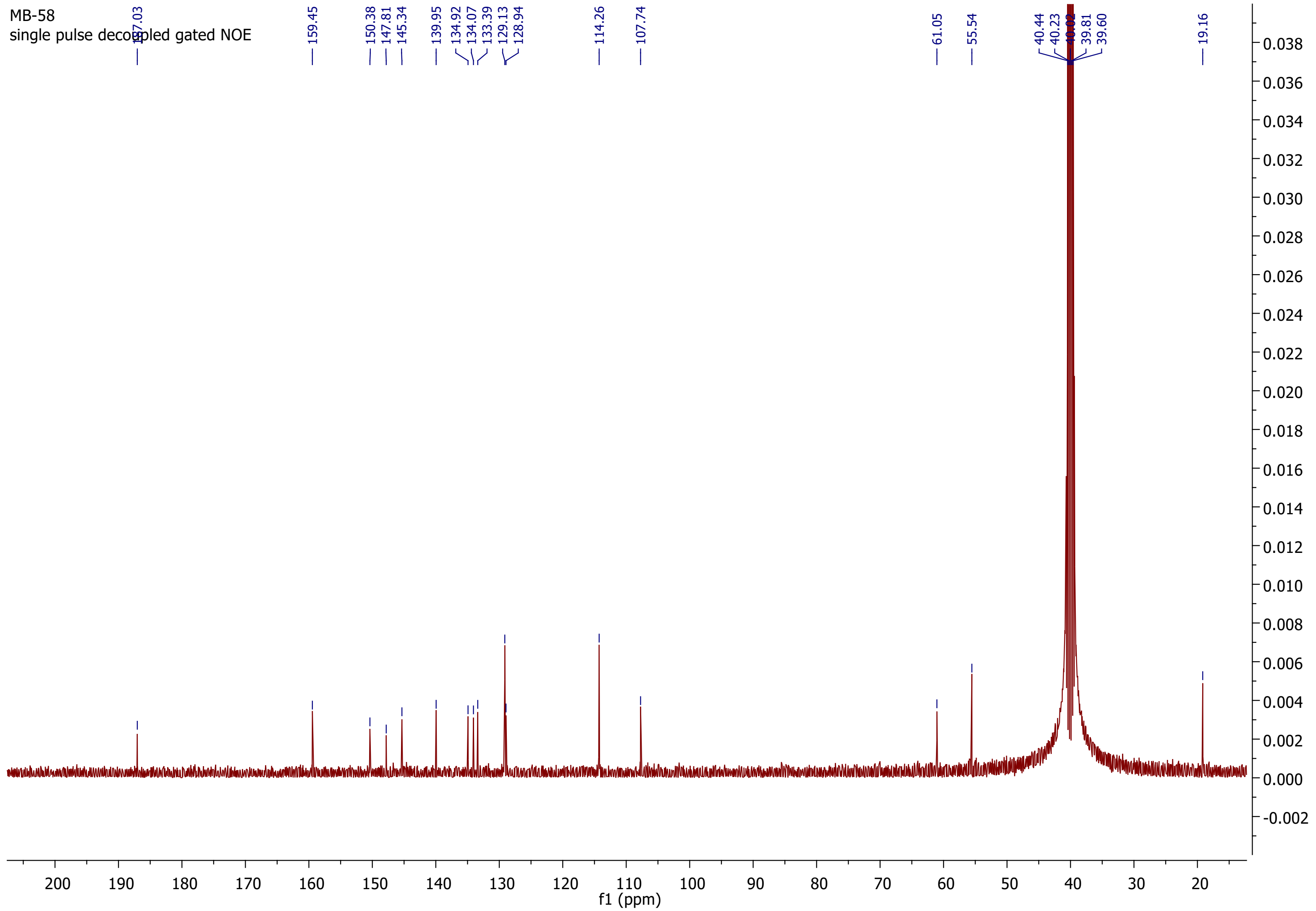

^{1}H NMR of (5-Methyl-7-(4'-nitrophenyl)-4,7-dihydro-[1,2,4]triazolo[1,5-*a*]pyrimidin-6-yl)(phenyl)methanone (5o)

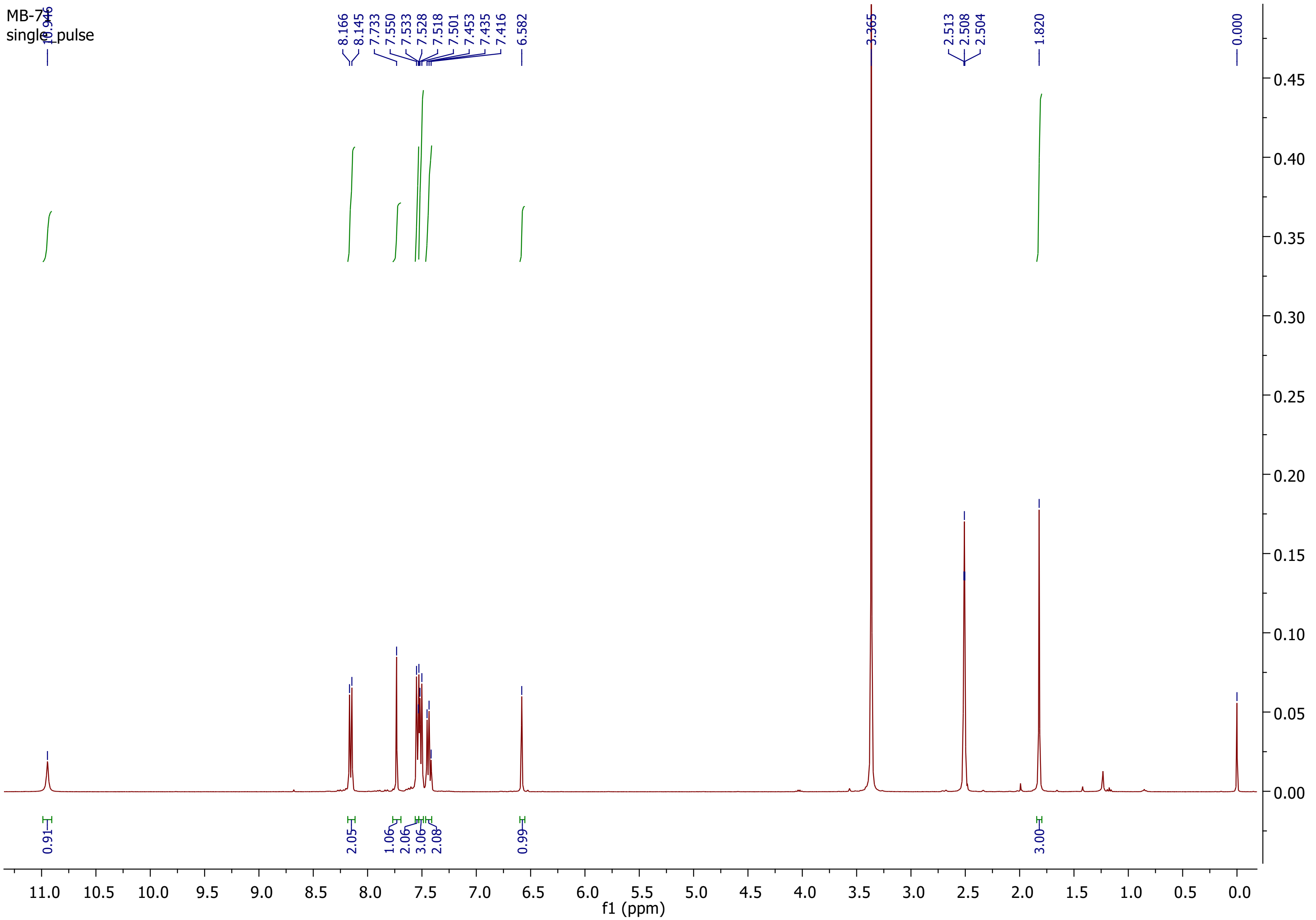

^{13}C NMR of (5-Methyl-7-(4'-nitrophenyl)-4,7-dihydro-[1,2,4]triazolo[1,5-*a*]pyrimidin-6-yl)(phenyl)methanone (5o)

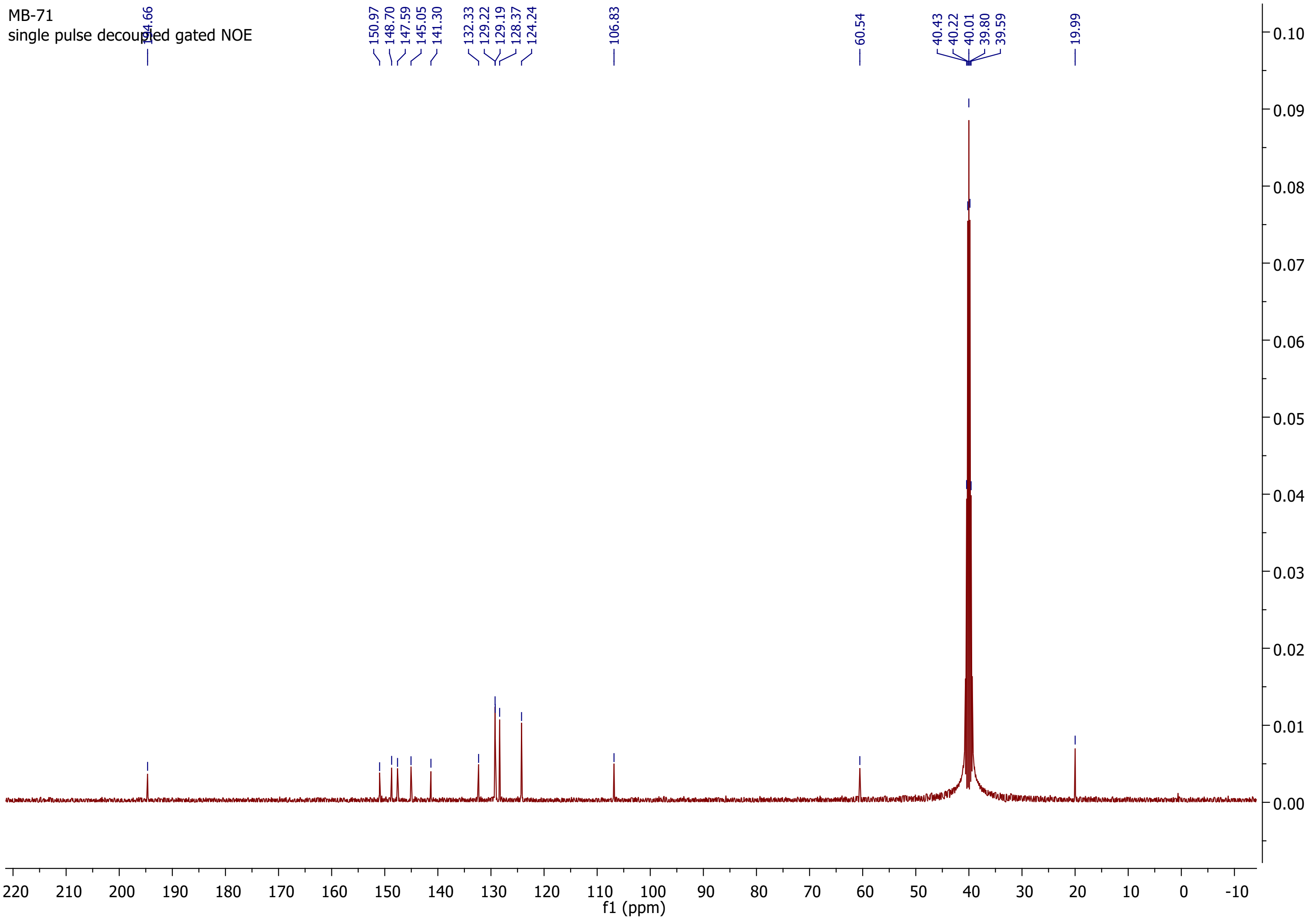

^{1}H NMR of (4''-Fluorophenyl)(5-methyl-7-(4'-nitrophenyl)-4,7-dihydro-[1,2,4]triazolo[1,5-*a*]pyrimidin-6-yl)methanone (5p)

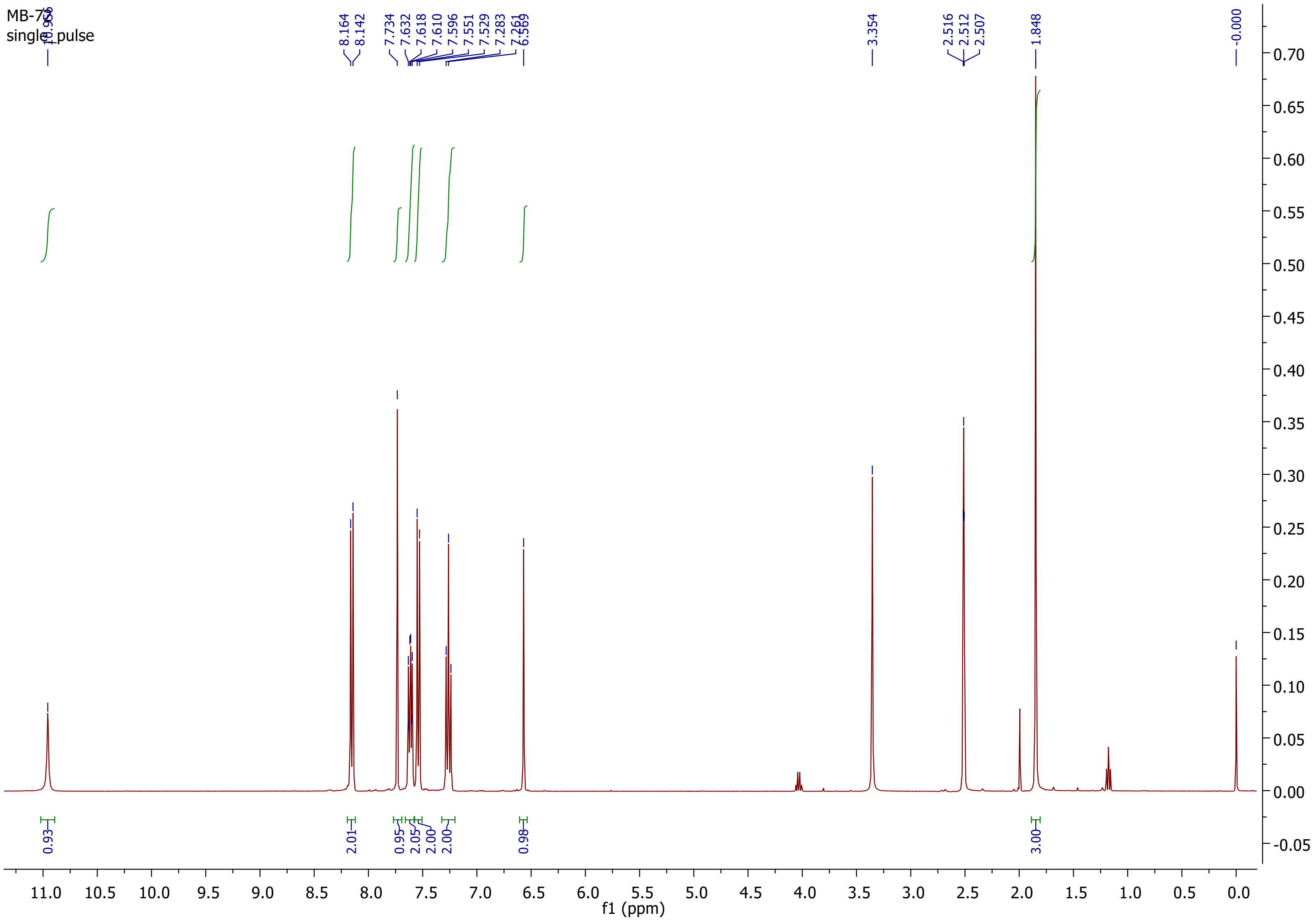

^{13}C NMR of (4''-Fluorophenyl)(5-methyl-7-(4'-nitrophenyl)-4,7-dihydro-[1,2,4]triazolo[1,5-*a*]pyrimidin-6-yl)methanone (5p)

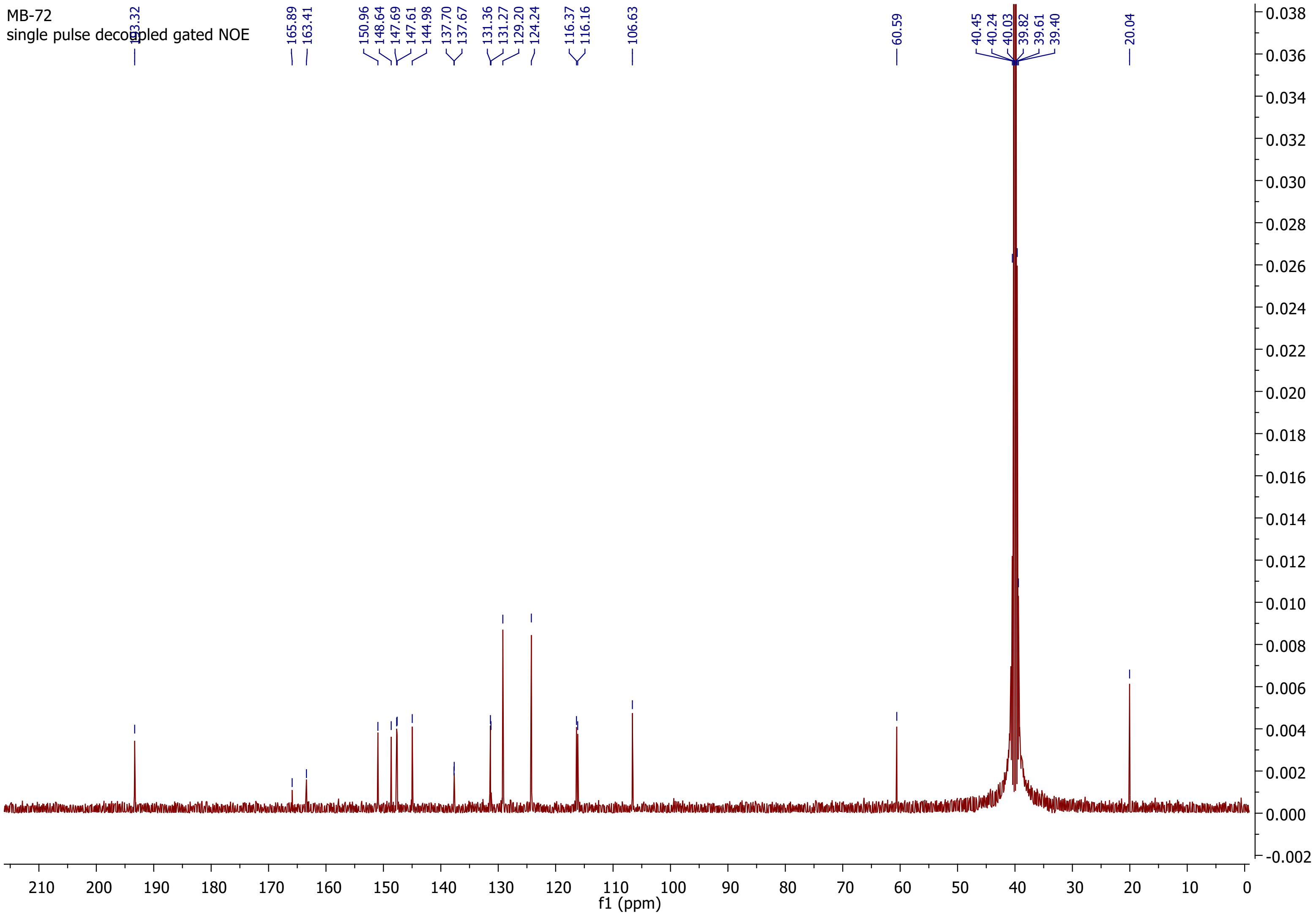

^{13}C NMR of (4''-Chlorophenyl)(5-methyl-7-(4'-nitrophenyl)-4,7-dihydro-[1,2,4]triazolo[1,5-*a*]pyrimidin-6-yl)methanone (5q)

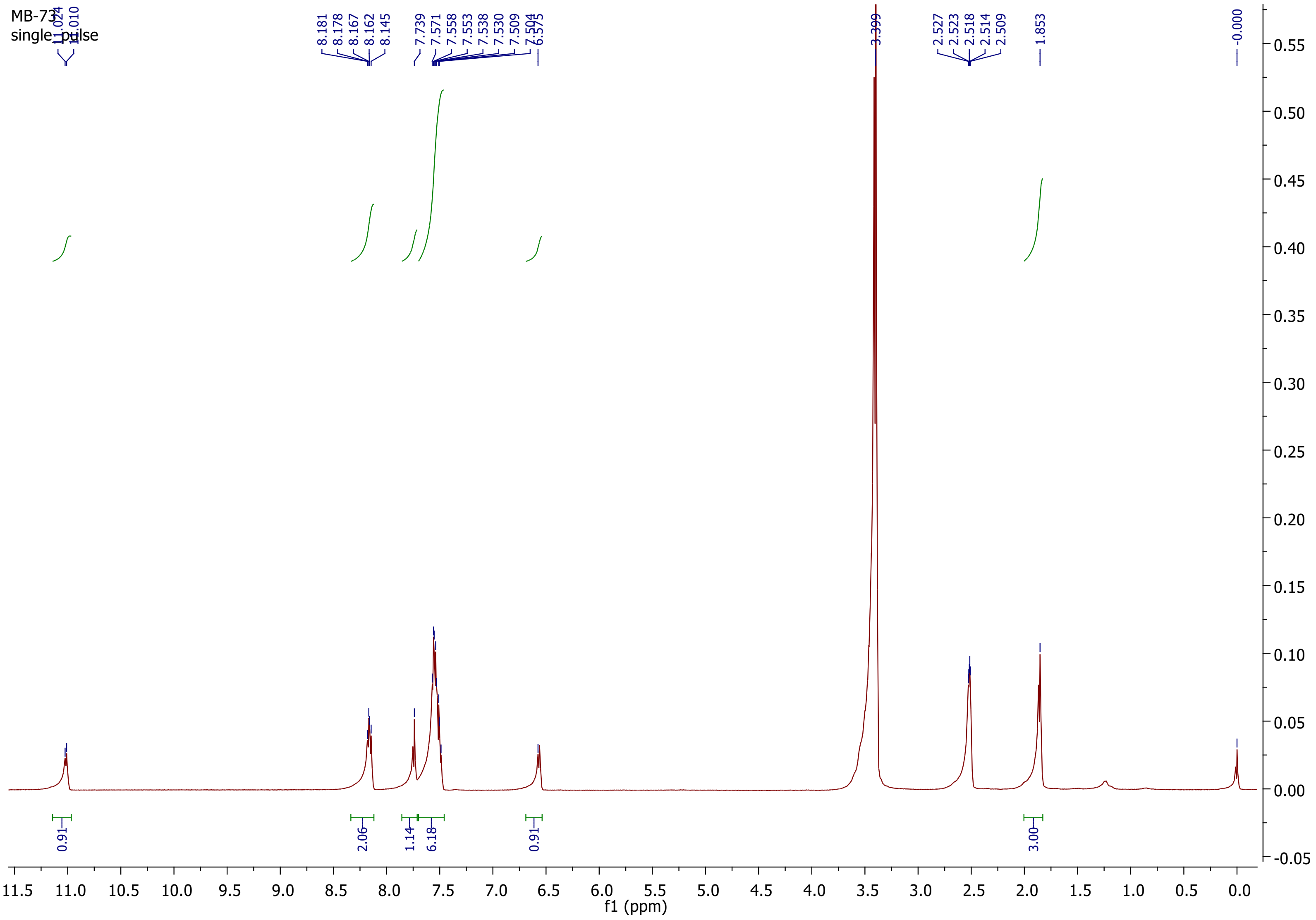

^{13}C NMR of (4''-Chlorophenyl)(5-methyl-7-(4'-nitrophenyl)-4,7-dihydro-[1,2,4]triazolo[1,5-*a*]pyrimidin-6-yl)methanone (5q)

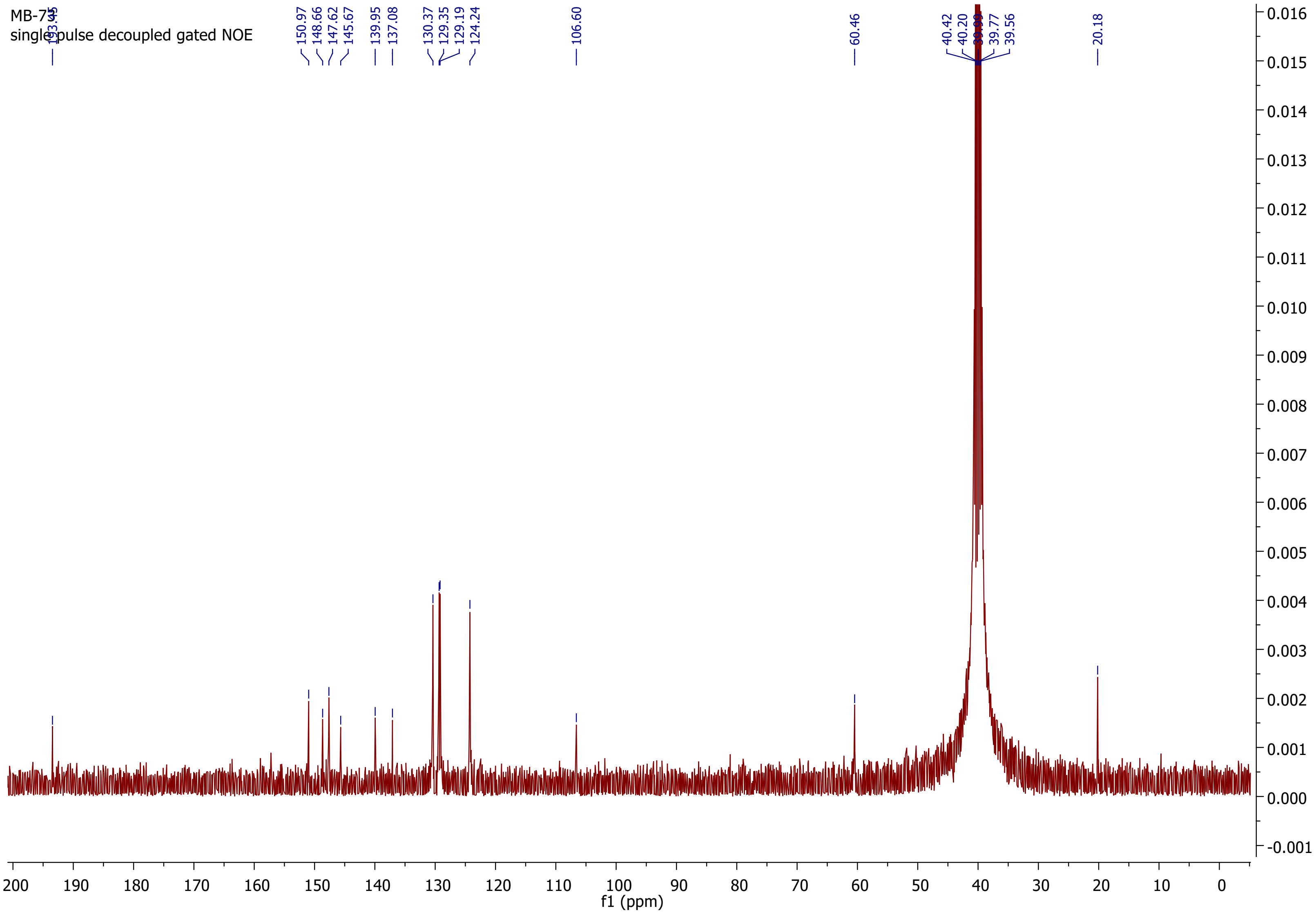

^{1}H NMR of (4''-Bromophenyl)(5-methyl-7-(4'-nitrophenyl)-4,7-dihydro-[1,2,4]triazolo[1,5-*a*]pyrimidin-6-yl)methanone (5r)

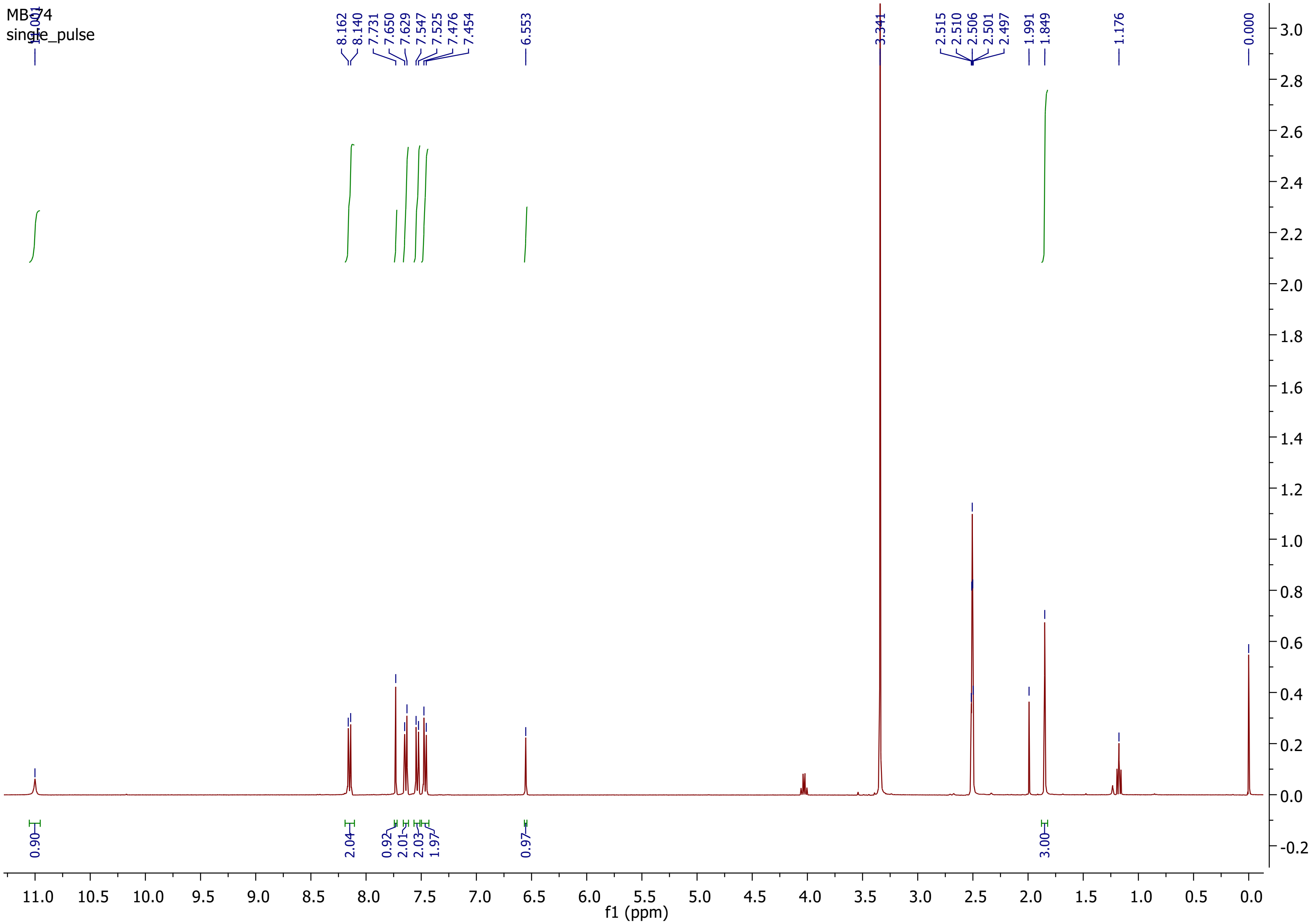

^{13}C NMR of (4''-Bromophenyl)(5-methyl-7-(4'-nitrophenyl)-4,7-dihydro-[1,2,4]triazolo[1,5-*a*]pyrimidin-6-yl)methanone (5r)

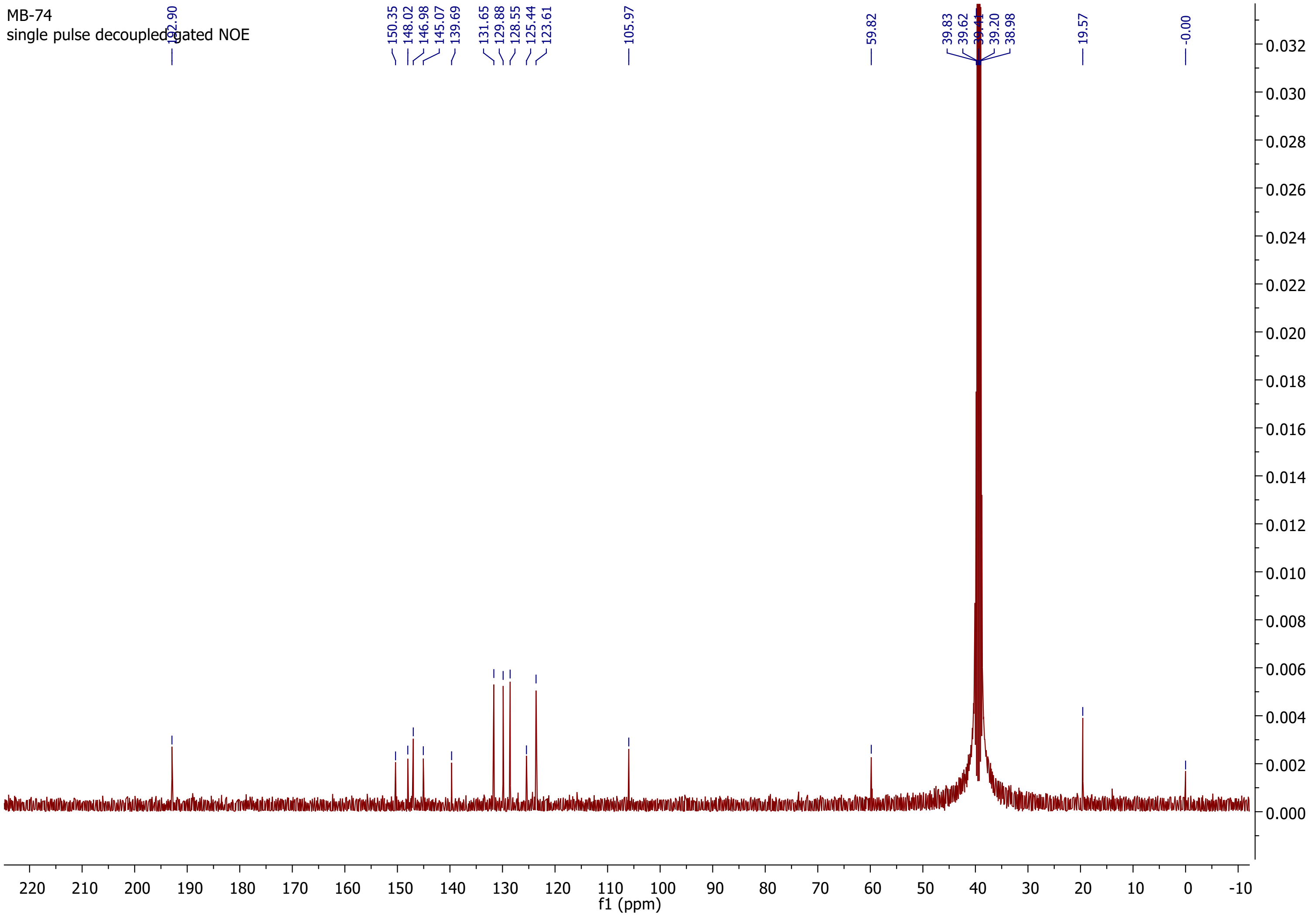

^{1}H NMR of (4''-Methoxyphenyl)(5-methyl-7-(4'-nitrophenyl)-4,7-dihydro-[1,2,4]triazolo[1,5-*a*]pyrimidin-6-yl)methanone (5s)

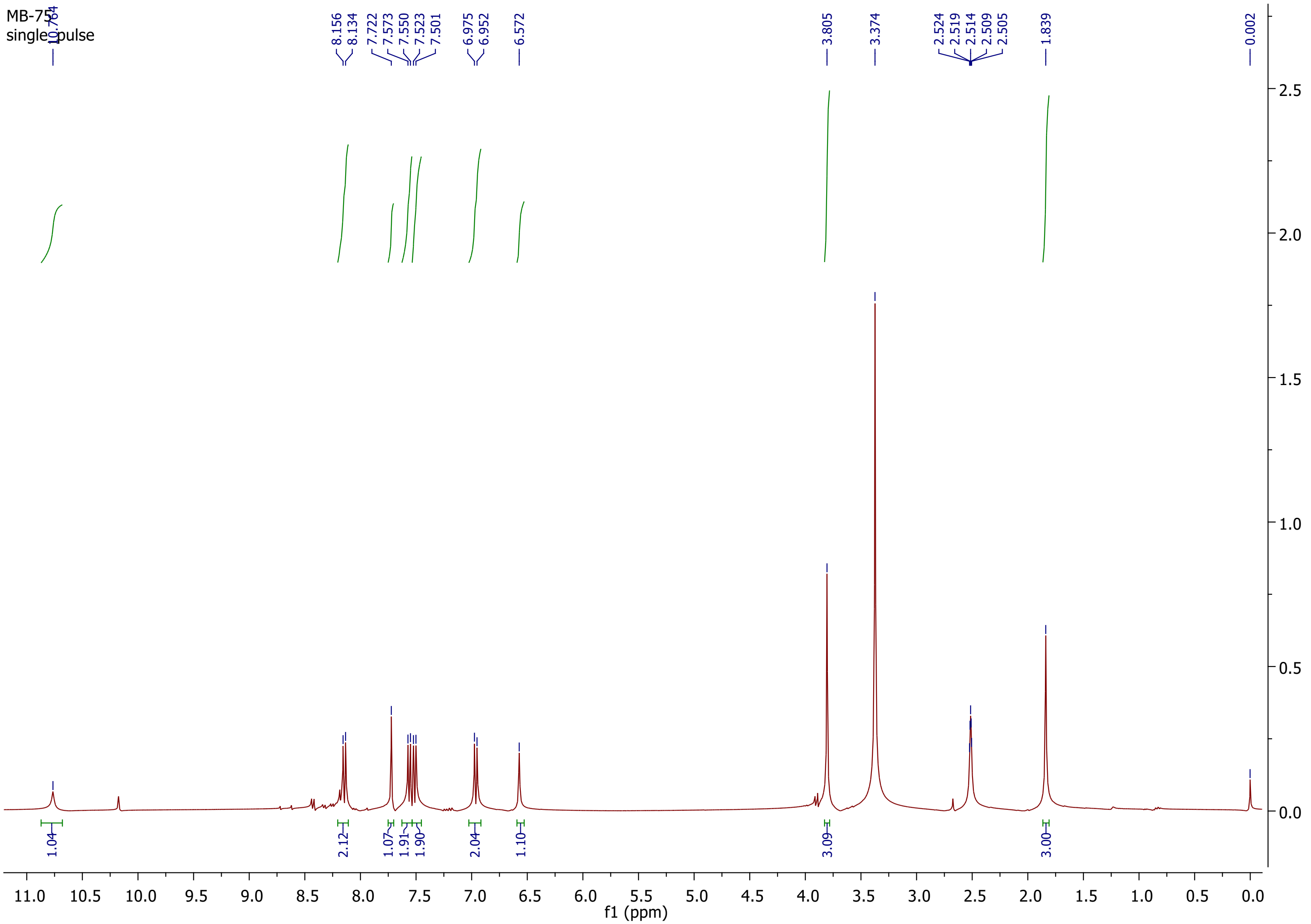

^{13}C NMR of (4''-Methoxyphenyl)(5-methyl-7-(4'-nitrophenyl)-4,7-dihydro-[1,2,4]triazolo[1,5-*a*]pyrimidin-6-yl)methanone (5s)

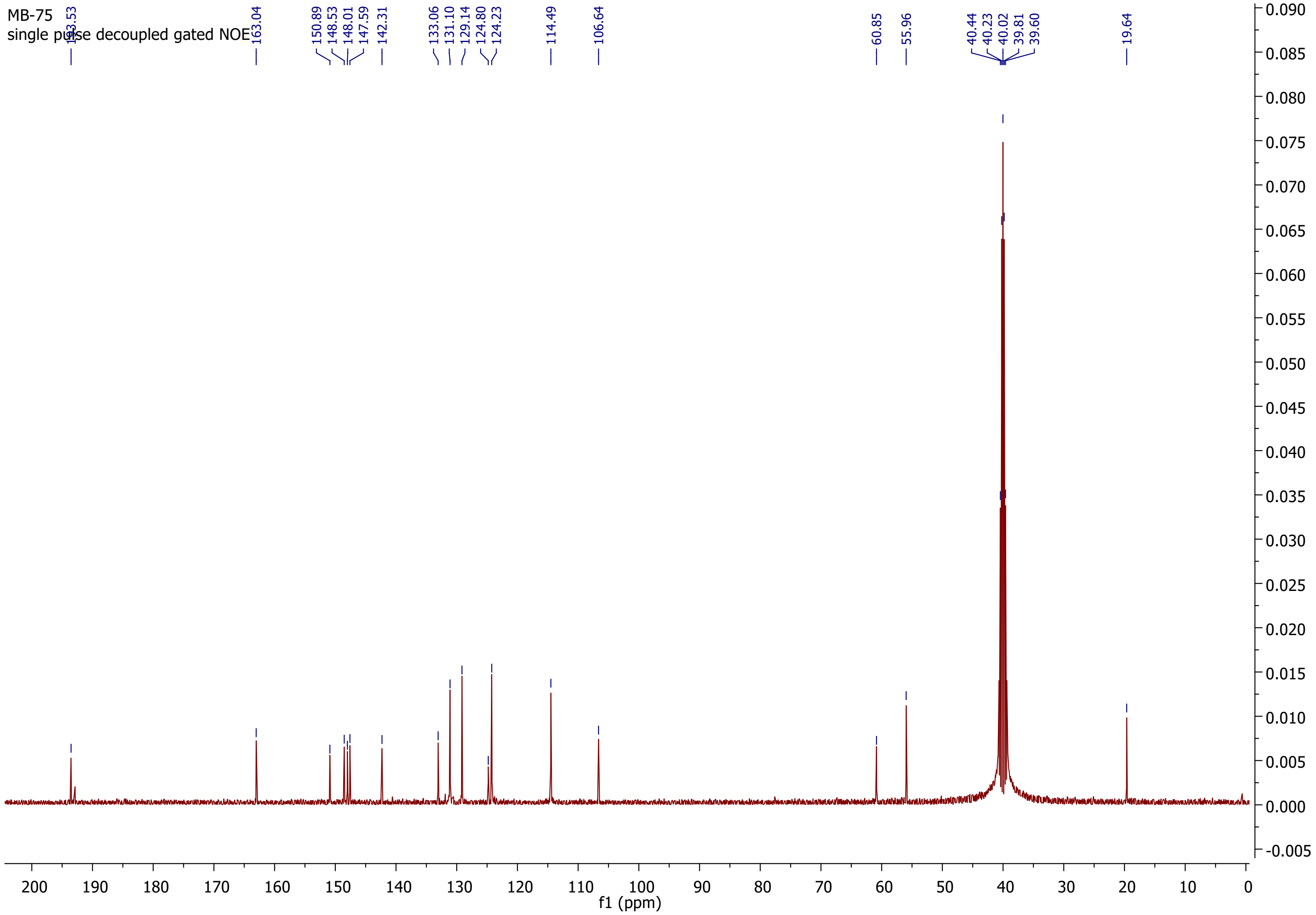

^{1}H NMR of (3''-Methoxyphenyl)(5-methyl-7-(4'-nitrophenyl)-4,7-dihydro-[1,2,4]triazolo[1,5-*a*]pyrimidin-6-yl)methanone (5t)

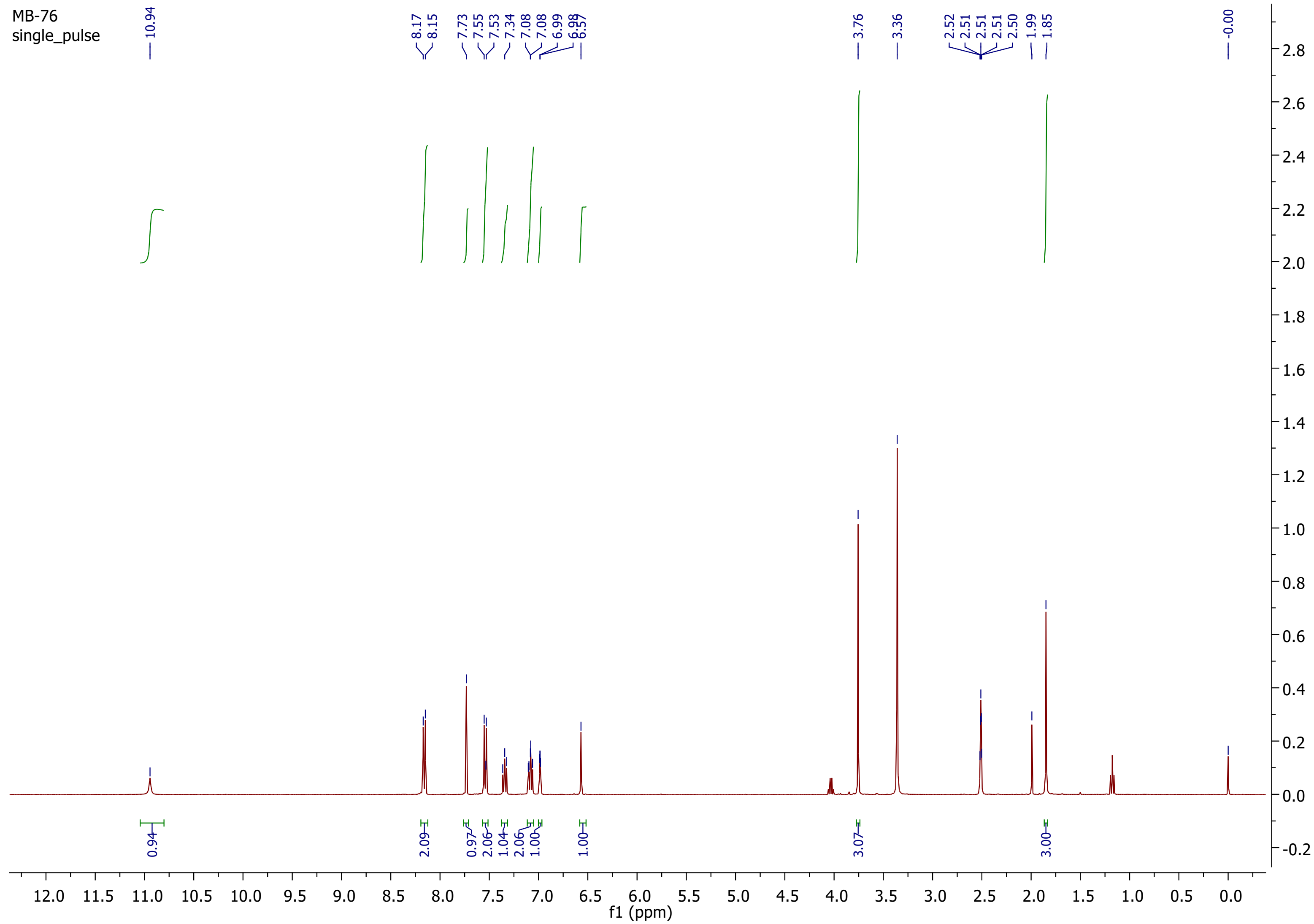

^{13}C NMR of (3''-Methoxyphenyl)(5-methyl-7-(4'-nitrophenyl)-4,7-dihydro-[1,2,4]triazolo[1,5-*a*]pyrimidin-6-yl)methanone (5t)

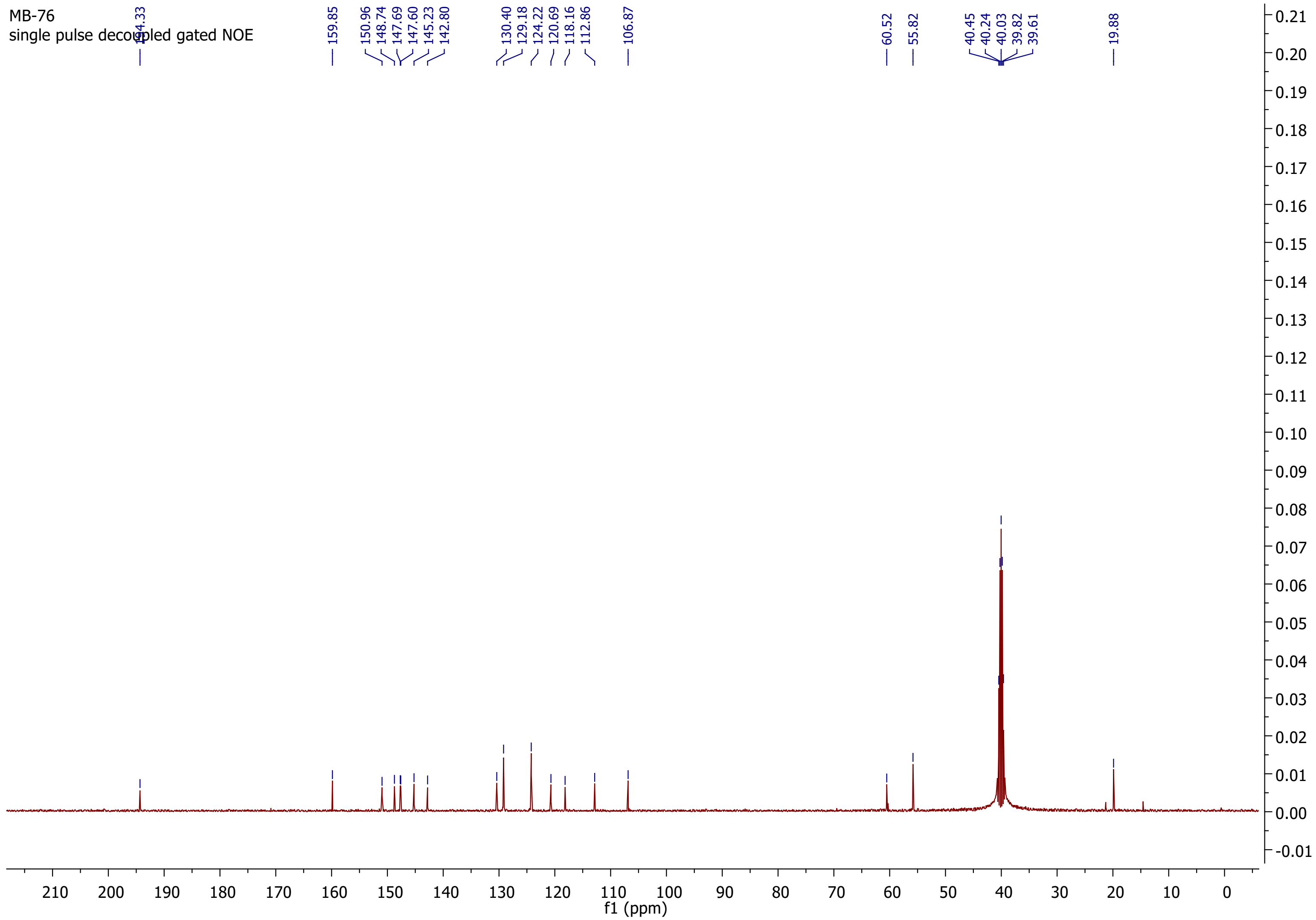

HRMS of (3''-Methoxyphenyl)(5-methyl-7-(4'-nitrophenyl)-4,7-dihydro-[1,2,4]triazolo[1,5-*a*]pyrimidin-6-yl)methanone (5t)

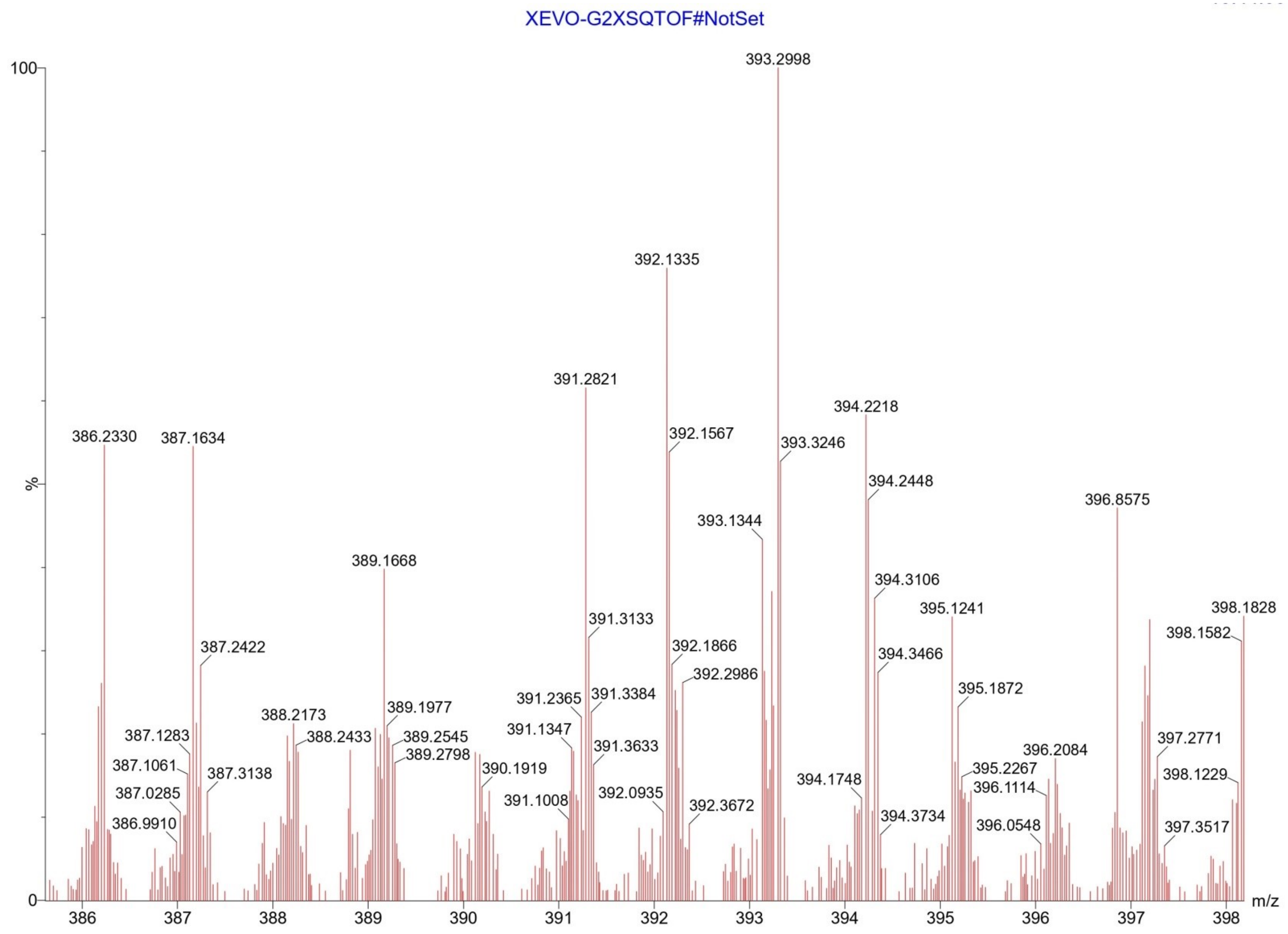

^{1}H NMR of 5-Methyl-7-(4'-nitrophenyl)-4,7-dihydro-[1,2,4]triazolo[1,5-*a*]pyrimidin-6-yl)(thiophen-2''-yl)methanone (5u)

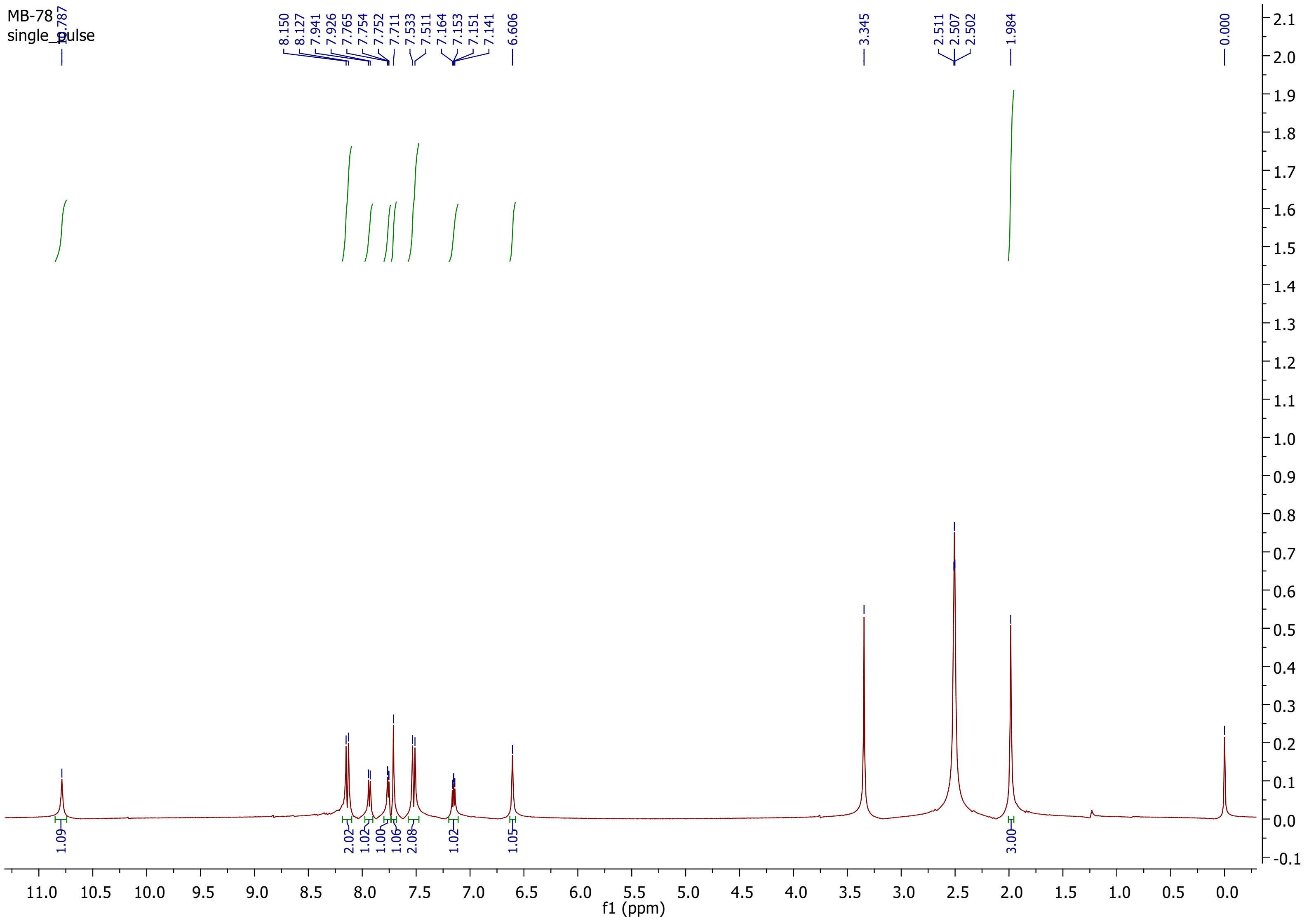

^{13}C NMR of 5-Methyl-7-(4'-nitrophenyl)-4,7-dihydro-[1,2,4]triazolo[1,5-*a*]pyrimidin-6-yl)(thiophen-2''-yl)methanone (5u)

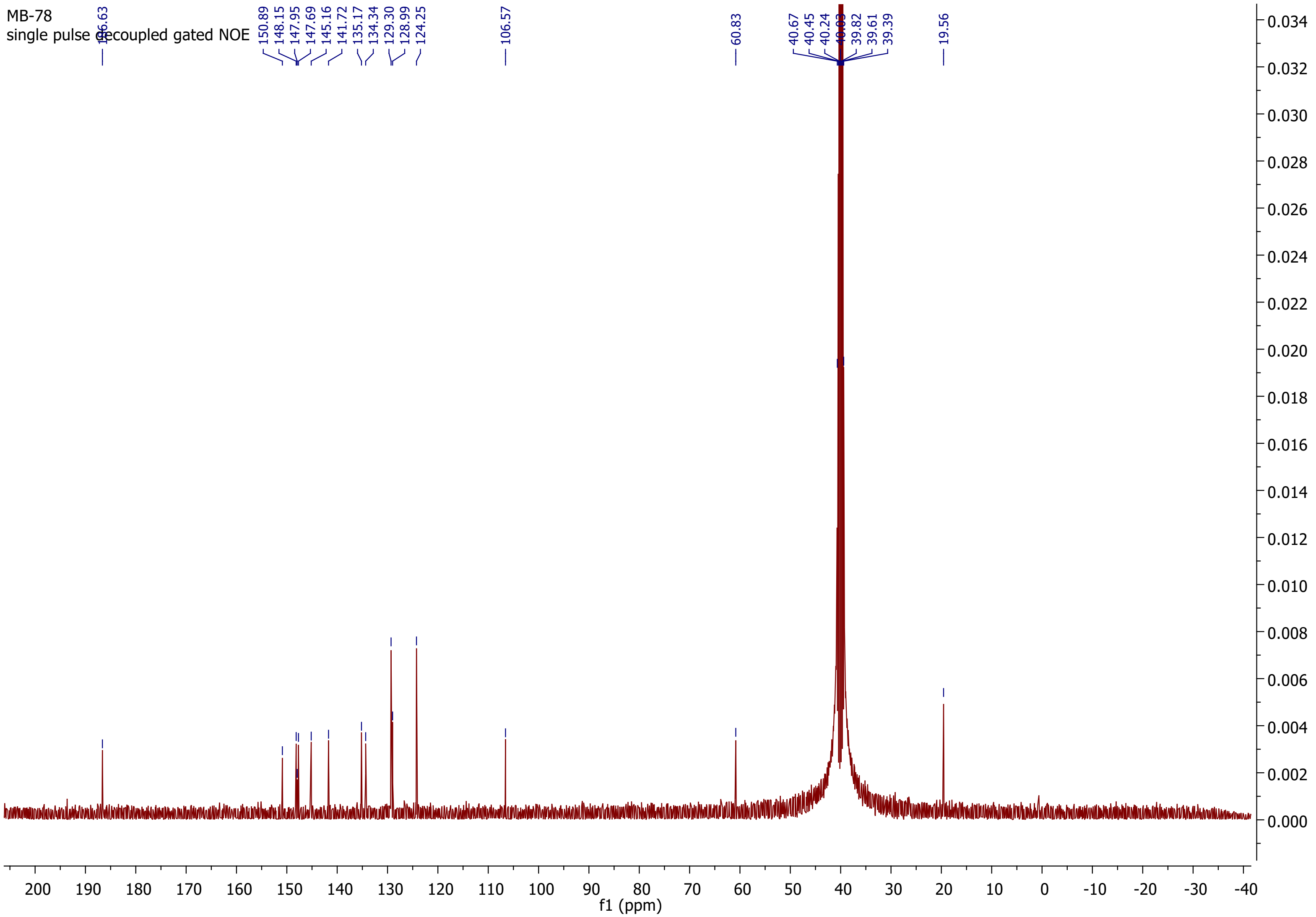

Fig. S1. ^{1}H-^{13}C HMBC NMR of (4''-Methoxyphenyl)(7-(4'-methoxyphenyl)-5-methyl-4,7-dihydro-[1,2,4]triazolo[1,5-*a*]pyrimidin-6-yl)methanone (5l)

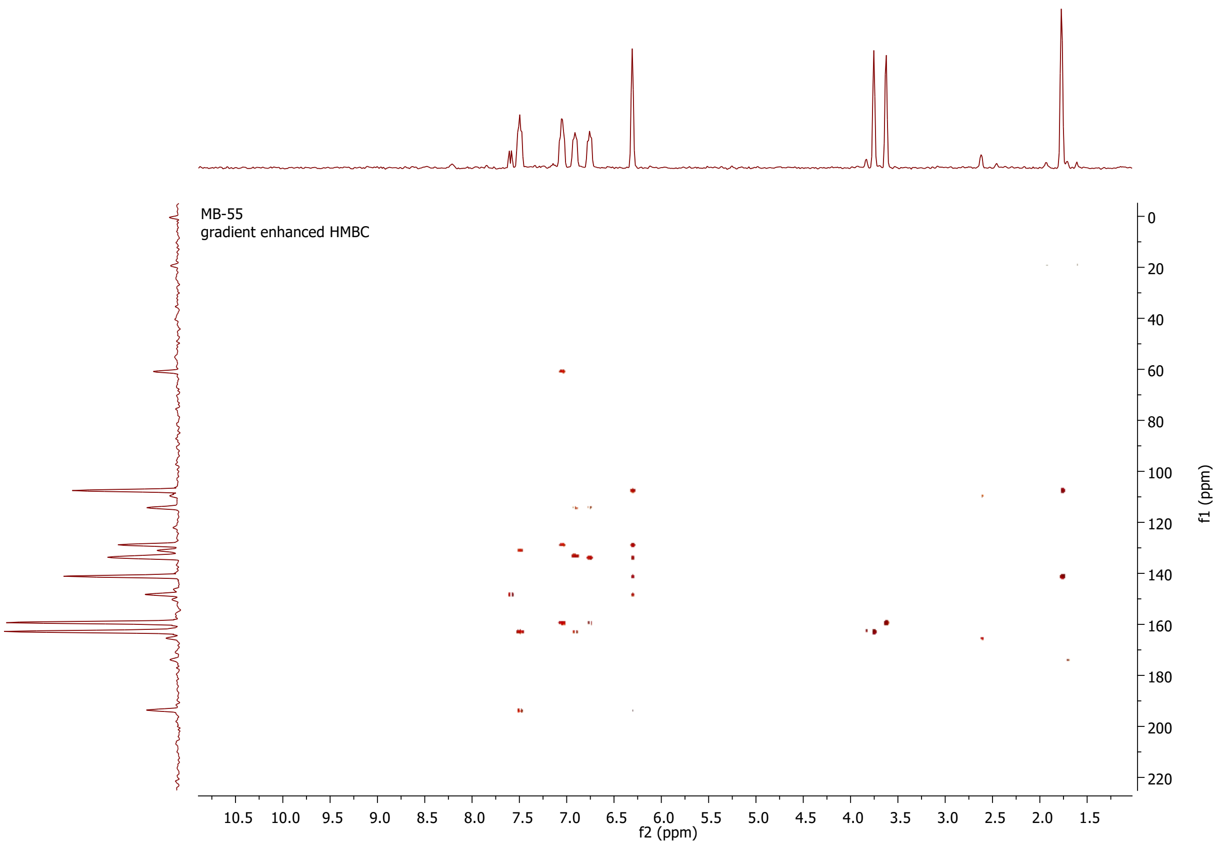

Fig. S2. ^{1}H-^{13}C HSQC NMR of (4''-Methoxyphenyl)(7-(4'-methoxyphenyl)-5-methyl-4,7-dihydro-[1,2,4]triazolo[1,5-*a*]pyrimidin-6-yl)methanone (5l)

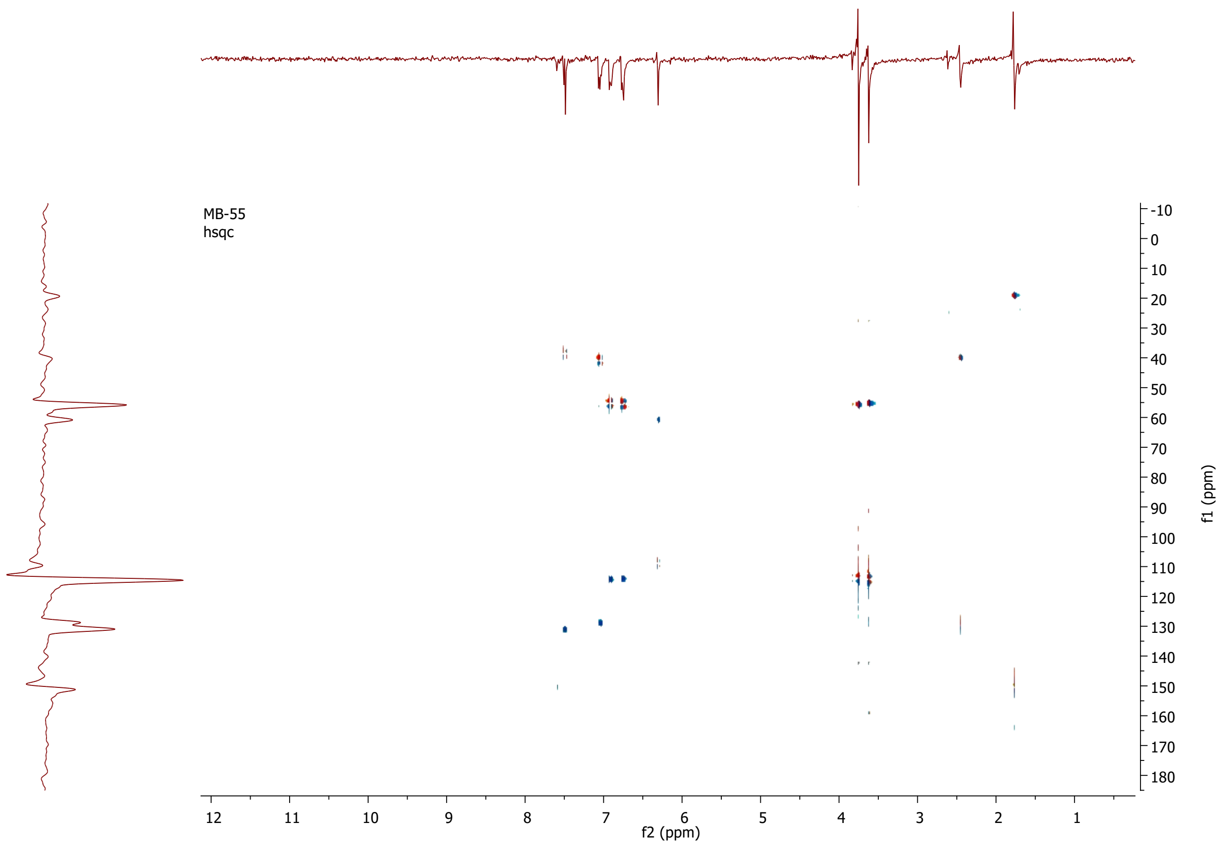

Fig. S3. ^{1}H-^{15}N HMBC NMR of (4''-Methoxyphenyl)(7-(4'-methoxyphenyl)-5-methyl-4,7-dihydro-[1,2,4]triazolo[1,5-*a*]pyrimidin-6-yl)methanone (5l)

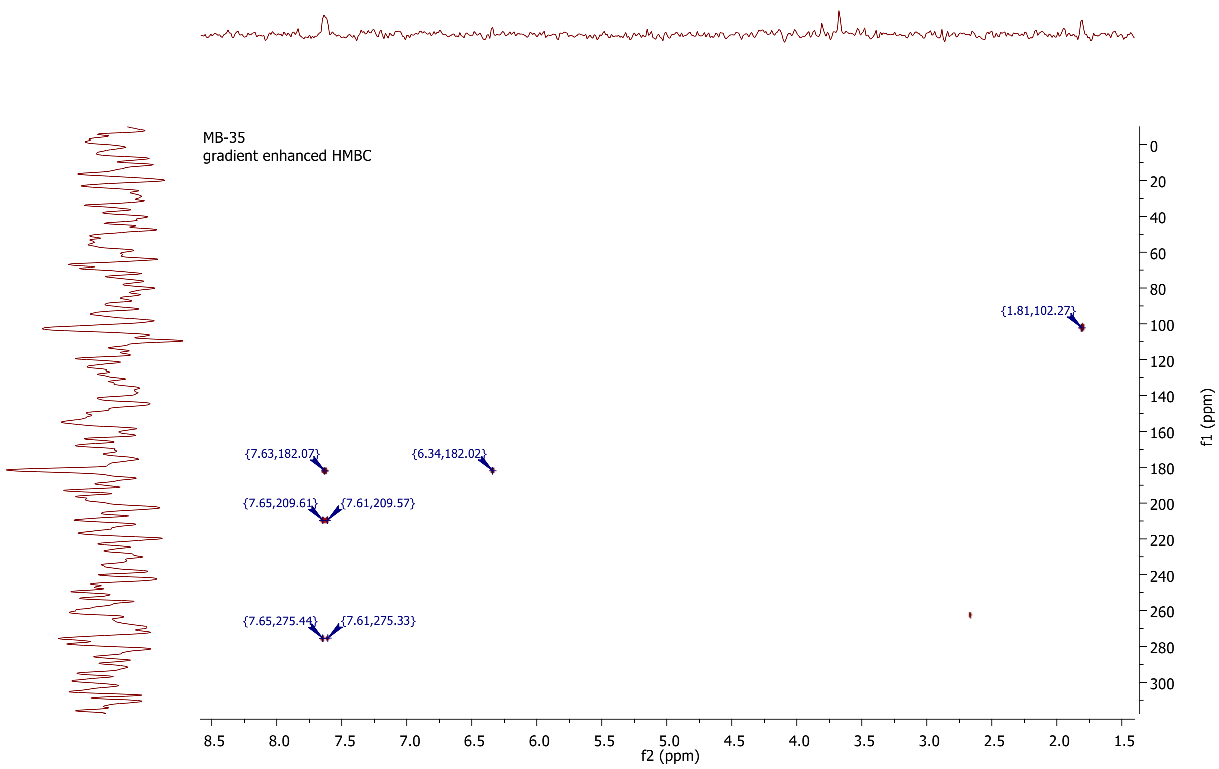

Supplement: RA-016-D5RA09673A-s001 [file RA-016-D5RA09673A-s001.pdf]
